# Molecular Tumor Boards clinical impact on patient care and structural features: A systematic review and meta-analysis

**DOI:** 10.1371/journal.pmed.1005125

**Published:** 2026-06-09

**Authors:** Luigi Russo, Erika Giacobini, Nicolò Lentini, Tommaso Osti, Maud Kamal, Stefania Boccia, Roberta Pastorino

**Affiliations:** 1 Section of Hygiene, Department of Life Sciences and Public Health, Università Cattolica del Sacro Cuore, Rome, Italy; 2 IHU PRISM National PRecISion Medicine Center in Oncology Gustave Roussy Cancer Campus, Villejuif, France; 3 Department of Woman and Child Health, Fondazione Policlinico Universitario A. Gemelli IRCCS, Rome, Italy; undefined, ITALY

## Abstract

**Background:**

Molecular Tumor Boards (MTBs) bring together multidisciplinary experts to translate genomic data into clinical decisions in oncology, however, their overall clinical impact remains unclear. The aim of this systematic review is to assess the clinical impact of MTB-recommended therapies on patients with cancer outcomes.

**Methods and findings:**

In this systematic review and meta-analysis, we searched PubMed, Embase, Scopus, and CENTRAL up to July 2025. We included studies of any design, both single-arm studies and studies with a comparator group, that reported the clinical impact of MTBs in patients who received MTB-guided therapy. Meta-analyses were performed separately by study design, using hazard ratios (HRs) for overall survival (OS) and progression-free survival (PFS), relative risks (RRs) for objective response rate (ORR) and disease control rate (DCR), and pooled proportions for PFS ratio ≥1.3. All meta-analyses were conducted using random-effects models based on the inverse variance method. We evaluated the risk of bias using the RoB 2.0 for RCTs and ROBINS-I for non-randomized studies.

From 6,846 records, 78 studies (9,195 patients; 4,569 treated per MTB recommendations) were included. MTB-guided therapies were associated with reduced risk of death (HR 0.87; 95% CI [0.76, 1.01]; *p* = 0.069; *I*^2^ = 0.0% in RCTs; 0.62 in retrospective studies) and disease progression (HR 0.73; 95% CI [0.64, 0.84]; *p* < 0.001; *I*^2^ = 0.0% in RCTs; 0.63 in retrospective studies), as well as improved ORR (RR 1.75; 95% CI [1.24, 2.47]; *p* = 0.001; *I*^2^ = 0.0% in RCTs; 3.32 in retrospective studies) and DCR (RR 1.20; 95% CI [1.03, 1.40]; *p* = 0.018; *I*^2^ = 19.9% in RCTs; 1.65 in retrospective studies). Between 33% and 43% of patients achieved a PFS ratio ≥1.3. While the risk of bias for RCTs was low, except for one study that was rated as having some concerns, the overall risk of bias for non-randomized studies was rated as “serious” in most of the studies (*n* = 54). Limitations include substantial heterogeneity, predominance of non-randomized studies with risk of bias, and limitations in data reporting, which restrict causal inference.

**Conclusions:**

This meta-analysis provides robust evidence from RCTs supporting the clinical benefit of MTBs, although limited for OS. Methodological heterogeneity and study limitations from observational studies warrant cautious interpretation. Future high-quality RCTs and standardized reporting are needed to confirm these findings and guide the integration of MTBs into routine clinical practice and health system strategies.

## Author summary

**Why was this study done?:**

- Molecular Tumor Boards (MTBs) are increasingly used to choose cancer treatments based on the genetic profile of tumors, but their real impact on patient outcomes has remained uncertain.
- Previous evidence came mostly from small, single-center or observational studies, with limited data from randomized trials.
- A comprehensive and critical synthesis of all available evidence was needed to understand whether MTB-guided treatments truly improve patient outcomes.

**What did the researchers do and find?:**

- We conducted a systematic review and meta-analysis of 78 studies including 9,195 patients with cancer, of whom 4,569 received treatments recommended by an MTB.
- High heterogeneity in the organization of MTBs emerged, in terms of different experts composing the board, schedule of meetings, comparison arms, and actionability frameworks used.
- Patients treated according to MTB recommendations experienced better control of their disease, longer progression-free survival, and higher response rates to treatment. However, evidence for overall survival was less certain, with a general improvement clearer in observational studies but less consistent in randomized trials.
- Risk of bias was generally high in non-randomized trial and generally low for randomized controlled trials.

**What do these findings mean?:**

- These results suggest that MTBs can help select more effective treatments and delay cancer progression for many patients.
- The findings support the growing role of MTBs in precision oncology and may inform clinical practice and health policy decisions, especially in systems where access to targeted therapies depends on MTB evaluation.
- However, results should be interpreted with caution since many included studies were observational and had critical limitations, such as differences in how patients were selected and how the studies were conducted, and possibly overestimating the effect.

## Abstract 3

In this systematic review meta-analysis, Nicolò Lentini and colleagues investigate the clinical impact of therapies recommended by Molecular Tumor Boards on patient outcomes.

## Introduction

The advent of precision oncology has transformed the clinical approach to cancer treatment over the past decade. Tumors, once considered uniform entities, are now recognized as highly complex networks of genetic and molecular variations, and treatment strategies are now driven by molecular profiling [1]. Advancements in next-generation sequencing (NGS) and multi-omics technologies, have enabled the large-scale identification of molecular targets and the development of a wide range of innovative cancer treatments, leading clinicians in tailoring therapies to the individual molecular landscape of a patient's tumor [2].

The decreasing costs of molecular profiling [3] and the widespread adoption of these technologies in major hospitals across Europe [4], have all contributed to the rapid expansion of precision medicine [5]. Despite these advancements, challenges remain in the clinical implementation of NGS, including data interpretation and the integration of multi-omics approaches into treatment strategies [6,7].

In this context, Molecular Tumor Boards (MTBs) have been established in hospitals to integrate clinical patient information with genetic and genomic data, alongside traditional tumor characteristics [8]. MTBs work as multidisciplinary teams whose mandate is to translate genomics-driven data into therapeutic recommendations for patients with cancer. This includes assessing the clinical actionability of mutations identified through genomic analysis, evaluating the most appropriate therapies, both approved and off-label, and, when appropriate, referring patients to clinical trials or innovative drug programs [9].

In Europe, the organization and regulatory recognition of MTBs vary across countries. In different health systems, involvement of an MTB is one of the prerequisites for the reimbursement of off-label therapies or access to specific targeted treatments [9]. For example, in Germany, MTBs play a key role in interpreting molecular profiling results and are involved in reimbursement decisions for off-label treatments [10]. Recently, Italy officially established MTBs through Law Decree 30/05/2023, and a new decree is currently under discussion to regulate the reimbursement of recommended therapies [11]. In France, MTBs are being integrated into the 2025 national genomics plan, with their recommendations often required to access off-label targeted therapies. A multidisciplinary genomic framework is currently being developed [12]. Nevertheless, some European countries still lack formal procedures or national guidelines to support and strengthen MTBs.

Available evidence from observational studies suggested a potential clinical benefit of MTBs, particularly in terms of improved patient outcomes [8,13]. However, the current body of evidence remains fragmented and is largely limited to single-center experiences or specific patient cohorts, while randomized controlled trials (RCTs) assessing the clinical efficacy of MTBs remain limited [14].

This systematic review and meta-analysis aim is to assess the clinical efficacy of MTB-recommended therapies on patients with cancer outcomes by including the entire body of evidence available from both observational and randomized studies. We aim to evaluate the quality of evidence in order to identify potential sources of bias to make informed considerations.

## Methods

We previously registered the protocol for this systematic review and meta-analysis on OpenScienceFramework [15]. This systematic review was reported following the *Preferred Reporting Items for Systematic Reviews and Meta-Analyses* (PRISMA) Checklist [16] (S1 Checklist).

### Search strategy

We conducted a preliminary search of PubMed, Web of Science, and Scopus to identify relevant keywords. These keywords were then used to develop a comprehensive search strategy.

We searched for articles published in English and indexed in the following databases: PubMed, Scopus, Web of Science, and the Cochrane Central Register of Controlled Trials (CENTRAL), with no restrictions on publication year. Moreover, we searched on ClinicalTrials.gov for registered clinical studies. The full search strings are provided in Table A in S1 Appendix. The search included all articles published from inception up to May 1, 2025.

The search was further updated after completion of the analyses up to July 1, 2025, to identify any additional high-impact publications published in the meantime; three additional observational studies [17–19] were found to be eligible but were not included in the analyses.

### Study eligibility and selection

We evaluated each article against the following eligibility criteria:

1. Studies published or accepted for publication (i.e., in press) in peer-reviewed journals, with no time restrictions, and written in English.
2. Studies involving patients with cancer of any age and with any cancer types.
3. Primary research studies of any design (RCTs, non-randomized clinical trials, prospective observational studies, and retrospective observational studies) that reported the use of MTBs in clinical decision-making and included at least one measurable clinical outcome, with or without a comparator. We defined the comparator as the absence of a MTB approach (e.g., no evaluation or implementation of MTBs in therapy planning). Platform trials using predefined algorithm-based treatment allocation (e.g., TAPUR [20] and NCI-MATCH [21]) were not considered eligible if they did not include a systematic MTB assessment for all patients as part of treatment assignment, which represented a predefined inclusion criterion for the present review.

We uploaded the articles identified on the Rayyan software platform, where we checked and removed duplicates. Four reviewers (L.R., E.G., M.G.C., T.O.) independently screened the titles and abstracts, with each article assessed by two reviewers. Full texts of the selected articles were retrieved and independently reviewed by two researchers (L.R., E.G.). Disagreements in any phase were resolved through consensus or, if necessary, by a third reviewer (T.O.). Articles that met the eligibility criteria were subsequently included in the systematic review. A rejection log was used (S1 File).

### Data extraction

Three reviewers (L.R., E.G., N.L.) independently performed data extraction, with each article assessed by two reviewers. From each included study, we extracted:

*General information:* title, first author, DOI, journal of publication, year of publication, country.

*Study characteristics:* study design, number of arms, sample size.

*MTBs characteristics:* modality of access to the MTB (eligible patients from the same institution or clinical trial, external or selected patients), composition of the board, frequency of meetings, turnaround time from case submission to genomic testing, and from receipt of genomic testing results to therapeutic recommendations, any other reported turnaround time, guidelines used to support recommendations, compliance with MTB recommendations, genomic profiling success rate.

*Patients and tumor characteristics:* patient characteristics (age, sex, ECOG status, median previous lines of treatment, if previously treated or not), cancer type and characteristics (e.g., stage, metastatic or non-metastatic), technology used for testing (e.g., NGS, IHC, CGH), NGS application (e.g., CGP, WES, t-NGS), total number of actionable alterations identified, number of patients in whom at least one actionable mutation is detected, number of patients who received an actionable therapy, number of patients who declined the suggested therapy, total number of patients treated.

*Clinical outcomes:* OS, PFS, median OS, median PFS, and PFS ratio ≥1.3 [22], overall mortality, treatment response following MTB-recommended therapy including objective response rate (ORR) and disease control rate (DCR) and any other relevant result of the study.

Moreover, for each study, we extracted the starting point for measuring survival time (e.g., from randomization, from referral to MTB, from treatment initiation) and the response assessment criteria (e.g., RECIST) used to evaluate therapeutic response and disease progression.

Furthermore, in order to control for potential selective reporting bias, we contacted the Principal Investigators of the RCTs included to request additional data when protocol-planned outcomes were not reported in the publication or when clarifications were needed for accurate data extraction. We also contacted Principal Investigators of RCTs with expected results by 2025 as reported on Clinical Trials.gov.

### Risk of bias assessment

Three independent reviewers (L.R., T.O., N.L.) assessed the risk of bias for each included study. For RCTs, we used the Cochrane Risk of Bias 2 (RoB 2) tool [23]. For non-randomized studies of interventions, both with and without a comparator, we applied the Risk Of Bias In Non-randomized Studies of Interventions (ROBINS-I) tool [24], using a modified version that excluded the domain related to bias due to confounding in studies without a comparator.

### Data synthesis and statistical methods

We synthesized data from all studies meeting the inclusion criteria through both descriptive and quantitative methods. The synthesis included a narrative summary of the evidence and, where applicable, a meta-analysis of clinical outcomes. Due to important methodological differences across study designs, we performed meta-analyses stratified by study design (RCTs, non-randomized clinical trials, prospective observational studies, and retrospective observational studies) and did not pool estimates across different study designs. One RCT [25] did not provide data usable for the meta-analysis and results are reported narratively.

Table B in S1 Appendix provides an overview of outcomes, effect measures, eligible study designs, and planned sensitivity analyses. For outcome definition, we defined ORR as the sum of patients achieving complete remission (CR) and partial remission (PR), and the DCR as the sum of patients with CR, PR, mixed remission (MR), and stable disease (SD). The PFS ratio was calculated as the ratio between PFS following MTB-recommended therapy and PFS during the previous line of therapy (prior to MTB assessment).

We conducted meta-analyses to evaluate quantitative outcomes, using hazard ratios (HRs) as the effect size for OS and PFS. When available, adjusted effect estimates were preferentially extracted to account for potential confounding; in the absence of adjusted estimates, unadjusted estimates were used. When HRs were not reported, we estimated them from published Kaplan–Meier survival curves and summary statistics using the method proposed by Tierney and colleagues [26]. WebPlotDigitizer tool (https://automeris.io/) was employed to extract information from survival data (Table C in S1 Appendix). Overall, 20 HRs were reconstructed from Kaplan–Meier curves. We calculated ORR and DCR if they were not directly reported in the original studies. When a comparator group was available, we performed a meta-analysis of RR for these outcomes, applying a continuity correction of 0.5 only in the presence of zero cells; studies with zero events in both arms were excluded. For the analysis of the PFS ratio, we calculated pooled proportions to quantify the proportion of patients achieving a PFS ratio ≥1.3, using the Freeman–Tukey double arcsine transformation to stabilize variance across studies [27].

We presented results separately according to the different effect measures. We calculated pooled estimates using the inverse variance-weighted method under a random-effects model to account for anticipated between-study heterogeneity due to variations in study design and patient population [28]. We estimated between-study variance using the restricted maximum-likelihood (REML) method [29], selected for its favorable statistical properties compared with traditional estimators, particularly in the presence of heterogeneity or when the number of studies is small [30]. For each random-effects model, we additionally calculated 95% prediction intervals (PIs) to estimate the expected range of true effects in future comparable studies.

We assessed heterogeneity using Cochrane’s *Q* test, and quantified it through the *I*^2^ statistic, with values of <50% interpreted as low; between 50% and 75% as moderate; and >75% as high heterogeneity, respectively [31]. We considered results statistically significant at a *p*-value <0.05. Results were visualized using forest plots, which included point estimates, 95% confidence intervals (CIs), and study descriptors (first author and year of publication). To evaluate robustness, we performed a leave-one-out sensitivity analysis, assessing the influence of each individual study on pooled estimates and heterogeneity.

For the publication bias assessment, we used funnel plots and Egger’s regression test, and we computed the test for excess significance [32]. Additionally, we conducted meta-regression analyses to explore potential sources of between-study heterogeneity, using a mixed-effects model. These analyses were performed across all included studies, with study design included as a covariate. To avoid case-wise exclusions and maximize data availability, we restricted analyses to study-level variables that were consistently reported across studies. Additional study characteristics (year of publication, inclusion of single versus multiple tumor types, and type of comparator) were examined individually in separate meta-regression models. We quantified the proportion of heterogeneity explained by each model using the *R*^2^ statistic. We estimated odds ratios (ORs) with corresponding 95% CIs for each covariate to assess their potential influence on the pooled effect sizes. In addition, we performed a sensitivity analysis in non-randomized studies for OS, PFS, and PFS ratio ≥1.3, restricting the analysis to studies in which survival time was measured from treatment initiation, in order to reduce immortal time bias, which can lead to overestimation of treatment effects. We also conducted additional analyses restricted to studies that used RECIST-based criteria to assess ORR and DCR, to ensure consistency in response evaluation across studies. We also performed sensitivity analyses excluding HRs reconstructed from Kaplan–Meier curves.

Lastly, we conducted sensitivity analyses by excluding studies rated as having a serious or critical risk of bias, in order to assess the robustness of the pooled estimates.

For all statistical analyses, we used R software version 4.4.0 (2024-04-24) for Windows and utilized the Meta package for conducting the meta-analyses.

## Results

**Fig 1:**
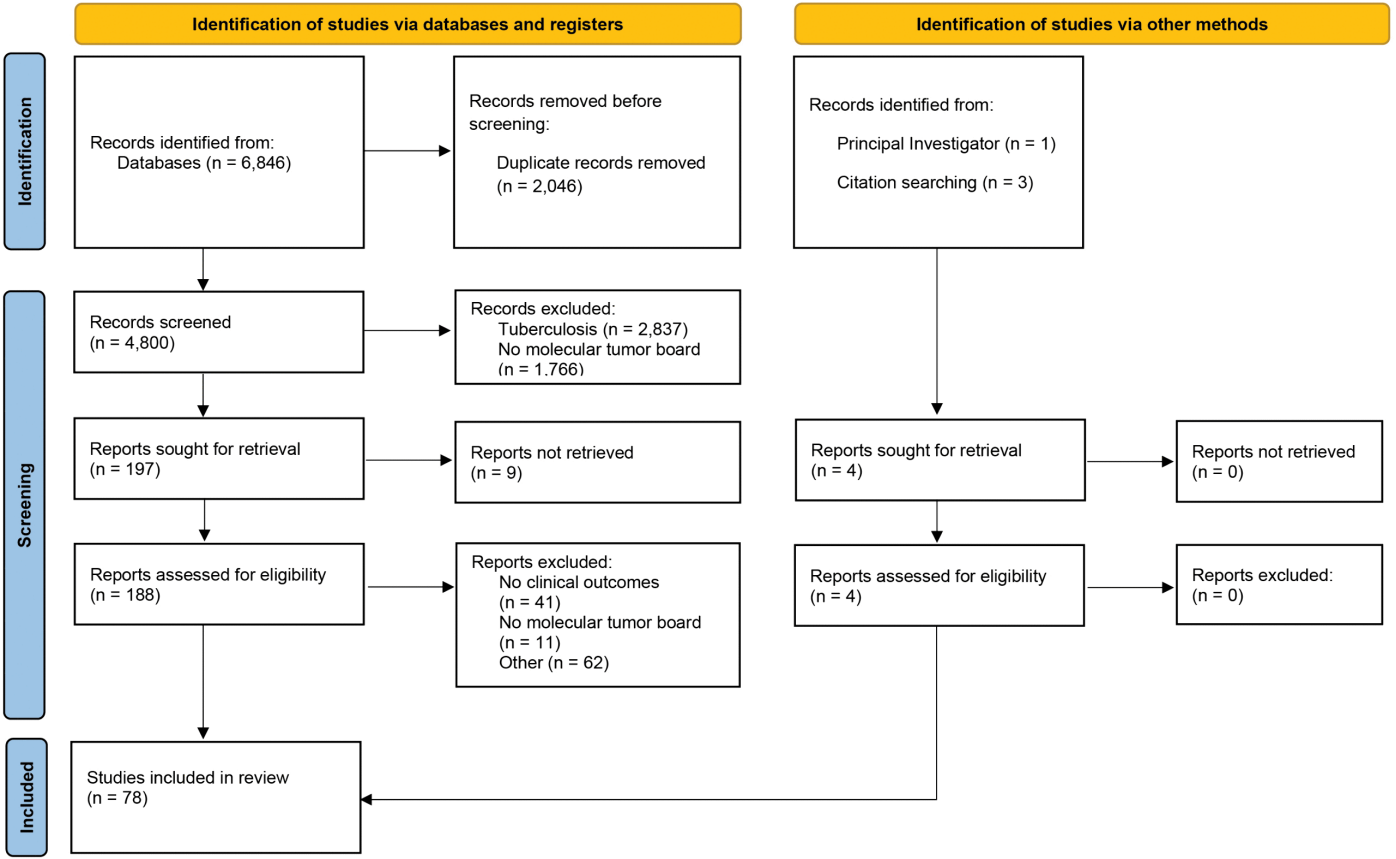
PRISMA flowchart.

The literature search retrieved 6,942 records. After deduplication, we screened 4,896 documents for title and abstract and 188 for full text. A total of 78 [14,25,33–108] records met the inclusion criteria, of which 74 were identified through scientific database searches, three through citation screening [33,35,37], and one provided directly by the Principal Investigator before publication [108] (Fig 1). Additionally, no missing outcomes were identified other than OS from the SHIVA trial, for which we obtained data directly from the Principal Investigator[14]. Furthermore, no additional unpublished trials were found on ClinicalTrials.gov, except for the ongoing MULTISARC trial [109]. The Principal Investigator informed us that they do not yet have data to share, as the analyses are still ongoing.

Table 1 and Table D in S1 Appendix show the characteristics of the studies included. We considered primary data on patients’ outcomes for a total of 9,195 patients discussed by the MTBs, of which 4,569 received the MTB-recommended therapy and 4,626 did not and were considered as comparators.

**Table 1:** Characteristics of the 78 studies included in the systematic review, by study design.

Study, year	Country	Sex	Age, median (range)^a^	Cancer details	Comparator details	N° patients in MTB arm (*N* = 4,569)	N° patients in comparator arm (*N* = 4,626)	Outcomes
**RCT (*N* = 7)**								
Le Tourneau (2015)	France	M:39%, F:61%	61 (54,96)	Metastatic cancers	Physician’s choice	100	97	OS, PFS, Median PFS, ORR
Belin (2017)	France	M:28%, F:72%	63 (24,88)	Metastatic cancers	Physician’s choice	25	70	PFS ratio ≥ 1.3, Median PFS, ORR
Schneider (2021)	USA	F:100%	50	Non-metastatic breast cancer	Physician’s choice	65	70	OS
Andrè (2022)	France	M:2%, F:98%	53.0 (26.0, 76.0)	Metastatic breast cancer	Physician’s choice	157	81	OS, PFS, Median OS, Median PFS, ORR, DCR, CR, PR, SD, PD
Krämer (2024)	Multinational	M:51%, F:49%	61.0 (53.0, 70.0)	Metastatic CUP	Platinum-Based Chemotherapy	326	110	OS, PFS, Median OS, Median PFS, ORR, DCR, CR, PR, SD, PD
Marchetti (2025)	Italy	M:48%, F:52%	61 (22,85)	Metastatic cancers	SoC	200	200	OS, PFS, Median OS, Median PFS, ORR, DCR, CR, PR, SD, PD
Sarno (2025)	Spain	M:54%, F:46%	62.4 (38,84)	Metastatic pancreatic cancer	Physician’s choice	4	0	Median OS
**Non-Randomized Clinical Trial (N = 16)**								
Massard (2017)	France	M:51%, F:49%	57 (19,86)	Metastatic or advanced cancers	–	194	–	PFS ratio ≥ 1.3, ORR
Sicklick (2019)	USA	M:34%, F:66%	62 (59,65)	Metastatic cancers	–	83	–	PFS ratio ≥ 1.3, Median OS, Median PFS
Trédan (2019)	France	M:37%, F:63%	53 (44,62)	Metastatic or recurrent cancers	–	163	–	ORR, DCR, CR, PR, SD, PD
Varnier (2019)	France	F:100%	60 (21,84)	Metastatic or advanced gynecological cancers	Non-target therapy group	39	200	Median OS, Median PFS, ORR, DCR, CB, PR, SD
Reda (2020)	France	M:40%, F:60%	65 (24,94)	Metastatic or advanced cancers	Non-target therapy group	79	263	OS, PFS ratio ≥ 1.3, Median PFS, ORR, DCR, PR, SD, PD
Bertucci (2021)	France	M:25%, F:75%	59 (20,84)	Metastatic or advanced cancers	Physician’s choice	94	160	PFS, PFS ratio ≥ 1.3, Median OS, Median PFS, ORR, DCR, CR, PR, SD, PD
Hlevjak (2021)	Germany	F:100%	53 (27,79)	Metastatic breast cancer	Non-target therapy group	64	63	OS, PFS ratio ≥ 1.3
Billon (2022)	France	M:91%, F:9%	Mean 65.55 (28,83)	Metastatic or advanced cancers	Physician’s choice	12	23	OS, PFS, PFS ratio ≥ 1.3, ORR, DCR, CR, PR, SD, PD
Louie (2022)	USA	M:47%, F:53%	56 (31,74)	Metastatic or advanced colorectal cancer	Non-target therapy group	34	17	OS, PFS, Median OS, Median PFS, DCR, CB
Miller (2022)	USA	M:41%, F:59%,	63 (18,85)	Metastatic or not cancers	–	93	–	PFS ratio ≥ 1.3, Median OS, Median PFS
Martin-Romano (2022)	France	M:49%, F:51%	59 (23,90)	Metastatic cancers	–	136	–	Median OS, Median PFS, ORR, DCR, PR, SD, PD
Debien (2023)	France	M:38%, F:62%	Mean 51	Metastatic or not cancers	Non-target therapy group	49	50	OS, PFS ratio ≥ 1.3, Median OS, Median PFS
Ladekarl (2023)	Denmark	M:43%, F:57%	64 (31,81)	Metastatic or advanced cancers	–	15	–	ORR, CR, PR
Blanc-Durand (2024)	France	F:100%	66.9 (60.6, 73.3)	Metastatic ovarian cancer	–	9	–	Median PFS, ORR, DCR, PR, PD
Sholler (2024)	USA	M:57%, F:43%	11 (1,23)	Refractory pediatric CNS or rare tumors	–	124	–	Median PFS, ORR, DCR, CR, PR, SD, PD
Vitale (2024)	Italy	M:52%, F:48%	66 (31,89)	Metastatic NSCLC	–	151	–	Median OS, Median PFS
**Observational Prospective (N = 20)**								
Dalton (2017)	USA	M:35%, F:65%	56 (17,89)	Advanced cancers	Non-target therapy group	24	54	PFS, Median PFS
Powell (2018)	USA	M:47%, F:53%	64.8 (33, 86.5)	Advanced cancers	–	39	–	Median OS, Median PFS, ORR, DCR, SD, PD
Rodriguez-Rodriguez (2018)	USA	F:100%	61 (22,80)	Metastatic or refractory gynecologic cancers	–	25	–	DCR, Response (CR, PR, SD), CB
Horak (2021)	Germany	M:51%, F: 49%	45 (16,82)	Advanced cancers	–	362	–	PFS ratio ≥ 1.3, Median OS, ORR, DCR
Huang (2021)	USA	M:40%, F:60%	Mean 66	NSCLC	Non-target therapy group	77	879	OS
Kikuchi (2021)	Japan	M: 41%, F:59%	61 (0, 79)	Metastatic or advanced cancers	–	21	–	ORR, DCR
Sultova (2021)	Germany	F:100%	52 (30,82)	Metastatic breast cancer	–	16	–	PFS ratio ≥ 1.3
Charo (2022)	USA	F:100%	50 (20,80)	Gynecologic cancers	Physician’s choice	16	52	OS, PFS, Median OS, Median PFS
Malani (2022)	Finland	M:48%, F:52%	63 (17,81)	AML	–	37	–	Median OS, ORR
Scheiter (2022)	Germany	M:62%, F:38%	Mean (sd) 56.19 (14.04)	Metastatic or advanced cancers	Physician’s choice	38	47	OS, PFS, PFS ratio ≥ 1.3, Median OS, Median PFS, ORR, DCR, CB, PR, MR, SD, PD
Mosteiro (2023)	Spain	M:69%; F:31%	64 (35,88)	NSCLC	Without an actionable driver	28	28	OS, Median OS
Pinet (2023)	France	M:50%, F:50%	64 (19,84)	Metastatic cancers	Non-target therapy group	13	69	OS, Median OS
Renovanz (2023)	Germany	M:55%, F:45%	53 (18,89)	Neurological rare tumors	–	86	–	PFS ratio ≥ 1.3, Median OS, Median PFS, DCR
Boer (2024)	Netherlands	M:71%, F:29%	13	Pediatric blood cancers	–	24	–	ORR, CR
Kato (2024)	USA	M:39%, F:61%	63 (21,95)	CUP	Non-target therapy group	37	25	OS, PFS, Median OS, Median PFS, DCR, CB
Kim (2024)	Korea	M:51%, F:49%	58 (24,88)	Metastatic or advanced cancers	–	89	–	Median OS, ORR, DCR, CR, PR, SD, PD
Lau (2024)	Australia, New Zealand	M:55%, F:45%	0.9 (0.1, 46)	Metastatic or advanced cancers	Non-target therapy group or SoC	99	75	OS, PFS, PFS ratio ≥ 1.3, ORR, DCR, CR, PR, SD, PD
Passiglia (2024)	Europe	M:55%, F:45%	70 (45, 77.5)	Metastatic NSCLC	Targetable alterations without matched targeted therapy	76	36	OS, PFS, Median OS, Median PFS, ORR
Mapendano (2025)	Denmark	M:43%, F:57%	65 (18,81)	Metastatic or advanced cancers	Non-target therapy group	30	53	OS, Median OS, ORR, DCR, CB, CR, PR, SD
Tsibulak (2025)	Germany	F: 100%	58 (18,87)	Gynecological cancers	–	9	–	PFS ratio ≥ 1.3, Median PFS, PD
**Observational Retrospective (N = 35)**								
Schwaederle (2014)	USA	M:21%, F:79%	56 (29,75)	Advanced cancers	–	12	–	ORR, DCR, PR, SD, PD
Parker (2015)	USA	F:100%	Mean 59 (55.71, 62.29)	Breast cancer	–	17	–	PFS ratio ≥ 1.3,
Kaderbhai (2016)	France	M:48%, F:52%	62.7 (20,79)	Lung cancer	–	9	–	Median PFS, ORR, PR
Burkard (2017)	USA	F:39%, M:61%	Mean 57 (33,88)	Advanced cancers	–	9	–	ORR, DCR, CB
Hoefflin (2018)	Germany	M:58%, F:42%	58 (1,85)	Metastatic or advanced cancers	Absence of treatment recommendations by the MTB	33	72	OS, PFS ratio ≥ 1.3, Median OS, ORR, DCR, PR, SD
Trivedi (2019)	USA	M:31%, F:69%	64 (37,82)	Advanced cancers	–	12	–	ORR, DCR, SD, PD
Bitzer (2020)	Germany	N.R.	N.R.	Gynecologic cancers	–	25	–	Median OS, Median PFS, ORR, DCR, PR, SD, PD
Kato (2020)	USA	M:41%, F:59%	61 (3,92)	Advanced cancers	Physician’s choice	86	164	OS, PFS
Koopman (2020)	Netherlands	M:34%, F: 53%, NR: 13%	68 (36,89)	Advanced cancers	–	25	–	Median OS, Median PFS, ORR, DCR, SD, PD
Angel (2021)	Argentina	M:50%, F:50%	57.8 (52, 68.7)	Advanced cancers	–	16	–	Median PFS, ORR, DCR, PR, SD, PD
Gambardella (2021)	Spain	M:34%, F:66%	Mean 59 (25,81)	Advanced cancers	SoC	32	66	PFS, PFS ratio ≥ 1.3, Median PFS, PD
Hoefflin (2021)	Germany	M:53%, F:47%	Mean 54 (1,88)	Metastatic or advanced cancers	Absence of treatment recommendations by the MTB	76	167	Median OS, ORR, DCR, CR, PR, SD
Niogret (2021)	France	M: 63%, F:37%	64 (20,87)	Lung cancer	Non-target therapy group	65	143	OS, PFS, Median OS, Median PFS, ORR, DCR, CR, PR, SD, PD
Ida (2022)	Japan	M:52%, F:48%	57 (3,86)	Colorectal, lung, soft tissue sarcoma, others	Without actionable driver	51	222	OS, Median OS
Slootbeek (2022)	Netherlands	M:100%	Mean 69.0 (62.6, 74.7)	Prostate cancer	Non-target therapy group	63	38	OS, Median OS, PFS, ORR, DCR, CR, PR, SD, PD
Tarawneh (2022)	Germany	M:50%, F:50%	Mean 57,5	Metastatic or advanced cancers	Non-target therapy group	30	17	PFS, Median PFS, DCR, CB
Berclaz (2023)	Germany	F:52%, M:48%	49 (0, 80)	Metastatic or advanced sarcomas	–	10	–	PFS ratio ≥ 1.3
Blobner (2023)	Germany	M:60%, F:40%	51 (8,77)	Advanced neurological cancers	–	12	–	PFS ratio ≥ 1.3, Median PFS, ORR, DCR, SD, PD
Dorman (2023)	Germany	M:37%, F:63%	62 (29,83)	Metastatic or advanced pancreatic cancer	SoC	88	90	OS, Median OS
El Helali (2023)	Hong Kong	M:53%, F:47%	Mean 60 (11,95)	Metastatic or advanced cancers	Not implemented MTB recommendation	77	15	OS, Median OS, ORR, DCR, CR, PR, SD, PD
Fukada (2023)	Japan	M:46%, F:54%	58 (12,85)	Metastatic or advanced cancers	Absence of treatment recommendations by the MTB	45	668	OS, Median OS, ORR, DCR, PR, SD, PD
Ghanem (2023)	USA	M:56%, F:44%	Mean (sd) 43.0 (17.3)	Advanced cancers	Non-target therapy group	18	26	OS, PFS, Median OS, Median PFS, ORR, DCR, CR, PR, SD, PD
Giacomini (2023)	Italy	N.R.	N.R.	Advanced cancers	–	22	–	PFS ratio ≥ 1.3
Limousin (2023)	France	M:70%, F:30%	57.0 (43.5, 70.2)	Metastatic or advanced hepatic cancers	–	9	–	Median OS, DCR, SD
Repetto (2023)	Italy	M:40%, F:60%	Mean 54	Metastatic or advanced cancers	SoC	76	70	OS, PFS, PFS ratio ≥ 1.3, Median OS, Median PFS, ORR, DCR, CR, PR, SD, PD
Shaya (2023)	USA	M:39%, F:61%	67 (47,84)	Metastatic pancreatic cancer	–	18	–	Median OS, Median PFS, DCR, CB
Weiss (2023)	Germany	M:57%, F:43%	60 (23,85)	CUP	–	4	–	Median PFS
Zhang (2023)	Germany	M:47%, F:53%	62 (24,81)	Biliary tract cancers	Absence of treatment recommendations by the MTB	14	56	OS, PFS ratio ≥ 1.3, Median OS, Median PFS, ORR, DCR, PR, SD, PD
Dang (2024)	Luxembourg	M:38%, F:62%	53.38	Metastatic or advanced cancers	Non-target therapy group	8	26	PFS ratio ≥ 1.3, Median PFS, ORR, DCR, PR, SD, PD
De Jager (2024)	Netherlands	M:42%, F:58%	69 (59,76)	Lung cancer and melanoma	–	57	–	Median OS, Median PFS, ORR, DCR, CR, PR, SD, PD
Dreikhausen (2024)	Germany	M:63%, F:37%	61 (24,90)	Advanced or metastatic gastrointestinal cancer or CUP	–	13	–	Median PFS
Gremke (2024)	Germany	F:100%	59 (26,84)	Metastatic or advanced cancers	Non-target therapy group	23	29	PFS, Median PFS
Louie (2024)	USA	M:46%, F:54%	60 (3,92)	Rare/ultra-rare metastatic or advanced cancers	–	112	–	Median OS, Median PFS, ORR, DCR, CR, PR, SD, PD
Perez (2024)	Spain	M:88%, F:12%	67.9 (IQR 61.2, 73.2)	Metastatic urothelial cancer	Without actionable driver	7	18	Median OS, Median PFS, ORR, DCR, PR, SD, PD
Boscolo Bielo (2025)	Italy	F:100%	53 (IQR 47, 61)	Metastatic breast cancer	SoC	33	17	OS, PFS, Median OS, Median PFS, DCR

^a^Unless otherwise specified. When both median age and mean age were reported, only median was considered; –, studies that did not have a comparator.

M, Male; F, Female; PFS, progression-free survival; ORR, Overall Response Rate; OS, Overall Survival; DCR, Disease Control Rate; CR, Complete Response; PR, Partial Response; SD, Stable Disease; PD, Progressive Disease; CUP, Carcinoma of Unknown Primary; SoC, Standard of Care; CB, Clinical Benefit; CNS, Central Nervous System; NSCLC, Non-small cell lung carcinoma; AML, Acute Myeloid Leukemia; MR, Mixed Response.

Overall, 53.8% of the studies included had a comparator group, most frequently involved patients treated with non-targeted therapies (40.5%) or receiving treatment at physician’s discretion (23.8%). The remaining 46.2% were single-arm studies. Study design varied across the publications included: the majority were retrospective observational studies (44.9%), followed by prospective observational studies (25.6%) and non-randomized clinical trials (20.5%). RCTs were the minority (seven studies, 9.0%).

The majority of studies were conducted after 2020 (75.6%) and in Europe (65.4%), namely in Germany (21.8%) and France (20.5%) (Table D in S1 Appendix). Most studies included both male and female patients (83.3%), while a smaller proportion (12.8%) focused exclusively on female patients due to the inclusion of only gynecologic cancers. Regarding patient demographics, most studies focused on adult populations (78.2%). The majority of studies enrolled patients who were either a mix of treatment-naive and previously treated (43.6%) or solely previously treated (50.0%). In 21.8% of the studies, the entire study population presented with metastatic disease at the time of MTB evaluation, with only one study (1.3%) included exclusively non-metastatic patients. The percentage of patients with ECOG of 0–1 was reported in only 29.5% of studies and RECIST 1.1 was the most frequently used response assessment criteria (67.6%), while 28.2% did not specify the criteria used. A wide variability in clinical outcomes was observed across the included studies. Survival time was measured from randomization in all seven RCTs (100%), and from treatment initiation in 56.3% of the non-randomized studies, while 24.4% did not clearly define the starting point. The majority reported information on ORR and DCR (59.0%), as well as survival outcomes, such as median PFS (57.7%) and median OS (55.1%) (Table D in S1 Appendix).

The main characteristics of the MTBs reported in the included studies are summarized in Table 2. In most of the cases, the access to MTBs was through treating physicians (16.7%) or institutional referrals (28.2%), although more than half of the studies (51.3%) did not report how cases were referred. Board composition varied, with most MTBs consisting of 1–5 (32.1%) or 6–10 (41.0%) members, and meetings held weekly (28.2%) or biweekly (11.5%). Oncologists (80.8%), pathologists (66.7%), geneticists (53.8%), and bioinformaticians (46.2%) were the most involved specialties in MTBs. Notably, 19.2% of the studies did not specify the specialties represented in the meetings.

**Table 2:** Characteristics of the MTBs reported in the included studies.

Variable		*N* (%)
**Methods of access to the MTB**	Referred by the treating physician/oncologist	13 (16.7%)
	From the hospital or same institution	22 (28.2%)
	Cases submitted by Steering Committees/external referrals	3 (3.8%)
	N.R.	40 (51.3%)
**Number of members of the MTB**	1–5	25 (32.1%)
	6–10	32 (41.0%)
	11–15	4 (5.1%)
	16–20	2 (2.6%)
	N.R.	15 (19.2%)
**Professional figures involved** ^a^	Oncologist^b^	63 (80.8%)
	Pathologist	52 (66.7%)
	Geneticists	42 (53.8%)
	Bioinformaticians	36 (46.2%)
	Biologist	29 (37.2%)
	Researchers or scientists	23 (29.5%)
	Radiologists	18 (23.1%)
	Physicians	15 (19.2%)
	Surgeons	12 (15.4%)
	Clinical trial coordinators or staff	12 (15.4%)
	Pharmacists	9 (11.5%)
	No panel members reported	15 (19.2%)
**Frequency of board meetings**	Once a week	22 (28.2%)
	Once every two weeks	9 (11.5%)
	Three times a month	5 (6.4%)
	Monthly	3 (3.8%)
	Twice per week	2 (2.6%)
	N.R.	37 (47.4%)
**Guidelines used for therapy recommendations**	ESMO-ESCAT	31 (39.7%)
	ASCO guidelines	2 (2.6%)
	Other	8 (10.3%)
	N.R.	37 (47.4%)
**Technology used** ^a^	NGS	78 (100.0%)
	IHC	16 (20.5%)
	CGH	7 (9.0%)
	FISH	3 (3.8%)
**NGS application** ^a^	t-NGS	31 (39.7%)
	CGP	23 (29.5%)
	WES	17 (21.8%)
	RNA-seq	16 (20.5%)
	WGS	5 (6.4%)
	mRNAseq	2 (2.6%)
	lcWGS	2 (2.6%)
	RNA Fusion Detection	1 (1.3%)
	l-NGS	1 (1.3%)
**Sample for analysis** ^a^	Biopsy sample	78 (100.0%)
	Liquid biopsy (on ctDNA)	16 (20.5%)
**Type of cancers discussed by the MTB**	Solid tumors	68 (87.2%)
	Hematologic malignancies	2 (2.6%)
	Both	8 (10.3%)
**Number of cancers discussed in the MTBs**	One^c^	25 (32.1%)
	2–5	14 (17.9%)
	6–10	10 (12.8%)
	11–15	12 (15.4%)
	16–20	6 (7.7%)
	>20	11 (14.1%)
**Most frequent cancers discussed in the MTBs** ^a^	Breast	40 (51.3%)
	Lung (including NSCLC)	36 (46.2%)
	Ovarian	34 (43.6%)
	Colorectal	26 (33.3%)
	Pancreas	25 (32.1%)

^a^The total percentages are more than 100% since more than one option could be included in each study.

^b^In 7 studies an oncologist was not explicitly reported as included in the panel; 3 of these studies reported the presence of a ‘physicians or clinicians’ in an unspecific manner; in the remaining 4 studies, it was reported the inclusion in the MTB professionals like ‘organ specialists’, ‘Physicians with molecular cancer expertise’, ‘experts on precision oncology’, and ‘Physicians specializing in cancer pharmacotherapy’.

^c^Three of the single cancer focus MTBs discussed only Carcinoma of Unknown Primary.

N.R., Not Reported; NGS, Next Generation Sequencing; IHC, Immunohistochemistry; CGH, Comparative Genomic Hybridization; FISH, Fluorescence In Situ Hybridization; t-NGS, Targeted Next Generation Sequencing; CGP, Comprehensive Genomic Profiling; WES, Whole Exome Sequencing; RNA-seq, RNA Sequencing; WGS, Whole Genome Sequencing; mRNAseq, Messenger RNA Sequencing; lcWGS, Low-Coverage Whole Genome Sequencing; l-NGS, large Next Generation Sequencing; ctDNA, circulating tumor DNA; NSCLC, non-small cell lung carcinoma; ECOG, Eastern Cooperative Oncology Group performance status.

Most cancers discussed were solid tumors (87.2%). Thirty-two percent of MTBs discussed single cancer, with three of those discussing only carcinoma of unknown primary (CUP). The most frequently discussed cancers were breast (51.3%), lung (46.2%), ovarian (43.6%), colorectal (33.3%), and pancreatic (32.1%).

The ESMO Scale for Clinical Actionability of molecular Targets (ESCAT) [110] framework was the most frequently cited guideline for therapy recommendations (39.7%). All studies utilized NGS, with targeted NGS (t-NGS, 39.7%) and comprehensive genomic profiling (CGP, 29.5%) being the most common applications. Tissue biopsy was used in all cases (100%), while ctDNA-based liquid biopsy was performed in 20.5% of studies (Table 2). Further detailed descriptions of each included MTB are presented in Table E in S1 Appendix.

### Clinical outcomes

Of the 78 studies identified, 52 were included in the meta-analysis, with results presented below, while non-meta-analyzed outcomes are detailed in Table F in S1 Appendix.

#### Meta-analysis results.

Table 3 shows the results of the 52 studies included in the meta-analysis, stratified by clinical outcome and study design.

**Table 3:** Meta-analysis results stratified by outcome and study design.

Outcome	Study Design	Studies (*N*)	N° patients in MTB/comp	Pooled outcome measure (95% CI)	*I* ^2^	Prediction Interval
Overall Survival (OS)	RCT	5	848/558	HR = 0.87 (0.76, 1.01)	0.0%	0.71, 1.07
	Non-Randomized Clinical Trial	5	195/353	HR = 0.83 (0.65, 1.05)	0.0%	0.58, 1.17
	Observational Prospective	9	457/1330	HR = 0.56 (0.36, 0.86)	74.1%	0.13, 2.32
	Observational Retrospective	12	650/1558	HR = 0.62 (0.56, 0.69)	10.8%	0.55, 0.70
Progression-free survival (PFS)	RCT	4	782/486	HR = 0.73 (0.64, 0.84)	0.0%	0.59, 0.91
	Non-Randomized Clinical Trial	3	139/174	HR = 0.70 (0.51, 0.96)	3.8%	0.28, 1.77
	Observational Prospective	6	330/280	HR = 0.73 (0.60, 0.88)	0.0%	0.57, 0.94
	Observational Retrospective	8	353/530	HR = 0.63 (0.54, 0.74)	0.0%	0.53, 0.76
Progression-free survival ratio ≥ 1.3 (PFS ratio ≥ 1.3)	RCT	1	68	Prop = 0.368 (0.246, 0.486)	–	–
	Non-Randomized Clinical Trial	8	560	Prop = 0.331 (0.272, 0.393)	47.0%	0.184, 0.496
	Observational Prospective	6	446	Prop = 0.381 (0.335, 0.429)	22.6%	0.321, 0.444
	Observational Retrospective	9	173	Prop = 0.430 (0.353, 0.509)	0.0%	0.340, 0.523
Objective Response Rate (ORR)	RCT	4	781/480	RR = 1.75 (1.24, 2.47)	0.0%	1.00, 3.05
	Non-Randomized Clinical Trial	2	101/163	RR = 1.19 (0.38, 3.72)	64.0%	NI^†^
	Observational Prospective	3	204/126	RR = 1.84 (0.39, 8.76)	79.7%	NI^†^
	Observational Retrospective	3	147/233	RR = 3.32(2.25, 4.89)	0.0%	1.42, 7.77
Disease Control Rate (DCR)	RCT	3	683/391	RR = 1.20 (1.03, 1.40)	19.9%	0.77, 1.88
	Non-Randomized Clinical Trial	3	133/180	RR = 1.62 (0.83, 3.20)	58.8%	0.14, 18.99
	Observational Prospective	3	162/111	RR = 1.26 (0.73, 2.18)	75.3%	0.15, 10.47
	Observational Retrospective	4	180/249	RR = 1.65 (1.02, 2.68)	74.7%	0.34, 7.94

† Extremely wide and not clinically interpretable.

HR, hazard ratio; Prop, proportion; RR, relative risk; CI, confidence interval.

**Fig 2:**
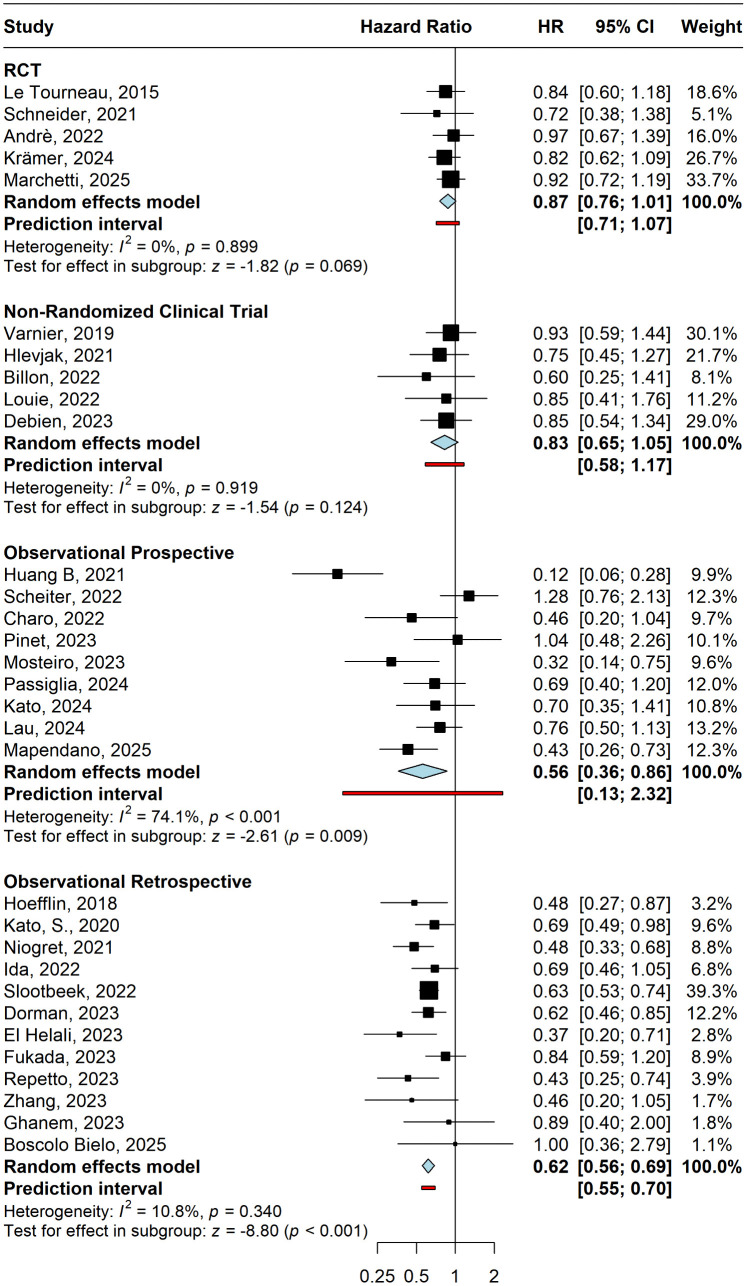
Meta-analysis of overall survival (OS) stratified by study design. HR, hazard ratio; CI, confidence interval. *P*-values were derived from Cochran’s *Q* test (for heterogeneity) and *Z*-tests (Wald-type tests) for pooled effects.

*Overall survival (OS):* The analysis within each study design showed a general trend toward improved OS with MTB-guided therapy. The analysis suggested a reduction in the risk of death associated with MTB-based treatment, with risk reductions ranging from 13% to 43%, depending on study design. In RCTs, the pooled estimate yielded a HR of 0.87 (95% CI [0.76, 1.01]; *p* = 0.069; *I*^2^ = 0.0%), and in non-randomized clinical trials the HR was 0.83 (95% CI [0.65, 1.05]; *p* = 0.124; *I*^2^ = 0.0%). Observational studies reported lower HRs for OS. In retrospective studies, the pooled HR was 0.62 (95% CI [0.56, 0.69]; *p* = 0.009; *I*^2^ = 10.8%), and in prospective studies, 0.56 (95% CI [0.36, 0.86]; *p* < 0.001), although with greater heterogeneity (*I*^2^ = 74.1%) (Fig 2).

Across RCTs, the median OS ranged from 9.1 to 22.1 months in the intervention arm. In particular, it was 22.1 versus 25.3 [35]; 14.7 versus 11.0 [36]; 9.5 versus 10.3 [14] and 9.11 versus 7.86 [108] months, respectively, in the intervention and comparator arm for each trial (Table F in S1 Appendix).

**Fig 3:**
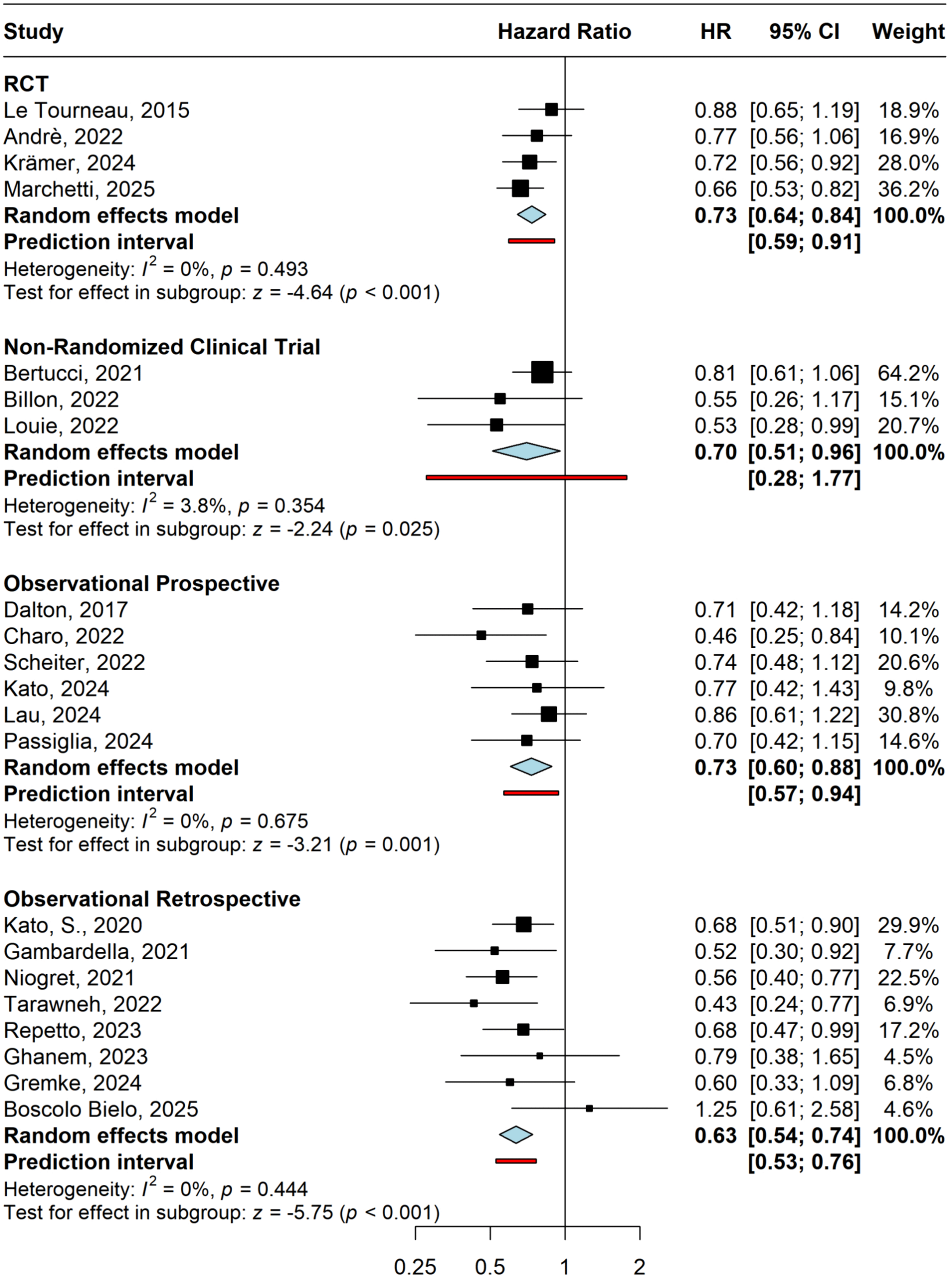
Meta-analysis of progression-free survival (PFS) stratified by study design. HR, hazard ratio; CI, confidence interval. *P*-values were derived from Cochran’s *Q* test (for heterogeneity) and *Z*-tests (Wald-type tests) for pooled effects.

*Progression Free Survival (PFS):* The meta-analysis demonstrated a significant reduction in the risk of disease progression following therapy recommended by the MTB, with risk reductions ranging from 27% to 37% depending on the study design. Heterogeneity was low across all study types. In RCTs, the pooled HR was 0.73 (95% CI [0.64, 0.84]; *p* < 0.001; *I*^2^ = 0.0%), and in non-randomized clinical trials, 0.70 (95% CI [0.51, 0.96]; *p* = 0.025; *I*^2^ = 3.8%). In prospective observational studies, the HR was 0.73 (95% CI [0.60, 0.88]; *p* = 0.001; *I*^2^ = 0.0%), and in retrospective studies, 0.63 (95% CI [0.54, 0.74]; *p* < 0.001; *I*^2^ = 0.0%) (Fig 3).

Across RCTs, the median PFS ranged from 2.3 to 6.0 months in the intervention arm. In detail, it was 2.3 versus 2.0 [14]; 5.5 versus 2.9 [35]; 6.0 versus 4.4 [36]; and 3.45 versus 2.80 [108] months, respectively, in the intervention and standard of care arm (Table F in S1 Appendix).

**Fig 4:**
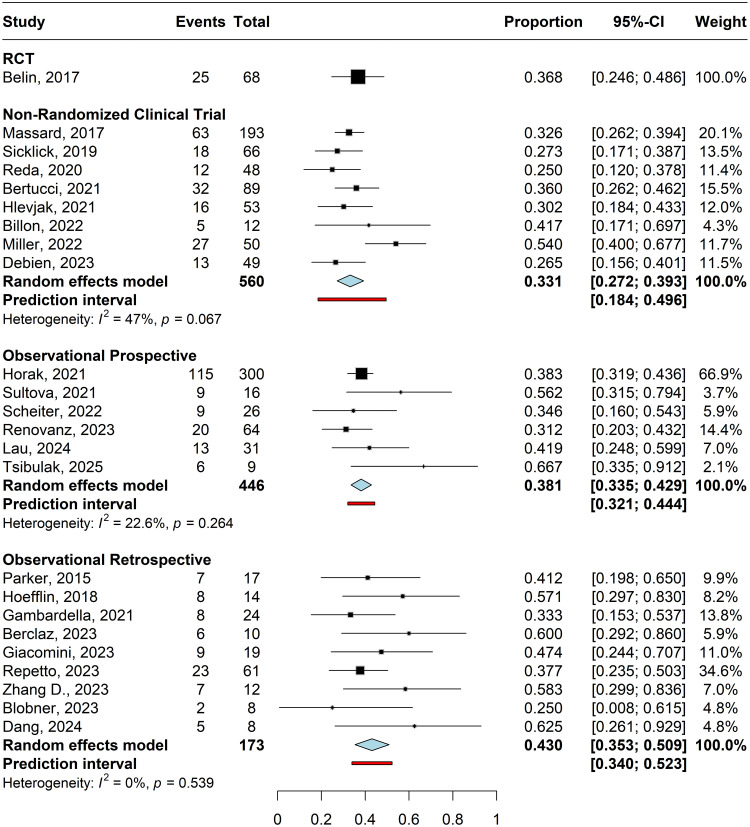
Meta-analysis of progression-free survival ratio (PFS ratio) ≥ 1.3 stratified by study design. CI, confidence interval. *P*-values were derived from Cochran’s *Q* test (for heterogeneity).

*Progression Free Survival ratio* ≥*1.3 (PFS ratio* ≥*1.3):* The proportion of patients achieving a PFS ratio ≥1.3 ranged from 33% to 43%, depending on study design. In RCTs, one study reported a proportion of 36.8% (95% CI [24.6, 48.6]). In non-randomized clinical trials, the pooled proportion was 33.1% (95% CI [27.2, 39.3]; *I*^2^ = 48.3%). In prospective observational studies, the proportion was 38.1% (95% CI [33.5, 42.9]; *I*^2^ = 22.7%), and in retrospective studies, 43.0% (95% CI [35.3, 50.9]; *I*^2^ = 0.0%) (Fig 4).

**Fig 5:**
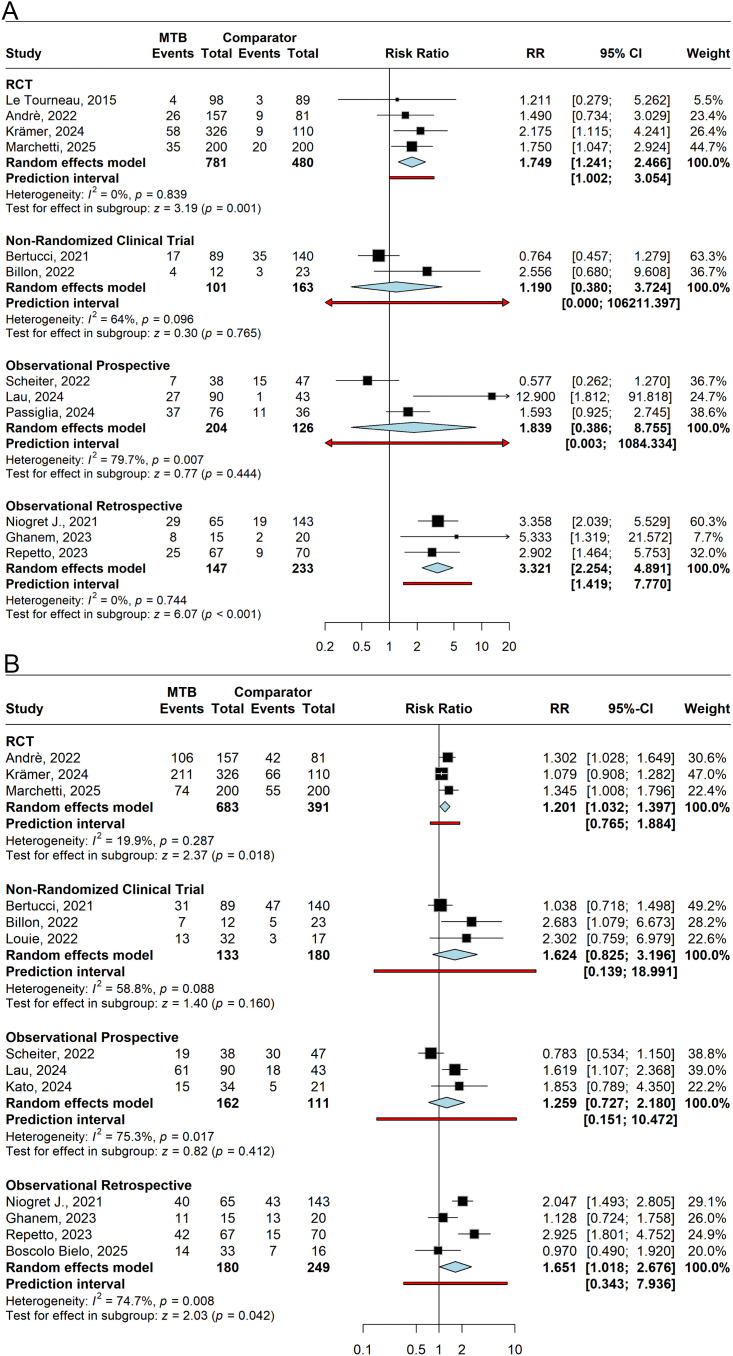
Meta-analysis of relative risk (RR) of Objective Response Rate (ORR) (A) and Disease Control Rate (B) stratified by study design. CI, confidence interval; RR, relative risk. *P*-values were derived from Cochran’s *Q* test (for heterogeneity) and *Z*-tests (Wald-type tests) for pooled effects.

*Objective response rate (ORR):* ORR was consistently higher with MTB-guided therapy, with RRs ranging from 1.19 to 3.32 depending on study design. The pooled RR in RCT was 1.75 (95% CI [1.24, 2.47]; *p* = 0.001; *I*^2^ = 0.0%) and in retrospective studies, 3.32 (95% CI [2.25, 4.89]; *p* = 0.765; *I*^2^ = 0.0%). Estimates from non-randomized clinical trials (RR 1.19, 95% CI [0.38, 3.72]; *p* = 0.444; *I*^2^ = 64.0%) and prospective observational studies (RR 1.84, 95% CI [0.39, 8.76]; *p* < 0.001; *I*^2^ = 79.7%) showed greater variability across studies (Fig 5A).

*Disease Control Rate (DCR):* DCR was also higher among patients treated according to MTB recommendations, with RR ranging from 1.20 to 1.65 depending on study design, although heterogeneity was generally high in non-randomized and observational studies. In RCT, the pooled RR was 1.20 (95% CI [1.03, 1.40]; *p* = 0.018; *I*^2^ = 19.9%). In non-randomized clinical trials, the RR was 1.62 (95% CI [0.83, 3.20]; *p* = 0.088; *I*^2^ = 58.8%). In prospective observational studies, the RR was 1.26 (95% CI [0.73, 2.18]; *p* = 0.017; *I*^2^ = 75.3%), and in retrospective studies, 1.65 (95% CI [1.02, 2.68]; *p* = 0.008; *I*^2^ = 74.7%) (Fig 5B).

*Sensitivity analysis:* The leave-one-out sensitivity analysis did not identify any influential studies for PFS and ORR. In contrast, four studies were found to be influential with respect to OS, PFS ratio, and DCR: Huang and colleagues (2021) [57] for OS, Miller and colleagues (2022) [46] for the PFS ratio, Bertucci and colleagues (2021) [42], and Scheiter and colleagues (2022) [62] for DCR. In each of these cases, the exclusion of the study resulted in a notable reduction in heterogeneity, as shown in Tables G–K in S1 Appendix, without affecting the overall effect estimate.

Meta-regression analyses showed that study design explained a substantial proportion of between-study heterogeneity for OS (*R*^2^ = 79.2%) and ORR (*R*^2^ = 86.3%). Other examined covariates were not significantly associated with outcomes. Residual heterogeneity was low for PFS and PFS ratio ≥1.3 (*I*^2^ approximately 0%–30%). In contrast, residual heterogeneity remained high for DCR across models (*I*^2^ approximately 60%−80%) (Table L in S1 Appendix).

As illustrated in Figs A–E in S2 Appendix and Table M in S1 Appendix, funnel plots and Egger’s tests did not indicate substantial asymmetry. Similarly, no clear indication of selective reporting was identified based on the test for excess significance (Table N in S1 Appendix).

Results were consistent in analyses restricted to non-randomized studies in which survival time was measured from treatment initiation, with no change in the direction of the pooled estimates for OS and PFS, nor in the proportion of PFS ratio ≥1.3 (Figs F–H in S2 Appendix). Similar consistency was observed in analyses restricted to studies applying RECIST 1.1 criteria for ORR and DCR (Fig I in S2 Appendix). Similarly, sensitivity analyses excluding HRs reconstructed from Kaplan–Meier curves confirmed the robustness of the findings, with no relevant changes in effect estimates (Figs J and K in S2 Appendix).

### Risk of bias assessment

Overall, the risk of bias for RCTs was judged to be low, except for one (14.3%) study that was rated as having “some concerns” (Fig L in S2 Appendix). In contrast, the overall risk of bias for non-randomized clinical trials, prospective observational studies, and retrospective observational studies was predominantly rated as “serious” in nine studies (56.3%), 18 studies (90.0%), and 27 studies (77.1%), respectively (Table O in S1 Appendix). Most of the remaining studies were rated as having moderate risk of bias, with the exception of four retrospective studies (11.4%) assessed as having a critical risk. A detailed breakdown of ROBINS-I domain-level judgments is available in Table P in S1 Appendix.

All results remained robust after excluding studies judged to have serious or critical risk of bias, with no changes in the direction of pooled effect estimates across outcomes and study designs, except for OS in prospective observational studies, which became non-significant (Figs M–P in S2 Appendix).

## Discussion

This systematic review and meta-analysis aimed to assess the clinical impact of MTB-recommended therapies on patients with cancer outcomes.

Based on 78 studies involving 9,195 patients, this analysis supports an association between MTB-based therapeutic decision-making and improved clinical outcomes when restricting the results to high-quality studies, PFS benefits ranged from 27% to 37%, and approximately one-third to 40% of patients achieved a PFS ratio ≥1.3. Comparable improvements were observed also for ORR and DCR.

Importantly, while prospective and retrospective observational studies showed a significant improvement in OS, the evidence from RCTs and non-randomized clinical trials remains uncertain.

This work represents the most extensive and comprehensive synthesis of evidence to date, providing information that MTB-recommended therapies are associated with a reduced risk of disease progression, as well as improved objective response and DCRs, but do not appear to clearly affect the risk of death.

The meta-analysis conducted by Gladstone and colleagues [13] included 34 studies involving 2,532 patients and reported significant HRs of 0.46 (0.24, 0.88) for OS across seven studies, and 0.65 (0.41, 1.03) for PFS in three studies. However, the authors did not stratify results by study design and did not include RCTs. In our review, we were able to include a larger number of studies providing comparative data, and we additionally conducted a meta-analysis for RR of ORR and DCR, likely due to our broader search strategy.

The results indicate clear differences on patients’ outcomes according to study design, with randomized evidence suggesting benefits primarily in terms of disease progression and treatment response, with a more limited effect on OS, whereas observational studies report favorable association between MTB-guided therapy and all the investigated outcomes.

The heterogeneity observed in our review reflects the fragmented and still non-standardized nature of MTBs. Significant efforts have been made in recent years to address these issues and to develop widely accepted guidelines for establishing and operating MTB [111,112]. Access to MTB-recommended therapies also differs across healthcare systems. In the United States, broader availability of clinical trials and off-label drugs may facilitate access [113], although this often depends on funding and insurance coverage [114]. In contrast, European MTBs may face more restricted access to certain drugs but tend to promote more equitable treatment allocation [115]. From a health system perspective, the implementation of MTBs raises important questions regarding sustainability and value. MTBs require substantial organizational resources, multidisciplinary expertise, and access to complex diagnostic technologies. Health systems therefore face a dilemma between the promise of more personalized care and the need to justify investment in MTBs against measurable patient benefit. In this context, generating stronger clinical evidence, together with robust evaluations of cost-effectiveness and implementation feasibility, is essential.

Several limitations of primary studies must be acknowledged. In non-randomized studies, comparator groups were often poorly defined: in nearly half of the included studies, patients not receiving MTB-recommended therapies were described in vague terms, often including individuals no longer eligible for treatment due to clinical deterioration or death. This likely introduced substantial bias and may have exaggerated the observed benefit of MTB-guided therapy. In other cases, comparators were treated according to physician discretion or local practice, adding further variability. In addition, non-experimental studies were particularly susceptible to confounding, as they often lacked adequate adjustment for key clinical factors such as disease severity, comorbidities, and prior therapies. Furthermore, many of these studies were at risk of immortal time bias, as survival time was sometimes measured from a point preceding treatment initiation, while in other cases the starting point for survival measurement was not clearly reported. Selection bias was also common, with patients referred to MTBs differing systematically from those not referred, either because of more complex disease or different performance status. These biases were often present even in studies rated as low risk in ROBINS-I, highlighting the limitations of considering non-randomized evidence in supporting claims in favor of MTB impact on health outcomes. Although sensitivity analyses excluding studies at serious or critical risk of bias did not materially alter the results, findings from observational studies should still be interpreted with appropriate caution. Information bias further limited retrospective studies that relied on medical records, with inconsistencies in reporting of whether MTB recommendations were implemented, and how outcomes were measured. Most non-randomized evidence was judged to be at serious or critical risk of bias, underscoring the importance of interpreting such evidence with caution. In this context, recent high-quality trials such as the ROME study [108] represent a meaningful advancement in the evaluation of MTB efficacy under controlled conditions. Our findings highlight a need for additional high-quality randomized evidence to more reliably define the clinical impact of MTBs. Notably, the largest effect estimates were observed in retrospective and observational studies, whereas RCTs showed more modest benefits, particularly for OS, likely reflecting methodological limitations of non-randomized studies. PIs further support this interpretation, as in several analyses they were wide and included the null value, suggesting that the effect in future studies may vary substantially across settings and may include no clinically relevant benefit.

Our meta-analysis has several methodological limitations. First, despite stratifying by study design and using random-effects models, residual heterogeneity likely remains due to substantial clinical and methodological variability across studies, including differences in patient populations, tumor types, sequencing technologies, comparator definitions, and treatment contexts. Although stratified and meta-regression analyses suggested that study design accounted for a relevant proportion of heterogeneity, other potentially important effect modifiers could not be fully explored because of incomplete or inconsistent reporting across studies. In addition, study-level meta-regression is inherently limited using aggregated data, which may introduce ecological bias and has limited statistical power, when the number of included studies is small.

Second, in some studies, HRs for OS and PFS were not directly reported and had to be estimated from Kaplan–Meier curves. While widely accepted, this introduces additional uncertainty, particularly when survival data were graphically imprecise or lacked complete at-risk information. Nevertheless, this approach allowed inclusion of studies with incomplete reporting, potentially mitigating publication bias. However, sensitivity analyses excluding HRs reconstructed from Kaplan–Meier curves confirmed the robustness of the findings. Moreover, some outcomes (e.g., ORR, DCR) had to be reconstructed from partially reported data, potentially affecting accuracy despite adherence to standardized definitions. Interpretation of the PFS ratio also warrants caution, as it may be influenced by the selection of prior therapy and by methodological biases inherent to observational settings, such as regression to the mean and informative censoring, thereby introducing additional uncertainty in pooled estimates.

To reduce the potential impact of incomplete or selective reporting, we contacted the Principal Investigators of included studies to request missing data or clarifications when protocol-specified outcomes were unavailable. We also contacted the Principal Invedtigators of RCTs with expected results by 2025, as reported on ClinicalTrials.gov, in order to minimize the risk of reporting bias due to unpublished data. Another important limitation concerns the potential for immortal time bias in non-randomized studies, particularly when survival was measured from a time point preceding treatment initiation. To address this, we conducted additional analyses restricted to studies that defined survival from the start of treatment. Finally, although we performed meta-regression and sensitivity analyses to explore sources of heterogeneity and test the robustness of the findings, the large proportion of non-randomized studies and few RCTs limit causal inference; therefore the results should be interpreted as evidence of association.

In conclusion, this meta-analysis supports a clinical benefit of MTBs in the disease progression, objective and DCRs from high-quality randomized and non-randomized studies, although it underlines that in absolute terms, the number of months of disease-free gained in MTB-guided therapy patients is limited.

Evidence regarding OS remains uncertain, as MTB-guided therapies did not consistently demonstrate significant improvements, particularly in RCTs, highlighting the need for cautious interpretation.

Methodological heterogeneity and limitations in the primary evidence, and uneven implementation of MTB across settings further underscore this caution. Our review also points to the serious and critical risk of bias of observational studies that attempt to address this research question. Future well-designed RCTs and standardized reporting practices are essential to confirm these findings and to facilitate the effective integration of MTBs into clinical decision-making processes and health system strategies.

## Supporting information

S1 Checklist PRISMA Checklist. *Page MJ, McKenzie JE, Bossuyt PM, Boutron I, Hoffmann TC, Mulrow CD, et al. (2021). The PRISMA 2020 statement: An updated guideline for reporting systematic reviews. PLoS Medicine, 18(3), e1003583. This checklist is licensed under the Creative Commons Attribution 4.0 International License (CC BY 4.0; https://creativecommons.org/licenses/by/4.0/)*.
(DOCX)

S1 Appendix **Table A.** Search strings used for each database. **Table B.** Summary of outcomes, effect measures, eligible study designs and planned analyses. **Table C.** Type and source of hazard ratio (HR) estimates for overall survival (OS) and progression-free survival (PFS) in included studies and variables considered for adjustment. **Table D.** Additional characteristics of the 78 studies included. a = when both median age and mean age were reported, only median was considered. b= in Kramer, 2024,100% of patients presented 0 lines of treatment; c = in Kramer, 2024,100% of patients presented 0 lines of treatment; d = in Schneider, 2021,100% of patients presented 1 line of treatment; e = in 7 studies, data were not reportable as they did not evaluat treatment response; f = in 7 studies, data were not reportable as they did not evaluate survival time outcomes; g = totals are more than 100% since more than on option could be included in each study. **Table E.** Additional characteristics of the MTBs in the 78 studies. N.R., Not Reported; NGS, Next Generation Sequencing; IHC, Immunohistochemistry; CGH, Comparative Genomic Hybridization; FISH, Fluorescence In Situ Hybridization; t-NGS, Targeted Next Generation Sequencing; CGP, Comprehensive Genomic Profiling; WES, Whole Exome Sequencing; RNA-seq, RNA Sequencing; WGS, Whole Genome Sequencing; mRNAseq, Messenger RNA Sequencing; lcWGS, Low-Coverage Whole Genome Sequencing; l-NGS, large Next Generation Sequencing; ctDNA, circulating tumor DNA; CUP, cancer of unknow primary; NSCLC, Non-small cell lung carcinoma **Table F.** Outcome non-metanalyzed, with corresponding survival time starting points and response assessment criteria in the studies. a = data was not reportable as they did not evaluate treatment response; b = data was not reportable as they did not evaluate survival time outcomes. N.A., Not Applicable; N.R., Not Reported; N.E., Not Estimable; ORR, Overall Response Rate; CR, Complete Response; PR, Partial Response; SD, Stable Disease; PD, Progressive Disease; CB, Clinical Benefit; NE, Not Evaluated; DCR, Disease Control Rate; MR, Mixed Response; irRC, Immune-related Response Criteria; RECIST, Response Evaluation Criteria In Solid Tumors; iRECIST, Immune RECIST; mRECIST, Modified RECIST; PCWG3, Prostate Cancer Clinical Trials Working Group 3; PERCIST, PET Response Criteria in Solid Tumors; RANO, Response Assessment in Neuro-Oncology; MCBS, Magnitude of Clinical Benefit Scale, ELN, European LeukemiaNet. **Table G.** Leave-one-out sensitivity analysis for the OS. OS, Overall Survival, HR, hazard ratio, CI, confidence interval. *P*-values were derived from *Z*-tests (Wald-type tests) for pooled effects. **Table H.** Leave-one-out sensitivity analysis for the PFS. PFS, progression-free survival, HR, hazard ratio, CI, confidence interval. *P*-values were derived from *Z*-tests (Wald-type tests) for pooled effects. **Table I.** Leave-one-out sensitivity analysis for the PFS ratio ≥ 1.3. PFS, progression-free survival, CI, confidence interval. **Table J.** Leave-one-out sensitivity analysis for the RR of ORR. ORR, Objective Response Rate; RR, relative risk; CI, confidence interval. *P*-values were derived from *Z*-tests (Wald-type tests) for pooled effects. **Table K.** Leave-one-out sensitivity analysis for the RR of DCR. DCR, Disease Control Rate; RR, relative risk; CI, confidence interval. *P*-values were derived from Z-tests (Wald-type tests) for pooled effects. **Table L.** Summary of meta-regression findings for study design and other covariates across outcomes. Values represent exponentiated coefficients from mixed-effects meta-regression models, reported as odds ratios (ORs) with 95% confidence intervals (CIs). *I*^2^ indicates residual heterogeneity, and *R*^2^ represents the proportion of between-study heterogeneity explained by each model. Model 1 included study design only; Models 2–4 additionally included, respectively, year of publication, inclusion of single versus multiple tumor types, and comparator type. **Table M.** *P*-value of the Egger’s test for the meta-analyzed outcome by stratified study design. **Table N.** *P*-value of the excess significance test for the meta-analyzed outcome stratified by study design. **Table O.** Risk of Bias Assessment for Observational and Non-Randomized Clinical Trials Using ROBINS-I. **Table P.** Detail of the Risk of Bias for observational and non-randomized clinical trial studies using ROBINS-I.
(DOCX)

S2 Appendix **Fig A.** Funnel plot stratified by study design for overall survival (OS). **Fig B.** Funnel plot stratified by study design for progression-free survival (PFS). **Fig C.** Funnel plot stratified by study design for progression-free survival ratio (PFS ratio) ≥1.3. **Fig D.** Funnel plot stratified by study design for Objective Response Rate (ORR). **Fig E.** Funnel plot stratified by study design for Disease Control Rate (DCR). **Fig F.** Sensitivity meta-analysis of overall survival (OS) stratified by study design, including only studies in which survival time was measured from the start of treatment. HR, hazard ratio, CI, confidence interval. *P*-values were derived from Cochran’s *Q* test (for heterogeneity) and *Z*-tests (Wald-type tests) for pooled effects. **Fig G.** Sensitivity meta-analysis of progression-free survival (PFS) stratified by study design, including only studies in which survival time was measured from the start of treatment. HR, hazard ratio, CI, confidence interval. *P*-values were derived from Cochran’s *Q* test (for heterogeneity) and *Z*-tests (Wald-type tests) for pooled effects. **Fig H**. Sensitivity meta-analysis of progression-free survival ratio (PFS ratio) ≥1.3 stratified by study design, including only studies in which survival time was measured from the start of treatment. CI, confidence interval. *P*-values were derived from Cochran’s *Q* test (for heterogeneity). **Fig I.** Sensitivity meta-analysis of relative risk (RR) of Objective Response Rate (ORR) (panel A) and Disease Control Rate (DCR) (panel B) stratified by study design, including only studies using RECIST 1.1 criteria. CI, confidence interval; RR, relative risk. *P*-values were derived from Cochran’s *Q* test (for heterogeneity) and *Z*-tests (Wald-type tests) for pooled effects. **Fig J.** Sensitivity meta-analysis of overall survival (OS) stratified by study design, excluding studies with hazard ratios (HRs) reconstructed from Kaplan–Meier curves. HR, hazard ratio; CI, confidence interval. *P*-values were derived from Cochran’s *Q* test (for heterogeneity) and *Z*-tests (Wald-type tests) for pooled effects. **Fig K.** Sensitivity meta-analysis of progression-free survival (PFS) stratified by study design, excluding studies with hazard ratios (HRs) reconstructed from Kaplan–Meier curves. HR, hazard ratio; CI, confidence interval. *P*-values were derived from Cochran’s *Q* test (for heterogeneity) and *Z*-tests (Wald-type tests) for pooled effects. **Fig L.** Risk of bias for Randomized Controlled Trials (RCTs) using RoB 2. **Fig M.** Sensitivity meta-analysis of overall survival (OS) stratified by study design, excluding studies with serious or critical risk of bias. HR, hazard ratio; CI, confidence interval. *P*-values were derived from Cochran’s *Q* test (for heterogeneity) and *Z*-tests (Wald-type tests) for pooled effects. **Fig N.** Sensitivity meta-analysis of progression-free survival (PFS) stratified by study design, excluding studies with serious or critical risk of bias. HR, hazard ratio; CI, confidence interval. *P*-values were derived from Cochran’s *Q* test (for heterogeneity) and *Z*-tests (Wald-type tests) for pooled effects. **Fig O.** Sensitivity meta-analysis of progression-free survival ratio (PFS ratio) ≥1.3 stratified by study design, excluding studies with serious or critical risk of bias. CI, confidence interval. *P*-values were derived from Cochran’s *Q* test (for heterogeneity). **Fig P.** Sensitivity meta-analysis of relative risk (RR) Objective Response Rate (ORR) (panel A) and Disease Control Rate (DCR) (panel B) stratified by study design, excluding studies with serious or critical risk of bias. CI, confidence intervali RR, relative risk. *P*-values were derived from Cochran’s *Q* test (for heterogeneity) and *Z*-tests (Wald-type tests) for pooled effects.
(DOCX)

S1 File Excluded records at full text screening (*n* = 114). (PDF)

## Abbreviations

CIs: confidence intervals
CGP: comprehensive genomic profiling
CR: complete remission
CUP: carcinoma of unknown primary
DCR: disease control rate
HRs: hazard ratios
MR: mixed remission
MTBs: Molecular Tumor Boards
NGS: next-generation sequencing
ORs: odds ratios
ORR: objective response rate
OS: overall survival
PIs: prediction intervals
PFS: progression-free survival
PR: partial remission
PRISMA: Preferred Reporting Items for Systematic Reviews and Meta-Analyses
RCTs: randomized controlled trials
REML: restricted maximum-likelihood
ROBINS-I: Risk Of Bias In Non-randomized Studies of Interventions
RRs: relative risks
SD: stable disease
t-NGS: targeted NGS
