# Supplementary material for: Molecular Tumor Boards clinical impact on patient care and structural features: A systematic review and meta-analysis
Source: PLoS Med. 2026 Jun 9;23(6):e1005125. doi: 10.1371/journal.pmed.1005125 (PMC13274928; doi:10.1371/journal.pmed.1005125)
Supplement: S1 Appendix — Table A. Search strings used for each database. Table B. Summary of outcomes, effect measures, eligible study designs and planned analyses. Table C. Type and source of hazard ratio (HR) estimates for overall survival (OS) and progression-free survival (PFS) in included studies and variables considered for adjustment. Table D. Additional characteristics of the 78 studies included. a = when both median age and mean age were reported, only median was considered. b= in Kramer, 2024,100% of patients presented 0 lines of treatment; c = in Kramer, 2024,100% of patients presented 0 lines of treatment; d = in Schneider, 2021,100% of patients presented 1 line of treatment; e = in 7 studies, data were not reportable as they did not evaluat treatment response; f = in 7 studies, data were not reportable as they did not evaluate survival time outcomes; g = totals are more than 100% since more than on option could be included in each study. Table E. Additional characteristics of the MTBs in the 78 studies. N.R., Not Reported; NGS, Next Generation Sequencing; IHC, Immunohistochemistry; CGH, Comparative Genomic Hybridization; FISH, Fluorescence In Situ Hybridization; t-NGS, Targeted Next Generation Sequencing; CGP, Comprehensive Genomic Profiling; WES, Whole Exome Sequencing; RNA-seq, RNA Sequencing; WGS, Whole Genome Sequencing; mRNAseq, Messenger RNA Sequencing; lcWGS, Low-Coverage Whole Genome Sequencing; l-NGS, large Next Generation Sequencing; ctDNA, circulating tumor DNA; CUP, cancer of unknow primary; NSCLC, Non-small cell lung carcinoma Table F. Outcome non-metanalyzed, with corresponding survival time starting points and response assessment criteria in the studies. a = data was not reportable as they did not evaluate treatment response; b = data was not reportable as they did not evaluate survival time outcomes. N.A., Not Applicable; N.R., Not Reported; N.E., Not Estimable; ORR, Overall Response Rate; CR, Complete Response; PR, Partial Response; SD, Stable Disease; PD, Prog [file pmed.1005125.s002.docx]

**Table A.** Search strings used for each database.

| **Database** | **Search string** |
| --- | --- |
| Pubmed | (("molecular tumour board*" OR “molecular tumor board*” OR MTB OR MTBs) AND (cancer OR neoplasm* OR tumour* OR tumor* OR malignanc* OR "precision medicine" OR "personal* medicine" OR oncolog*)) |
| Scopus | (("molecular tumour board*" OR “molecular tumor board*” OR MTB OR MTBs) AND (cancer OR neoplasm* OR tumour* OR tumor* OR malignanc* OR "precision medicine" OR "personal* medicine" OR oncolog*)) |
| Web of Science | (ALL=molecular tumour board* OR ALL=molecular tumor board* OR ALL=MTB OR ALL=mtbs) AND (ALL=cancer OR ALL=neoplasm* OR ALL=tumour* OR ALL=tumor* OR ALL=malignanc* OR ALL="precision medicine" OR ALL="personal* medicine" OR ALL=oncolog*) |
| Cochrane Central Register of Controlled Trials (CENTRAL) | (molecular tumour board* OR “molecular tumor board* OR MTB OR MTBs) AND (cancer OR neoplasm* OR tumour* OR tumor* OR malignanc* OR "precision medicine" OR personal* medicine OR oncolog*) |
| ClinicalTrials.gov | ("MOLECULAR TUMOR BOARD" OR "MOLECULAR TUMOUR BOARD" OR "MOLECULAR TUMOR BOARDS" OR "MOLECULAR TUMOUR BOARDS" OR "MTB" OR "MTBS" OR "BIOLOGY BOARD") NOT Tuberculosis |

**Table B**. Summary of outcomes, effect measures, eligible study designs and planned analyses.

| **Outcome** | **Effect measure used in meta-analysis** | **Eligible study designs** | **Additional analyses / Sensitivity analyses** |
| --- | --- | --- | --- |
| Overall Survival (OS) | Hazard Ratio (HR) | - RCT  - Non-randomized clinical trials  - Observational prospective  - Observational retrospective | - Leave-one-out analysis  - Restriction to studies measuring survival from treatment initiation  - Exclusion of studies at serious/critical risk of bias  - Meta-regression |
| Progression Free Survival (PFS) | Hazard Ratio (HR) | - RCT  - Non-randomized clinical trials  - Observational prospective  - Observational retrospective | - Leave-one-out analysis  - Restriction to studies measuring survival from treatment initiation  - Exclusion of studies at serious/critical risk of bias  - Meta-regression |
| PFS ratio ≥ 1.3 | Pooled proportion | - RCT  - Non-randomized clinical trials  - Observational prospective  - Observational retrospective | - Leave-one-out analysis  - Restriction to studies measuring PFS from treatment initiation  - Exclusion of studies at serious/critical risk of bias  - Meta-regression |
| Objective Response Rate (ORR) | Relative Risk (RR) | - RCT  - Non-randomized clinical trials  - Observational prospective  - Observational retrospective | - Leave-one-out analysis  - Restriction to RECIST-based assessment  - Exclusion of studies at serious/critical risk of bias  - Meta-regression |
| Disease Control Rate (DCR) | Relative Risk (RR) | - RCT  - Non-randomized clinical trials  - Observational prospective  - Observational retrospective | - Leave-one-out analysis  - Restriction to RECIST-based assessment  - Exclusion of studies at serious/critical risk of bias  - Meta-regression |

**Table C.** Type and source of hazard ratio (HR) estimates for overall survival (OS) and progression-free survival (PFS) in included studies and variables considered for adjustment.

| **Study, year** | **Overall Survival (OS)** | **Progression Free Survival (PFS)** |
| --- | --- | --- |
| **RCT** | | |
| Le Tourneau, 2015 | Unadjusted | Unadjusted |
| Schneider, 2021 | Adjusted (age, race, body mass index, clinical tumor size, pathologic tumor size, pathologic nodal status, histologic grade, residual cancer burden classification, and prior therapy) |  |
| Andrè, 2022 | Adjusted (line of chemotherapy, disease status at randomization and group of genomic alteration) | Adjusted (line of chemotherapy, disease status at randomization and group of genomic alteration) |
| Marchetti, 2025 | Unadjusted | Unadjusted |
| Krämer, 2024 | Adjusted (gender and chemotherapy response (complete response or partial response versus stable disease)) | Adjusted (gender and chemotherapy response (complete response or partial response versus stable disease)) |
| **Non-Randomized Clinical Trial** | | |
| Debien, 2023 | Estimated from Keplan-Meier curve |  |
| Hlevjak, 2021 | Estimated from Keplan-Meier curve |  |
| Louie, 2022 | Unadjusted | Unadjusted |
| Varnier, 2019 | Estimated from Keplan-Meier curve |  |
| Billon, 2022 | Estimated from Keplan-Meier curve | Estimated from Keplan-Meier curve |
| Bertucci, 2021 |  | Estimated from Keplan-Meier curve |
| **Observational Prospective** | | |
| Charo, 2022 | Unadjusted | Unadjusted |
| Huang B, 2021 | Adjusted (age, sex, current or former smoker, stage, lives in appalachian area) |  |
| Pinet, 2023 | Unadjusted |  |
| Scheiter, 2022 | Estimated from Keplan-Meier curve | Estimated from Keplan-Meier curve |
| Kato, 2024 | Unadjusted | Unadjusted |
| Passiglia, 2024 | Estimated from Keplan-Meier curve | Estimated from Keplan-Meier curve |
| Mapendano, 2025 | Adjusted (histology, primary site, time from diagnosis, ECOG, RTk/SAS pathway) |  |
| Mosteiro, 2023 | Adjusted (sex, stage, age, histology, smoking status and TMB status) |  |
| Dalton, 2017 |  | Estimated from Keplan-Meier curve |
| Lau, 2024 | Estimated from Keplan-Meier curve | Estimated from Keplan-Meier curve |
| **Observational Retrospective** | | |
| Dorman, 2023 | Estimated from Keplan-Meier curve |  |
| El Helali, 2023 | Unadjusted |  |
| Fukada, 2023 | Estimated from Keplan-Meier curve |  |
| Hoefflin, 2018 | Estimated from Keplan-Meier curve |  |
| Slootbeek, 2022 | Adjusted (baseline characteristics) |  |
| Ida, 2022 | Estimated from Keplan-Meier curve |  |
| Kato, S., 2020 | Unadjusted | Unadjusted |
| Niogret, 2021 | Unadjusted | Unadjusted |
| Repetto, 2023 | Unadjusted | Unadjusted |
| Zhang, 2023 | Estimated from Keplan-Meier curve |  |
| Boscolo Bielo, 2025 | Adjusted (age at the time of treatment start, number of metastatic sites (1–2 vs.≥3), liver metastasis, brain metastasis, and BC subtype) | Adjusted (age at the time of treatment start, number of metastatic sites (1–2 vs.≥3), liver metastasis, brain metastasis, and BC subtype) |
| Ghanem, 2023 | Unadjusted | Unadjusted |
| Gambardella, 2021 |  | Unadjusted |
| Tarawneh, 2022 |  | Estimated from Keplan-Meier curve |
| Gremke, 2024 |  | Estimated from Keplan-Meier curve |

**Table D.** Additional characteristics of the 78 studies included

| **Variable** |  | **N (%)** |
| --- | --- | --- |
| **Year of publication** | Before 2020 | 19 (24.4%) |
|  | After 2020 | 59 (75.6%) |
| **Study Design** | Randomized Controlled Trial | 7 (9.0%) |
|  | Non-Randomized Clinical Trial | 16 (20.5%) |
|  | Observational Prospective | 20 (25.6%) |
|  | Observational Retrospective | 35 (44.9%) |
|  | **With comparator** | **42 (53.8%)** |
|  | Randomized Controlled Trial | 7 (16.7%) |
|  | Non-Randomized Clinical Trial | 7 (16.7%) |
|  | Observational Prospective | 10 (23.8%) |
|  | Observational Retrospective | 18 (42.9%) |
|  | **Without comparator** | **36 (46.2%)** |
|  | Randomized Controlled Trial | - |
|  | Non-Randomized Clinical Trial | 9 (25.0%) |
|  | Observational Prospective | 10 (27.8%) |
|  | Observational Retrospective | 17 (47.2%) |
| **Country** | USA | 19 (24,4%) |
|  | Europe | 51 (65.4%) |
|  | Germany | 17 (21.8%) |
|  | France | 16 (20.5%) |
|  | Italy | 6 (7.7%) |
|  | The Netherlands, Spain (each) | 4 (5.1%) |
|  | Denmark, Finland, Luxembourg, (grouped) | 4 (5.1%) |
|  | Others (South America, Asia, Oceania) | 8 (10.3%) |
| **Type of comparator (N=42)** | Non-target therapy group | 17 (40.5%) |
|  | Physician choice | 10 (23.8%) |
|  | Standard of Care (SoC) | 5 (11.9%) |
|  | No recommendation from MTB | 4 (9.5%) |
|  | Without actionable driver | 3 (7.1%) |
|  | Untreated target | 1 (2.4%) |
|  | Specific treatment | 1 (2.4%) |
|  | Not pursued recommendation | 1 (2.4%) |
| **Patients’ Age**^a^ | Both adult and youth patients | 15 (19.2%) |
|  | Only adult patients (≥ 18 years old) | 61 (78.2%) |
|  | N.R. | 2 (2.6%) |
| **Sex** | Mixed | 65 (83.3%) |
|  | Only Male | 1 (1.3%) |
|  | Only Female | 10 (12.8%) |
|  | N.R. | 2 (2.6%) |
| **Previous treatments** | Treatment naive^b^ | 1 (1.3%) |
|  | Both treatment naive and previously treated | 34 (43.6%) |
|  | Previously treated | 39 (50.0%) |
|  | N.R. | 4 (5.1%) |
| **Number of previous treatments (median)** | 0^c^ | 2 (2.8%) |
|  | 1^d^ | 13 (16.7%) |
|  | 2 | 20 (25.6%) |
|  | 3 | 23 (29.5%) |
|  | 4 | 3 (3.8%) |
|  | Median not reported | 17 (21.8%) |
| **Proportion of patients with ECOG 0-1** | 50% - 79.9% | 1 (1.3%) |
|  | 80% - 89.9% | 6 (7.7%) |
|  | 90% - 94.9% | 4 (5.1%) |
|  | 95% - 99.9% | 9 (11.5%) |
|  | 100% | 3 (3.8%) |
|  | N.R. | 55 (70.5%) |
| **Proportion of patients with metastasis** | 0% | 1 (1.3%) |
|  | 1%-50% | 5 (6.4%) |
|  | 50%-84% | 11 (14.1%) |
|  | 85%-99% | 8 (10.3%) |
|  | 100% | 17 (21.8%) |
|  | N.R. | 36 (46.2%) |
| **Response assessment criteria (N=71)e** | RECIST 1.1 | 48 (67.6%) |
|  | Not defined | 20 (28.2%) |
|  | Other | 3 (4.2%) |
| **Start of survival time (N=71)^f^** | **RCT (N=7)** |  |
|  | Randomization | 7 (100.0%) |
|  | **Non RCT studies (N=64)** |  |
|  | Treatment Initiation | 36 (56.3%) |
|  | Before Treatment (e.g. from consent, from diagnosis, from referral to MTB, from specimen shipment) | 13 (20.3%) |
|  | Not defined | 15 (23.4%) |
| **Clinical outcomes considered**^g^ | Objective Response Rate | 46 (59.0%) |
|  | Disease Control Rate | 46 (59.0%) |
|  | Median Progression Free Survival | 45 (57.7%) |
|  | Median Overall Survival | 43 (55.1%) |
|  | Stable Disease | 36 (46.2%) |
|  | Partial Response | 35 (44.9%) |
|  | Progressive Disease | 33 (42.3%) |
|  | Overall Survival | 31 (39.7%) |
|  | Progression Free Survival ratio | 24 (30.8%) |
|  | Progression Free Survival | 21 (26.9%) |
|  | Complete Response | 19 (24.4%) |
|  | Clinical Benefit | 9 (11.5%) |
|  | Mixed Response | 1 (1.3%) |

^a^ =when both median age and mean age were reported, only median was considered. ^b^=in Kramer, 2024 100% of patients presented 0 lines of treatment; ^c^=in Kramer, 2024 100% of patients presented 0 lines of treatment; ^d^= in Schneider, 2021 100% of patients presented 1 line of treatment; ^e^=in 7 studies, data were not reportable as they did not evaluat treatment response; ^f^=in 7 studies, data were not reportable as they did not evaluate survival time outcomes; ^g^= totals are more than 100% since more than on option could be included in each study.

**Table E.** Additional characteristics of the MTBs in the 78 studies.

| **Study** | **MTB composition** | **Frequency of gatherings** | **Guidelines used for recommendations** | **Turnaround times** | **Modality of access** | **Specific cancer(s)** | **Technology used for testing** | **NGS application** | **Liquid biopsy** |
| --- | --- | --- | --- | --- | --- | --- | --- | --- | --- |
| Le Tourneau, 2015 | Biologists, physicians, bioinformaticians, the technical platforms’ managers and pathologists | N.R. | N.R. | N.R. | Eligible patients from the same institution or clinical trial | Breast, Ovarian, lung, colorectal, cervical, Head and neck squamous cell, Sarcoma, Urothelial, Pancreatic, CUP, Esophagogastric, Adenoid cystic, Non-adenoid cystic carcinoma salivary gland tumour, Hepatocellular, Anal squamous cell, Neuroendocrine, Biliary tract, Nasopharyngeal, Cutaneous, Mesothelioma, Peritoneal, Ependymoma, Prostate, Uveal, Germline, kidney | NGS and IHC | N.R. | no |
| Belin, 2017 | N.R. | N.R. | N.R. | N.R. | Eligible patients from the same institution or clinical trial | Breast, ovarian, lung, colorectal, cervical, head and neck squamous cell, sarcoma, urothelial carcinoma, pancreatic, CUP, Esophagogastric, adenoid cystic (ACC), non-ACC salivary gland tumour, hepatocellular, anal squamous cell, neuroendocrine, biliary tract, nasopharyngeal, cutaneous, mesothelioma, peritoneal, ependymoma, prostate, uveal melanoma, germline, kidney | NGS | N.R. | no |
| Schneider, 2021 | N.R. | N.R. | ASCO and National Comprehensive Cancer Network guidelines | N.R. | Eligible patients from the same institution or clinical trial | Breast | NGS | N.R. | yes |
| Andrè, 2022 | N.R. | N.R. | ESMO/ESCAT | N.R. | Eligible patients from the same institution or clinical trial | Breast | NGS and CGH | N.R. | no |
| Krämer, 2024 | Treating investigator, referent pathologist, referent oncologist, and, when required, a genomics expert from Foundation Medicine | N.R. | N.R. | N.R. | Eligible patients from the same institution or clinical trial | CUP | NGS | CGP | yes, CPG |
| Marchetti, 2025 | Medical oncologists, pathologists, geneticists, immunologists, bioinformaticians, and other relevant specialists | Weekly | ESMO/ESCAT | N.R. | External or selected referrals | Colorectal, breast, gastric, glioblastoma, biliary tract, NSCLC, ovarian, pancreatic, melanoma, anus, others | NGS | CGP | yes, CPG |
| Sarno, 2025 | Medical oncologists with expertise in pancreatic cancer, computational biologists, and cancer biologists | N.R. | ESMO/ESCAT | N.R. | Eligible patients from the same institution or clinical trial | Pancreatic | NGS | WES | no |
| Massard, 2017 | N.R. | Weekly | N.R. | - Signed consent to biopsy: 19 days  - Biopsy to MTB: 21 days | Eligible patients from the same institution or clinical trial | Head and neck, Digestive, Lung, Bone, Melanoma, Mesothelioma and soft tissue, Breast, Gynecological female, Urological, Central nervous system, Thyroid and other endocrine glands, Ill-defined primary tumor | NGS, CGH (aCGH) and IHC | t-NGS, WES; RNA-seq; high-res CGH | no |
| Sicklick, 2019 | Oncologists, pharmacologists, cancer biologists, geneticists, surgeons, radiologists, pathologists, and bioinformatics experts | Weekly | N.R. | Molecular genetic results to treatment: 2.0 months | N.R. | Gastrointestinal and hepatopancreatobiliary, Gynecologic, Breast, Central Nervous System, Genitourinary, Head and Neck, Lung, Other | NGS | N.R. | no |
| Trédan, 2019 | Molecular biologists, medical oncologists, and pathologists | Weekly | N.R. | Inclusion to discussion: 86 days | N.R. | Colorectal, Breast, Gynecologic, Head & neck, Sarcoma, Central nervous system, Liver–pancreas–biliary, Lung, Gastro-intestinal, Kidney, Genito-urinary, CUP, Adrenals—thyroid, Melanoma, Lymphoma-myeloma, Sensory organs and skin, Neuroblastoma, Bone, Placenta, Mesenchyma | NGS and CGH (aCGH) | t-NGS | no |
| Varnier, 2019 | Clinicians and scientists | Weekly | N.R. | N.R. | N.R. | Gynecological | NGS and CGH (aCGH) | t-NGS | no |
| Reda, 2020 | N.R. | N.R. | ESMO/ESCAT | Sample reception to results: 52 days | Eligible patients from the same institution or clinical trial | Breast, colorectal, pancreatic | NGS | WES | no |
| Bertucci, 2021 | N.R. | Weekly | ESMO/ESCAT | Testing to discussion: 58 days | Eligible patients from the same institution or clinical trial | Breast, lung, prostate, ovary, pancreas, colorectal, sarcoma, endometrial, uterine cervix, liver-biliary tractu, bladder-ureter, CUP, other | NGS | t-NGS | no |
| Hlevjak, 2021 | N.R. | N.R. | N.R. | Biopsy to discussion: 55 days | Eligible patients from the same institution or clinical trial | Breast | NGS | WGS; RNA-seq | no |
| Billon, 2022 | N.R. | Weekly | Institutional guidelines | Inclusion to discussion: 53 days | Eligible patients from the same institution or clinical trial | Prostate, Bladder-Ureter, Kidney, Testicular | NGS and CGH | N.R. | no |
| Louie, 2022 | Medical, radiation, and surgical oncologists as well as radiologists, pathologists, geneticists, clinical trial coordinators, translational/basic science researchers, and bioinformaticians. | Three times per month | N.R. | Case submission to testing: 8 days | Eligible patients from the same institution or clinical trial | Colorectal | NGS | t-NGS | yes, t-NGS |
| Miller, 2022 | Medical and gynecologic oncologists, genetic counselors, pharmacists, nurses, pathologists, radiologists, and basic scientists. | N.R. | University of Kentucky Healthcare Pharmacy and Therapeutics Committee consensus guideline | N.R. | Eligible patients from the same institution or clinical trial | Lung and bronchus, NSCLC, Small cell lung, Gynaecologic, Ovarian, fallopian tube, and peritoneal, Uterine, Gynaecological, Gastroesophageal, Pancreatic, Colorectal and appendiceal, Liver, Anus , Head and neck , Thyroid gland, Leukaemia, Breast, Nonmelanoma skin, Connective/soft tissue, Urinary tract, CUP | NGS and IHC | t-NGS | yes, t-NGS |
| Martin-Romano, 2022 | Medical oncologists, geneticists, molecular biologists, and basic scientists | Weekly | ESMO/ESCAT | N.R. | N.R. | Gastrointestinal, lung, genitourinary, brain tumors, head and neck, endocrine, soft tissue sarcoma, breast, gynecologic, CUP | NGS | t-NGS, WES; RNA-seq | no |
| Debien, 2023 | N.R. | N.R. | ESMO/ESCAT | Testing to recommendation: 21 days | Eligible patients from the same institution or clinical trial | CUP, ovarian, leiomyosarcoma, cervix SCC/AC, neuroendocrine carcinoma/tumor others (in total 35 different types of tumors). | NGS, CGH and IHC | t-NGS | no |
| Ladekarl, 2023 | Medical oncologists, pathologists, clinical genetics, and molecular biologists | Weekly | N.R. | - Signed consent to discussion: 31 days  - Signed consent to biopsy: 12 days  - Biopsy to testing results: 15 days  - Testing to discussion: 2 days | External or selected referrals | Breast, Ovaries, Lungs, Brain, Prostate, Other | NGS | WES; RNA-seq | no |
| Blanc-Durand, 2024 | Organ specialists, phase 1 specialists, biologists, and geneticists. | N.R. | ESMO/ESCAT | Diagnosis to trial inclusion: 7.7 months | N.R. | Ovarian | NGS and IHC | t-NGS | yes, t-NGS |
| Sholler, 2024 | Pediatric oncologists, pharmacists, genomics experts, cancer biologists, and bioinformaticians | N.R. | N.R. | - Biopsy to testing: 10 days (average)  - Biopsy to MTB decisions: 23 days  - Biopsy to therapy 38 days | Eligible patients from the same institution or clinical trial | Neuroblastoma, CNS tumor, Ependymoma, Glioblastoma, Diffuse midline glioma, Other CNS tumors, Rare tumor, Rhabdomyosarcoma, Ewing sarcoma, Osteosarcoma, and 33 other rare tumors | NGS | WES; RNA-seq | no |
| Vitale, 2024 | Medical oncologists, radiation oncologists, surgeons, geneticists, molecular biologists, pathologists, and bioinformaticians. | Biweekly | ESMO/ESCAT | N.R. | External or selected referrals | Non-Small-Cell Lung Cancer | NGS and IHC | t-NGS; CGP | no |
| Dalton, 2017 | Adult and pediatric medical oncologists representing diverse subspecialties, molecular pathologists with NGS expertise, genetic counselors, phase I clinical trial investigators, research coordinators, a patient advocate, and medical oncology fellows | Weekly | N.R. | N.R. | Eligible patients from the same institution or clinical trial | Breast, Lung adenocarcinoma, Lung squamous carcinoma, Lung adenosquamous, Head and neck squamous carcinoma, Neuroendocrine carcinoma/small-cell carcinoma/atypical carcinoid, Salivary gland/duct, Glioblastoma/anaplastic astrocytoma, CUP, Adenoid cystic carcinoma, Hepatobiliary/ampullary/duodenal, Pancreas adenocarcinoma, Cholangiocarcinoma, Sarcoma, Endometrial, Prostate, Stomach, Colon, Ovary, Others | NGS, CGH, IHC and FISH | t-NGS | yes, t-NGS (only one pt) |
| Powell, 2018 | Medical oncologists, board-certified genetics specialists (clinical genetics, molecular genetics, cytogenetics, and genetic counselors), oncology subspecialists (radiology, pathology, interventional radiology, and surgery), clinical research, and ancillary staff (nursing, pharmacy, and patient advocates). | Twice a week | N.R. | N.R. | N.R. | Colorecta, NSCLC, breast, head and neck squamous cell, pancreatic, sarcoma, small-cell lung, esophageal, neuroendocrine, cholangiocarcinoma, thyroid, carcinoid, cervical, ovarian, prostate, endometrial. | NGS | CGP | no |
| Rodriguez-Rodriguez, 2018 | Medical oncologists, surgical oncologists, pathologists, clinical trialists, systems biologists, genetic counselors, and biomedical research scientists. | N.R. | N.R. | Testing results to therapy: 3 weeks | N.R. | Gynaecologic | NGS | CGP | no |
| Horak, 2021 | One clinician familiar with the individual case, a bioinformatician, a molecular oncologist, a medical geneticist, a pathologist, and a tumor biologist. | Twice a week | ESMO/ESCAT | Sample processing, sequencing and bioinformatics analysis: 21 days | N.R. | Bone, Breast, Colorectal, Ewing sarcoma/PNET, GIST, Gynecologic, Head and neck, Hematopoietic, Hepatopancreaticobiliary, Leiomyosarcoma, Other | NGS | WES; WGS; RNA-seq; | no |
| Huang, 2021 | Medical oncology, surgical oncology, pathology, radiology, genetic counseling, and clinical pharmacology. | Biweekly | N.R. | N.R. | Eligible patients from the same institution or clinical trial | NSCLC | NGS | N.R. | no |
| Kikuchi, 2021 | Medical oncologists, pathologists, bioinformaticians, medical geneticists, certified genetic counselors, cancer genome medical coordinators, specialists in cancer genomic medicine and attending physicias | Weekly | N.R. | - Testing to discussion: 26 days  - First outpatient visit to MTB: 43 days  - From testing to results: 14 days | N.R. | Soft-tissue sarcoma, Colorectal, Pancreas, Ovarian, Head and neck, Breast, Endometrial, Lung, Stomach, Neuroendocrine, Biliary, Urologic, Melanoma, Cervical, CUP, Thymic, Duodenal, Esophageal, Liver, Mesothelioma, Carcinosarcoma, Appendiceal, GIST, Chordoma, Small intestine, Anaplastic oligodendroglioma, Adrenocortical, Malignant transformation of a mature cystic teratoma, Anal canal. | NGS | CGP | no |
| Sultova, 2021 | Gyneco-oncologists with expertise in various cancer entities along with molecular pathologists, and genetic counselors. | N.R. | ESMO/ESCAT | Discussion to therapy: 53 days | Eligible patients from the same institution or clinical trial | Breast | NGS, IHC and FISH | t-NGS | no |
| Charo, 2022 | Medical, surgical, radiation, and gynecologic oncologists, bioinformaticians, basic and translational scientists, geneticists, clinical trial coordinators, and medication acquisition specialists. | Three times per month | N.R. | N.R. | Eligible patients from the same institution or clinical trial | Breast and gynaecologic (ovarian, endometrial, cervical, vulvar, UPS) | NGS | t-NGS | yes, t-NGS |
| Malani, 2022 | AML tumor group chair and clinicians, clinical laboratory specialists, translational scientists, bioinformaticians, study nurses, genetic counsellor for actionable germline variants (by referral) | Weekly | N.R. | N.R. | Eligible patients from the same institution or clinical trial | Acute Myeloid Leukemia | NGS | WES; RNA-seq | no |
| Scheiter, 2022 | One trained specialist in oncology, one specialist in pathology, one molecular biologist with expertise in bioinformatics, and one rotational representative of the affiliated clinics and departments | Weekly | ESMO/ESCAT | N.R. | N.R. | Colorectal, iCCA, PDAC, Gastric, Sarcoma, CUP, pCCA, Breast | NGS | N.R. | no |
| Mosteiro, 2023 | N.R. | N.R. | ESMO/ESCAT | N.R. | N.R. | Non-Small-Cell Lung Cancer | NGS | CGP | no |
| Pinet, 2023 | Molecular biologists, engineers of the PGMC, pathologists, and medical oncologists | Biweekly | ESMO/ESCAT | - Case submission to testing: 23 days  - Diagnosis to sampling: 2 years | Eligible patients from the same institution or clinical trial | Glioma, lung, breast, colorectal, prostate, pancreatic, ovarian, head and neck, kidney, gastric, biliary, uterus, cervical, others brain, esophageal, small intestine, skin, sarcoma, anal | NGS | CGP | yes, CPG |
| Renovanz, 2023 | All clinical oncology disciplines, pathologists, human geneticists, bioinformaticians, pharmacologists, and tumor biologists. | Weekly | ESMO/ESCAT | N.R. | N.R. | SNC and rare | NGS | N.R. | no |
| Boer, 2024 | Treating paediatric oncologists, early phase clinical trial oncologists, molecular pathologists, clinical geneticists, bioinformaticians, disease-specific paediatric tumour biologists from the research department | Weekly | N.R. | Sample inclusion to MTB: 30.0 working days | Eligible patients from the same institution or clinical trial | Pediatric blood cancer (leukemia - acute myeloid, acute lymphoblastic; lymphoma - non-hodgkin, hodgkin; stem cell leukemia/lymphoma) | NGS | N.R. | no |
| Kato, 2024 | N.R. | N.R. | N.R. | N.R. | N.R. | CUP | NGS | WES; mRNA-seq; RNA-seq; lcWGS | no |
| Kim, 2024 | Oncologists, pathologists, and bioinformaticians, as recommended by the Korean Society of Medical Oncology. | 5 meetings per month | N.R. | - Case submission to discussion: 7 days  - Case submission to therapy: 28 days | Eligible patients from the same institution or clinical trial | Colorectum, Lung, Breast, Other, Head and neck, Stomach, Biliary tract, Brain, MUO (Malignancy of Unknown Origin), Pancreas, Bladder, Ovary, Kidney, Uterine, Prostate, Thyroid, Adrenal gland, Liver, Skin. | NGS and IHC | t-NGS; RNA-seq | yes, t-NGS |
| Lau, 2024 | Oncologists, pathologists, clinical geneticists, genetic counselors, basic scientists, bioinformaticians and study managers, treating oncologist | N.R. | RECIST/ Percist/ RANO criteria/ National Comprehensive Cancer network Guidelines | N.R. | Eligible patients from the same institution or clinical trial | Central nervous system, solid tumours, haematologic | NGS | N.R. | no |
| Passiglia, 2024 | Thoracic oncologists, lung pathologists, molecular biologists, advocacy personnel, and data managers | N.R. | ESMO/ESCAT | - Sample reception to results: 8 days | N.R. | Non-Small-Cell Lung Cancer | NGS | t-NGS; WGS; RNA-seq | no |
| Mapendano, 2025 | Oncologists, molecular biologists, bioinformaticians, clinical geneticists, and pathologists | N.R. | N.R. | N.R. | N.R. | Breast, Lung, Ovaries, Brain, Prostate, female genital tract excluding ovaries, esophagus-stomach, head-and-neck, skin, pancreas, and urinary tract | NGS | CGP | no |
| Tsibulak, 2025 | Gynecologic oncologists, medical oncologists, pathologists, bioinformaticians, and basic scientists. Treating physicians submitted and presented patients’ clinical history | Once a month | ESMO/ESCAT | N.R. | Eligible patients from the same institution or clinical trial | Gynecological | NGS and IHC | WES; RNA-seq | yes, WES |
| Schwaederle, 2014 | Senior physician, medical oncology, surgery, radiation therapy, radiologists, clinical geneticists, pathologists, molecular diagnostics, basic and translational scientists, bioinformatics | Biweekly | N.R. | - Submission to testing: 27 days  - Test results to MTB discussion: 24 days | Eligible patients from the same institution or clinical trial | Breast, gastrointestinal, head and neck, lung, other (epithelioid sarcoma, myoepithelial carcinoma, paraganglioma, CUP). | NGS, IHC and FISH | t-NGS; WES; CGP | no |
| Parker, 2015 | Physicians, scientists, geneticists, and bioinformatics/pathway specialists attended | three times per month | N.R. | - Test ordering to reporting results: 27 days | N.R. | Breast | NGS | t-NGS | no |
| Kaderbhai, 2016 | N.R. | N.R. | N.R. | - Molecular diagnosis request to recommendation: 20 calendar days | N.R. | Lung | NGS | t-NGS | no |
| Burkard, 2017 | Basic cancer biology, medical/clinical genetics, molecular pathology, surgical pathology/ cytopathology, pharmacology, medical oncology, and radiation oncology | Biweekly | N.R. | Submission to MTB discussion: 13.5 days | External or selected referrals | Lung, Gynaecological, breast, others | NGS | N.R. | no |
| Hoefflin, 2018 | Medical and scientific experts with a focus on clinical and translational oncology and computational and molecular biology | N.R. | N.R. | Testing to recommendation: 28 days | Eligible patients from the same institution or clinical trial | Soft tissue, CNS 22, CUP, Colorectal, Urogenital, Thyroid, Breast, Lung, Hepatobiliary, Skin, Upper Gynaecological tract, Hematologic, Neuroendocrine, Pediatric, Head and neck, Others | NGS | t-NGS, WES; RNA-seq | no |
| Trivedi, 2019 | Medical and radiation oncologists, nursing, pharmacy and clinical trials staff, including a genomicist and molecular oncologist. | Biweekly | N.R. | N.R. | Eligible patients from the same institution or clinical trial | Gynecological, breast, NSCLC, CRC, other | NGS | CGP | no |
| Bitzer, 2020 | Clinical and translational oncology, pathology, bioinformatics, molecular biology, radiology, human genetics | Weekly | N.R. | N.R. | Eligible patients from the same institution or clinical trial | Gynaecological | NGS | WES | no |
| Kato, 2020 | Oncologists, surgeons, radiation oncologists, clinical trial coordinators/navigators, geneticists, pathologists, radiologists, gynaecologic oncologists, basic/translational scientists and bioinformaticians. | Three times per month | N.R. | N.R. | N.R. | Breast, colorectal, hematologic malignancies, gastroesophageal and pancreatic | NGS | t-NGS | yes, t-NGS |
| Koopman, 2020 | Pulmonary oncologists, medical oncologists, pathologists, clinical scientists in molecular pathology (molecular biologists), and structural biologists | Weekly | ASCO | N.R. | External or selected referrals | NSCLC, non-NSCLC cases, Colorectal, Melanoma, Endometrial, CUP | NGS | N.R. | no |
| Angel, 2021 | Medical oncologists, pathologist, clinical trials investigators, genetic counsellors, molecular biologists and bioinformatics professionals | Monthly | N.R. | N.R. | Eligible patients from the same institution or clinical trial | NSCLC, breast, Colorectal, Pancreatic, Cholangiocarcinoma, Metastatic castration resistant prostate, Urothelial, CUP, sarcomas, head and neck, gynaecological | NGS | CGP | no |
| Gambardella, 2021 | Clinical oncology, molecular biology, pathology, clinical genetics and bioinformatics. | Weekly | ESMO/ESCAT | N.R. | N.R. | Breast, Gynaecological, Lung, Digestive, Head and Neck, Skin, Others | NGS | t-NGS; RNA-seq | no |
| Hoefflin, 2021 | Physicians with molecular cancer expertise, experts from molecular pathology, molecular biology, and medical bioinformatics | Biweekly | N.R. | MTB discussion to recommendation: 42 days | Eligible patients from the same institution or clinical trial | Lower GI tract, Pancreas, Upper GI tract, Central nervous system, CUP, Hepatobiliary, Thyroid, Soft tissue and bone, Gynaecological (others), Head and neck, Breast, Urogenital, Ovary, Dermatologic, Hematologic, Lung, Neuroendocrine, Other | NGS and IHC | t-NGS, WES; RNA-seq | no |
| Niogret, 2021 | Clinical and translational oncology, pathology, bioinformatics, molecular biology, radiology, and human genetics. | N.R. | ESMO/ESCAT | Testing to results: 44 days | Eligible patients from the same institution or clinical trial | Lung | NGS | large NGS | no |
| Ida, 2022 | Oncologists, pediatric oncologists, pathologists, genome researchers, bioinformaticians, and genetic counselors. | Weekly | N.R. | - Testing to reccomendation: 27 days  - Recomendation to therapy: 58 days | N.R. | Colorectal, lung, soft tissue sarcoma, others | NGS | CGP | no |
| Slootbeek, 2022 | Oncologists, pathologists, clinical scientists in molecular pathology, clinical geneticists and pharmacists | Weekly | ESMO/ESCAT | N.R. | Eligible patients from the same institution or clinical trial | Prostate | NGS | t-NGS; WGS | no |
| Tarawneh, 2022 | Oncologists, radiation oncologists, surgeons, radiologists, pathologists, basic scientists, bioinformatics specialists, clinical study coordinators, patient navigators, and drug acquisition specialists | N.R. | ESMO/DKTK | N.R. | Eligible patients from the same institution or clinical trial | Lower Gynaecological tract, Neuroendocrine neoplasms, Sarcoma, Head and neck, Hepatobiliary, CUP, Pancreato, biliary, Breast, Ovary, Thoracic malignancies, Upper GI tract, Gynecologic (non-ovarian), Germ cell tumor, Embryonal (Neuroblastoma) | NGS | t-NGS; RNA Fusion Detection; lcWGS | no |
| Berclaz, 2023 | Pathologists, medical oncologists, geneticists, and representatives from organ-specific tumor boards | Weekly | ESMO/ESCAT | - Testing to MTB discussion: median 62.5 days  - Diagnosis to MTB discussion 18.7 months | Eligible patients from the same institution or clinical trial | Bone and soft tissue sarcomas (BS/STS) | NGS | CGP | no |
| Blobner, 2023 | Neuro-oncologists, neuropathologists, pathologists, hemato-oncologists, and human geneticists. | N.R. | ESMO/ESCAT | - Testing to MTB discussion: 48 days  - Completion of molecular profiling: 34 days | Eligible patients from the same institution or clinical trial | Glioblastoma, Astrocytoma, Oligodendroglioma, ependymoma with different mutations | NGS | t-NGS | no |
| Dorman, 2023 | Clinicians, pathologists, tumor geneticists, and experts on precision oncol- ogy | Weekly | N.R. | Diagnosis to MTB discussion: 5.5 months | Eligible patients from the same institution or clinical trial | Pancreatic | NGS | CGP | no |
| El Helali, 2023 | Oncologists, physicians with cancer subspecialties, molecular pathologists, cancer biologists, pharmacists, clinical geneticists, bioinformaticians, biostatisticians, and data scientists. | Monthly | ESMO/ESCAT | N.R. | N.R. | Primary central nervous system, hepato-biliary-pancreatic (HBP), thoracic, Breast, Endocrine, Gastrointestinal, Genitourinary, Gynecologic, Head & Neck, Hepato Biliary, pediatric, CNS, Sarcoma, thoracic, unknown origin. | NGS | CGP | no |
| Fukada, 2023 | Physicians specializing in cancer pharmacotherapy, expertise in genetic medicine, genetic counselors, pathologists, expertise in molecular genetics and cancer genomic medicine, those with expertise in bioinformatics | N.R. | N.R. | N.R. | N.R. | Colorectal, Pancreas, Ovary, Breast, Biliary tract, Uterine, Soft tissue sarcoma, Stomach, Lung, Esophagus, Head and neck, Prostate, CUP, Thyroid gland, Urothelium, Other | NGS | CGP | no |
| Ghanem, 2023 | Adult and pediatric medical oncologists representing diverse subspecialties, molecular pathologists with NGS expertise, genetic counselors, phase I clinical trial investigators, research coordinators, a patient advocate, and medical oncology fellows | N.R. | N.R. | N.R. | N.R. | Oligodendroglioma, high-grade astrocytoma and glioblastoma | NGS | t-NGS, WES | no |
| Giacomini, 2023 | Oncologists, molecular biologists/biotechnologists, pathologists, biostatisticians, bioinformaticians, hospital pharmacist, delegate from the Institutional Biobank, the Scientific Secretary, executive Secretary, haematologists, endocrinologist-oncologists, medical geneticist, interventional radiologists and radio- therapists, surgeons, patient advocacy delegates, nurses, data managers and representatives of the local health governance | N.R. | ESMO/ESCAT | Testing to MTB discussion: 15 days | Eligible patients from the same institution or clinical trial | Breast, NSCLC, colorectal, brain, sarcoma, melanoma, multiple cancers, pancreatic, ovarian, gastric, renal carcinoma, leukemia, medullary thyroid, salivary gland carcinoma, biliary carcinoma, holangiocarcinoma, larynx carcinoma, neuroblastoma, paraganglioma | NGS | t-NGS, WES; RNA-seq | yes, all |
| Limousin, 2023 | N.R. | N.R. | ESMO/ESCAT | N.R. | N.R. | Hepatocellular carcinoma and Hepatocholangiocarcinoma | NGS | WES; WGS; RNA-seq; | no |
| Repetto, 2023 | Oncologists, pathologists, pharmacists, radiotherapists, geneticists, bio- informaticians and psychologists. | Biweekly | ESMO/ESCAT | N.R. | Eligible patients from the same institution or clinical trial or External or selected referrals | Breast, lung and 24 other types | NGS | N.R. | no |
| Shaya, 2023 | Medical oncologists, radiation oncologists, surgeons, radiologists, pathologists, basic scientists, bioinformatics specialists, clinical study coordinators, patient navigators, and drug acquisition specialists | N.R. | N.R. | Case submission to testing: 3–4 weeks | Eligible patients from the same institution or clinical trial | Pancreatic | NGS | CGP | yes, CPG |
| Weiss, 2023 | Physicians and scientists with expertise in precision oncology. Oncologists, pathologists/ molecular pathologists, tumor geneticists, and experts for precision oncology discuss CGP results within a patient’s clinical context | N.R. | ESCAT + | - Testing results to MTB presentation: 36 days  - Diagnosis to MTB discussion 249 days (external patients), 79 days (internal patients) | Eligible patients from the same institution or clinical trial | CUP | NGS | CGP | no |
| Zhang, 2023 | N.R. | N.R. | ESMO/ESCAT | Diagnosis to testing: 155.5 days | N.R. | Biliary tract | NGS | CGP | no |
| Dang, 2024 | Expert in medical and molecular oncology, pathologist, geneticist, and medical oncologists | Every 8 weeks | OncoKB | Biopsy to MTB discussion: 12.5 months | N.R. | Adrenal, Anal carcinoma, Breast, Cholangiocarcinoma, CRC, CUP, Gastric, Glioblastoma, H&N, Kidney, Kidney (Bellini), Liver, Lung, Melanoma, Merkel cell, Mucin rich appendix ADC, Neuroendocrine, Ovarian, Pancreatic, Peritoneal PseudoMyxoma, Prostate, Renal, Sarcoma, Thyroid, Urological, Uterine cancer | NGS | t-NGS; CGP | no |
| De Jager, 2024 | Pulmonary oncologists, medical oncologists, pathologists, clinical scientists in molecular pathology (molecular biologists), and structural biologists | Weekly | N.R. | N.R. | Eligible patients from the same institution or clinical trial and External or selected referrals | Lung and melanoma | NGS | N.R. | no |
| Dreikhausen, 2024 | N.R. | N.R. | ESMO/ESCAT; OncoKB;  NCT/DKTK | Diagnosis to testing: 23.4 months | N.R. | Colorectal, Cholangiocellular carcinoma, EGJ/Gastric, Pancreatic, Hepatocellular, duodenal, neuroendocrine of the esophagogastric junction, neuroendocrine tumor of the midgut, small bowel adenocarcinoma, ampullary cancers, mixed hepatocellular-cholangiocellular carcinomas and CUP | NGS and IHC | t-NGS | no |
| Gremke, 2024 | Clinical oncologists, pathologists, bioinformaticians, human geneticists, organ specialists (e.g., gynecologists), and scientists with expertise in molecular diagnostics. | N.R. | N.R. | N.R. | N.R. | Breast, Ovary, Uterine sarcoma, Endometrium, Cervix, Vagina/Vulva, Others | NGS and IHC | N.R. | no |
| Louie, 2024 | Medical, radiation, and surgical oncologists, radiologists, and pathologists, bioinformaticians, geneticists, basic/translational scientists, medication acquisition specialists, and clinical trial coordinators/navigators | Several times per month | N.R. | N.R. | N.R. | Rare/ultra-rare (Biliary cancer, Sarcoma, Other GI malignancy, Appendiceal, Duodenal, Gynecological , Uterine , Cervical, Ovarian, Fallopian tube carcinoma, Vulvar squamous cell, CNS malignancy, Medullary thyroid , Other malignancies, CUP, Anal squamous cell, Metastatic cutaneous squamous cell, Gastroesophageal, Renal cell, Renal collecting duct, Metastatic cutaneous basal cell, Hepatocellular, Pleuropulmonary blastoma, Parotid gland, Penile squamous cell, Peritoneal, Rectal neuroendocrine, Desmoid-type fibromatosis, Undifferentiated pleomorphic sarcoma, Submandibular gland, Thymoma, Urachal, Adrenal cortical carcinoma, Sclerosing epithelioid fibrosarcoma) | NGS and IHC | t-NGS; mRNA-seq | yes, t-NGS |
| Perez, 2024 | Medical oncologists, pathologists, genetic counselors, computer scientists, and biologists | N.R. | SEOM/ESMO/ ESCAT | N.R. | Eligible patients from the same institution or clinical trial | Urothelial cancer | NGS | t-NGS | yes, t-NGS |
| Boscolo Bielo, 2025 | Oncologists, pathologists, pharmacists, radiotherapists, geneticists, bio- informaticians and psychologists | N.R. | ESMO/ESCAT | - Sample collection to testing results: 1.74 months  - Sample collection for CPG testing to MTB discussion: 4.57 months  - Tissue sample to MTB discussion: 5.06 months | N.R. | Breast | NGS | CGP | no |

N.R.= Not Reported; NGS = Next Generation Sequencing; IHC = Immunohistochemistry; CGH = Comparative Genomic Hybridization; FISH = Fluorescence In Situ Hybridization; t-NGS = Targeted Next Generation Sequencing; CGP = Comprehensive Genomic Profiling; WES = Whole Exome Sequencing; RNA-seq = RNA Sequencing; WGS = Whole Genome Sequencing; mRNAseq = Messenger RNA Sequencing; lcWGS = Low-Coverage Whole Genome Sequencing; l-NGS = large Next Generation Sequencing; ctDNA = circulating tumor DNA; CUP = cancer of unknow primary; NSCLC = Non-small cell lung carcinoma

**Table F.** Outcome non meta-analyzed, with corresponding survival time starting points and response assessment criteria in the studies.

| **Study** | **Sample size** | **Median OS (months)** | **Median PFS (months)** | **Response rate information** | **Start of survival time** | **Response assessment criteria** |
| --- | --- | --- | --- | --- | --- | --- |
| **RCT** | | | | | | |
| Le Tourneau, 2015 | MTB: 99  Comp: 95 | MTB: 9.5  Comp: 10.3 | MTB: 2.3 (95% CI 1.7–3.8)  Comp: 2.0 (95% CI 1.8–2.1) | MTB: ORR 4/98 (4.1% (95% CI 0.0–8.0))  Comp: ORR 3/89 (3.4% (95% CI 1.0–9.5)) | Randomization | RECIST 1.1 |
| Belin, 2017 | MTB: 25  Comp: 70 | N.R. | MTB: 2.1 (95% CI 2.0-3.8)  Comp: 2.0 (95% CI 1.9–2.4) | MTB: ORR 0 (0.0%)  Comp: ORR 2 (4.0% (2.8% (95% CI: 0–6.8)) | Randomization | RECIST 1.1 |
| Schneider, 2021 | MTB: 65  Comp: 70 | N.R. | N.R. | N.R. | Randomization | N.A.^b^ |
| Andre, 2022 | MTB: 157  Comp: 81 | MTB: 22.1 (95%CI 19.4-25.7)  Comp: 25.3 (95%CI 17.0-28.5) | MTB: 5.5 (95% CI: 4.0–6.9)  Comp: 2.9 (95% CI 2.3–4.8) | MTB: ORR 26 (17.1%), DCR 106 (67.5%), CR 4 (2.6%), PR 22 (14.5%), SD 80 (52.6%), PD 46 (30.3%), missing 5 (3.2%)  Comp: ORR 9 (12.9%), DCR 42 (51.9%), CR 1 (1.4%), PR 8 (11.3%), SD 33 (46.5%), PD 28 (39.4%), NE 1 (1.4%), missing 10 (12.3%) | Randomization | RECIST 1.1 |
| Krämer, 2024 | MTB: 326  Comp: 110 | MTB: 14.7 (95% CI 13.3-17.3)  Comp: 11.0 (95% CI 9.7-15.4) | MTB: 6.0 (95% CI 4.70–6.47)  Comp: 4.4 (95% CI 4.14–5.59) | MTB: ORR 58 (17.8%), DCR 211 (64.7%), CR 16 (4.9%), PR 42 (12.9%), SD 145 (44.5%), PD 58 (17.8%), Non-CR/PD 23 (7.1%), NE (CR at end of induction) 8 (2.5%), NE 2 (0.6%), missing 32 (9.8%)  Comp: ORR 9 (8.2%), DCR 66 (60.0%), CR 4 (3.6%), PR 5 (4.5%), SD 54 (49.1%), PD 24 (21.8%), Non-CR/PD 7 (6.4%), NE (CR at end of induction) 3 (2.7%), missing 32 (9.8%) | Randomization | RECIST 1.1 |
| Marchetti, 2025 | MTB: 200  Comp: 200 | MTB: 9.11 (95%CI 6.78-11.02)  Comp: 7.86 (95% CI 5.43-10.00) | MTB: 3.45 (95%CI 3.03-4.84)  Comp: 2.80 (95% CI 2.53-3.19) | MTB: ORR 35 (17.5% (95% CI: 12.5-23.5)), DCR 74 (37.0%), CR 6 (3.0%), PR 29 (14.5%), SD 39 (19.5%), PD 126 (63.0%)  Comp: ORR 20 (10.0% (95% CI: 5.6–15.0)), DCR 55 (27.5%), CR 0 (0.0%), PR 20 (10%), SD 35 (17.5%), PD 145 (72.5%) | Randomization | RECIST 1.1, irRC |
| Sarno, 2025 | 4 | 19.3 | N.R. | N.R. | Randomization | RECIST 1.1 |
| **Non-Randomized Clinical Trial** | | | | | | |
| Massard, 2017 | 194 | N.R. | N.R. | ORR 22 (11.3% (95% CI 7%–17%)) | Treatment | RECIST 1.1 |
| Sicklick, 2019 | 83 | 8.5 | 6.5 | N.R. | Treatment | RECIST 1.1 |
| Tredan, 2019 | 163 | N.R. | N.R. | ORR 23/182 (12.6%), DCR 80/182 (44.0%), PR 23/182 (12.6%), SD 57/182 (31.3%), PD 81/182 (44.5%), NE 21/182 (11.5%) | N.A. ^a^ | RECIST 1.1 |
| Varnier, 2019 | MTB: 39  Comp: 200 | MTB: 15.6 (95% CI 6.6–33)  Comp: 14.2 (95% CI 11.0–17.4) | MTB: 2.7 (95% CI 2.3–4.7) | ORR 8 (20.5%), DCR 26 (46.1%), CB 18 (46.1%), PR 8 (20.5%), SD 10 (25.6%) | MTB | RECIST 1.1 |
| Reda, 2020 | MTB: 79  Comp: 263 | N.R. | MTB: 2.5 (95% CI 2.2-3.7)  Comp: 2.4 (95% CI 2.1-3.3) | ORR 4 (5.1%), DCR 22 (27.8%), PR 4 (5.1%), SD 18 (22.8%), PD 57 (72.2%) | Treatment | RECIST 1.1 |
| Bertucci, 2021 | MTB: 94  Comp: 160 | MTB: 8.1 (95% CI 6.2–12.2)  Comp: 8.9 (95% CI 6.5–11.1) | MTB: 2.9 (95% CI 2.7-3.2)  Comp: 2.8 (95% CI 2.2–3.2) | MTB: ORR 17/89 (19.1% (95%CI 12–29)), DCR 31/89 (34.8% (95%CI 25–46)), CR 2/89 (2.2%), PR 15/89 (16.9%), SD 14/89 (15.7%), PD 58/89 (65.2%),  Comp: ORR 35/140 (25.0%), DCR 47/140 (33.6% (95%CI 26–42)), CR 2/140 (1.4%), PR 33/140 (23.6%), SD 12/140 (8.6%), PD 93/140 (66.4%) | Treatment | RECIST 1.1 |
| Hlevjak, 2021 | MTB: 64  Comp: 63 | N.R. | N.R. | N.R. | Treatment | RECIST 1.1 |
| Billon, 2022 | MTB: 12  Comp: 23 | N.R. | N.R. | MTB: ORR 4 (33.3% (95%CI 11–65)), DCR 7 (58.3% (95% CI 28%-85%)), CR 1 (8.3%), PR 3 (25.0%), SD 3 (25.0%), PD 5 (41.7%)  Comp: ORR 3 (13.0% (95%CI 3–35)), DCR 5 (21.7% (95%CI 7–44)) | Treatment | RECIST 1.1 |
| Louie, 2022 | MTB: 34  Comp: 17 | MTB: 9.3 (95% CI 3.9–14.7)  Comp: 13.1 (95% CI 0–27) | MTB: 3.9 (95% CI 1.3–6.5)  Comp: 3.1 (95% CI 1.6–4.7) | MTB: DCR 13/32 (40.6%), CB 13/32 (40.6%)  Comp: DCR 3 (17.6%), CB 3 (17.6%) | Treatment | RECIST 1.1 |
| Miller, 2022 | 93 | 768 days (range 22-1240) | 449 days (range 42-1125) | N.R. | Treatment | Not defined |
| Martin-Romano, 2022 | 136 | 13.9 (95% CI 12.7-N.E.) | 4.6 (95% CI 3.8-6.1) | ORR 36 (26.5%), DCR 82 (60.3%), PR 36 (26.5%), SD 46 (33.8%), PD 54 (39.7%) | Treatment | RECIST 1.1 |
| Debien, 2023 | MTB: 49  Comp: 50 | MTB: 11.4 (95% CI, 9–15.5)  Comp: 7.4 (95% CI 3.7–13.1) | 2.3 (95% CI 1.8–3.6) | N.R. | Treatment | RECIST 1.1 |
| Ladekarl, 2023 | 15 | N.R. | N.R. | ORR 6 (40.0%), CR 1 (6.7%), PR 5 (33.3%) | N.A.^a^ | RECIST 1.1 |
| Blanc-Durand, 2024 | 9 | N.R. | 7.6 (95%CI 3.7-N.E.) | ORR 5 (55.6%), DCR 5 (55.6%), PR 5 (55.6%), PD 4 (44.4%) | Treatment | RECIST 1.1 |
| Sholler, 2024 | 124 | N.R. | 0.26 years (95% CI 0.19-0.35) | ORR 22 (17.7%), DCR 63 (50.8%), CR 11 (8.9%), PR 11 (8.9%), SD 41 (33.1%), PD 48 (39.7%), NED 18 (14.5%) | Treatment | RECIST 1.1 |
| Vitale, 2024 | 151 | non-MTT 10.7 (95% CI 8.3-15.9), TKI 23.9 (95% CI 21.1-N.E.), MTT 23.4 (95% CI 13.6-45.5) | CT/IO 7.7 (95% CI 6.2-11.6), TKI 18.8 (95% CI 13.3-NE), MTT 14 (95% CI 4.7-N.E.) | N.R. | Not defined | Not defined |
| **Observational Prospective** | | | | | | |
| Dalton, 2017 | MTB: 24  Comp: 54 | N.R. | MTB: 5.0 (95% CI 2.9-N.E.)  Comp: 2.97 (95% CI 2.4-5.13) | N.R. | Treatment | RECIST 1.1 |
| Powell, 2018 | 39 | 44.8 weeks GMCT, 35.6 weeks FDA | GMCT 14.9 weeks, FDA 25.0 weeks | ORR 5 (12.8%), DCR 10 (25.6%), SD 14 (35.9%), PD 13 (33.3%) | Not defined | RECIST 1.1 |
| Rodriguez-Rodriguez, 2018 | 25 | N.R. | N.R. | DCR 16/23 (69.6%), (CR, PR, SD, or CB) 16/23 (69.6%) | Treatment | RECIST 1.1 |
| Horak, 2021 | 362 | 10.4 (9.2–11.6) | N.R. | ORR 87 (23.9%), DCR 201 (55.3%) | MTB | Not defined |
| Huang, 2021 | MTB: 77  Comp: 879 | N.R. | N.R. | N.R. | Not defined | N.A.^b^ |
| Kikuchi, 2021 | 21 | N.R. | N.R. | ORR 6 (28.6%), DCR 14 (66.7%) | N.A.^a^ | RECIST 1.1 |
| Sultova, 2021 | 16 | N.R. | N.R. | N.R. | Treatment | RECIST 1.1 |
| Charo, 2022 | MTB: 16  Comp: 52 | MTB: 17.1 (95% CI 10.1-24.1)  Comp: 10.8 (95% CI 8.0-26.4) | MTB: 9.3 (95% CI 8.0-10.6)  Comp: 3.4 (95% CI 2.5-4.3) | N.R. | Treatment | RECIST 1.1 |
| Malani, 2022 | 37 | 7.5 months (range 14 days–4.7+ years) | N.R. | ORR 22 (59.5%) | Not defined | ELN2017 criteria |
| Scheiter, 2022 | MTB: 38  Comp: 47 | MTB: 11.90  Comp: 6.90 | MTB: 4.20  Comp: 2.83 | MTB: ORR 7 (18.4%), DCR 19 (50.0%), CB 19 (50.0%), PR 7 (18.4%), MR 1 (2.6%), SD 11 (29.0%), PD 19 (50.0%),  Comp: CR 1 (2.1%), PR 14 (29.8%), MR 3 (6.4%), SD 12 (25.5%), PD 17 (36.2%) | MTB | Not defined |
| Mosteiro, 2023 | MTB: 28  Comp: 28 | MTB: Not reached  Comp: 27.7 (95% CI 15.02-N.E.) | N.R. | N.R. | Diagnosis | N.A.^b^ |
| Pinet, 2023 | MTB: 13  Comp: 69 | MTB: 13 (95% CI 13.0–N.E.)  Comp: 14 (95% CI 11.0–20.0) | N.R. | N.R. | Diagnosis | N.A.^b^ |
| Renovanz, 2023 | 86 | 33 (range 8– 271) | 3.3 (range 0.04–30.9) | DCR 27/70 (38.6%) | Treatment | ESMO-MCBS |
| Boer, 2024 | 24 | N.R. | N.R. | ORR 12 (50.0%), CR 12 (50.0%) | N.A.^a^ | Not defined |
| Kato, 2024 | MTB: 37  Comp: 25 | MTB: 13.4 (95% CI 7.2-19.6)  Comp: 6.9 (95% CI 4.5-9.3) | MTB: 3.7 (95% CI 1.4-6.0)  Comp: 3.8 (95% CI 1.6-6.0) | MTB: DCR 15/34 (44.1%), CB 15/34 (44.1%)  Comp: DCR 5/21 (23.8%), CB 5/21 (23.8%) | Treatment | Not defined |
| Kim, 2024 | 89 | 6.9 (95% CI 5.2−10.0) | N.R. | ORR 7/69 (10.1%), DCR 50/69 (72.5%), CR 1/69 (1.4%), PR 6/69 (8.7%), SD 43/69 (62.3%), PD 19/69 (27.5%) | Consent | RECIST 1.1 |
| Lau, 2024 | MTB: 99  Comp: 75 | N.R. | N.R. | MTB: ORR 27/90 (30.0%), DCR 61/90 (67.8%), CR 8/90 (8.9%), PR 19/90 (21.1%), SD 34/90 (37.8%), PD 29/90 (32.2%)  Comp: ORR 1/43 (2.3%), DCR 18/43 (41.9%), CR/PR 1/43 (2.3%), SD 17/43 (39.5%) | Treatment | RECIST 1.1, PERCIST, RANO |
| Passiglia, 2024 | MTB: 76  Comp: 36 | MTB: 22.3  Comp: 12.6 | MTB: 16.3  Comp: 6.9 | MTB: ORR 37 (48.7%)  Comp: ORR 11 (30.6%) | Not defined | Not defined |
| Mapendano, 2025 | MTB: 30  Comp: 53 | MTB: 15.7 (95% CI 9.2-N.E.)  Comp: 6.5 (95% CI 5.2-8.4) | N.R. | ORR 7/33 (21.2%), DCR 15/33 (45.5%), CB 15/33 (45.5%), CR 1/33 (3.0%), PR 6/33 (18.2%), SD 8/33 (24.2%) | MTB | RECIST 1.1 |
| Tsibulak, 2025 | 9 | N.R. | 12.0 (95% CI 5.0–N.E.) | PD 5 (55.6%) | Treatment | Not defined |
| **Observational Retrospective** | | | | | | |
| Schwaederle, 2014 | 12 | N.R. | N.R. | ORR 3/11 (27.3%), DCR 7/11 (63.6%), PR 3/11 (27.3%), SD 4/11 (36.4%), PD 4/11 (36.4%) | N.A.^a^ | Not defined |
| Parker, 2015 | 17 | N.R. | N.R. | N.R. | Not defined | Not defined |
| Kaderbhai, 2016 | 9 | N.R. | 4.5 | ORR (44.4%), PR for at least 4 months 4 (44.4%) | Not defined | Not defined |
| Burkard, 2017 | 9 | N.R. | N.R. | ORR 1/6 (16.7%), DCR 3/8 (37.5%), CB 3/8 (37.5%) | N.A.^a^ | RECIST 1.1 |
| Hoefflin, 2018 | MTB: 33  Comp: 72 | MTB: Not reached (95% CI 9-N.E.)  Comp: 10 (95% CI 7-17) | N.R. | ORR 11 (33.3%), DCR 14 (7.1%), PR 11 (33.3%), SD 8 (24.2%) | Not defined | RECIST 1.1 |
| Trivedi, 2019 | 12 | N.R. | N.R. | ORR 0 (0.0%), DCR 9 (66.7%), SD 9 (66.7%), PD 3 (33.3%) | N.A.^a^ | RECIST 1.1 |
| Bitzer, 2020 | 25 | 5.2 (95% CI 0.1- N.E.) | 2.8 (95% CI 1.0-9.0) | ORR 3/20 (15.0%), DCR 9/20 (45.0%), PR 3/20 (15.0%), SD 6/20 (30.0%), PD 11/20 (55.0%) | Treatment | RECIST 1.1, iRECIST, mRECIST |
| Kato, 2020 | MTB: 86  Comp: 164 | N.R. | N.R. | N.R. | Treatment | RECIST 1.1 |
| Koopman, 2020 | 25 | 10.4 (IQR 6.3-14.6) | 6.3 (IQR 3.2-10.6) | ORR 14/21 (66.7%), DCR 17/21 (81.0%), SD 3/21 (14.3%), PD 2/21 (9.5%) | Treatment | RECIST 1.1 |
| Angel, 2021 | 16 | N.R. | 15.25 | ORR 5 (31.2%), DCR 9 (56.3%), PR 5 (31.3%), SD 4 (25.0%), PD 7 (43.8%) | Not defined | Not defined |
| Gambardella, 2021 | MTB: 32  Comp: 66 | N.R. | MTB: 6.47  Comp: 2.76 | PD 10 (31.3%) | MTB | RECIST 1.1 |
| Hoefflin, 2021 | MTB: 76  Comp: 167 | MTB: 18 (95% CI 11-30)  Comp: 8 (95% CI 7-12) | N.R. | ORR 22 (28.9%), DCR 41 (53.9%), 5 CR (6.6%), 17 PR (22.4%), 19 SD (25.0%) | Not defined | RECIST 1.1 |
| Niogret, 2021 | MTB: 65  Comp: 143 | MTB: 19  Comp: 6.5 | MTB: 7  Comp: 2.5 | MTB: ORR 29 (44.6%), DCR 40 (61.5%), CR 2 (3.1%), PR 27 (41.5%), SD 11 (16.9%), PD 25 (38.5%)  COMP: ORR 19 (13.3%), DCR 43 (30.1%), CR 8 (5.6%), PR 11 (7.7%), SD 24 (16.8%), PD 101 (70.6%) | Treatment | RECIST 1.1 |
| Ida, 2022 | MTB: 51  Comp: 222 | MTB: Not reached (95% CI 12.9–N.E.)  Comp: 16.1 (95% CI 12.9–18.3) | N.R. | N.R. | Specimen shipment | N.A.^b^ |
| Slootbeek, 2022 | MTB: 63  Comp: 38 | 19.0 (IQR 14.7–23.2) | 5.3 (95% CI 3.5–7.1) | ORR 20/52 (38.5%), DCR 42/52 (80.8%), CR 2/52 (3.9%), PR 18/52 (34.6%), SD 22/52 (42.3%), PD 10/52 (19.2%) | Treatment | RECIST 1.1, PCWG3 |
| Tarawneh, 2022 | MTB: 30  Comp: 17 | N.R. | MTB: 4.3  Comp: 1.9 | DCR: 21 (70.0 %), No benefit 9 (30.0%), minor benefit 11 (36.7%), major benefit 10 (33.3%) | Not defined | Not defined |
| Berclaz, 2023 | 10 | N.R. | N.R. | N.R. | Diagnosis | Not defined |
| Blobner, 2023 | 12 | N.R. | patients SD 338 days (range 119–343), patients with PD 55 days (range 9–126) | ORR 0 (0.0%), DCR 4 (33.3%), SD 4 (33.3%), PD 4 (33.3%), NE 4 (33.3%) | Treatment | Wen et al. 2010 |
| Dorman, 2023 | MTB: 88  Comp: 90 | synchronous metastatic disease 14.1 (95% CI 10.4–17.8),  without metastases 24.6 (95% CI 20.4–28.8) | N.R. | N.R. | Diagnosis | N.A.^b^ |
| El Helali, 2023 | MTB: 77  Comp: 15 | MTB: 12.7 (95% CI 9.9–20.5)  Comp: 5.2 (95% CI 2.5–N.E.) | N.R. | ORR 22 (28.6%), DCR 50 (65.0%), CR 6 (7.8%), PR 16 (20.8%), SD 28 (36.3%), PD 27 (35.1%) | MTB | Not defined |
| Fukada, 2023 | MTB: 45  Comp: 668 | MTB: 18.0 (95% CI 7.9–28.1)  Comp: 14.2 (95% CI 12.4–16.0) | N.R. | ORR 16 (35.6%), DCR 26 (57.8%), PR 16 (35.6%), SD 10 (22.2%), PD 17 (37.8%), NE 2 (4.4%) | Not defined | RECIST 1.1 |
| Ghanem, 2023 | MTB: 18  Comp: 26 | MTB: 17.4 (95% CI 8.83-N.E.)  Comp: 14.3 (95% CI 7.37-N.E.) | MTB: 8.93 (95% CI 5.67-14.9)  Comp: 3.7 (95% CI 2.9-14.6) | MTB: ORR 8/15 (53.3%), DCR 11/15 (73.3%), PR 8/15 (53.3%), SD 3/15 (20.0%), PD 4/15 (26.7%)  Comp: ORR 2/20 (10.0%), DCR 13/20 (65.0%), PR 2/20 (10.0%), SD 11/20 (55.0%), PD 7/20 (35.0%) | Treatment | Not defined |
| Giacomini, 2023 | 22 | N.R. | N.R. | N.R. | MTB | Not defined |
| Limousin, 2023 | 9 | Not reached (95% CI N.E.-N.E.) | N.R. | DCR 3 (33.3%), SD/objective radiological response 3 (33.3%) | Treatment | RECIST, mRECIST |
| Repetto, 2023 | MTB: 76  Comp: 70 | MTB: 35.1 (95% CI N.E.)  Comp: 8.5 (95% CI 3.8-13.2) | MTB: 5.8 (95% CI 4.1-7.5)  Comp: 3.6 (95% CI 2.5-4.8) | MTB: ORR 25/67 (37.3%), DCR 42 (62.7%), CR 1/67 (1.5%), PR 24/67 (35.8%), SD 17/67 (25.4%), PD 22/67 (32.8%)  Comp: ORR 9 (12.9%), DCR 15 (21.4%), CR 1 (1.4%), PR 8 (11.4%), SD 6 (8.6%), PD 46 (65.7%) | Treatment | RECIST 1.1 |
| Shaya, 2023 | 18 | 4.8 (95% CI: 2.9 - 6.7) | 1.9 (95% CI 1.3 - 2.4) | DCR 5 (27.8%), CB 5 (27.8%) | Treatment | RECIST 1.1 |
| Weiss, 2023 | 4 | N.R. | 9 (range 1–19) | N.R. | Not defined | Not defined |
| Zhang, 2023 | MTB: 14  Comp: 56 | MTB: 19.0 (95% CI 4.86–33.14)  Comp: 8 (95% CI 6.27–9.73) | 9 (range 1–19) | ORR 8 (57.1%), DCR 9 (64.3%), PR 8 (57.1%), SD 1 (7.1%), PD 4 (28.6%), missing 1 (7.1%) | Treatment | Not defined |
| Dang, 2024 | MTB: 8  Comp: 26 | N.R. | MTB: 21 weeks (95% CI 20-N.E.)  Comp: 8 weeks (95% CI 4-N.E.) | ORR 3/8 (37.5%), DCR 6/8 (75.0%), PR 3/8 (37.5%), SD 3/8 (37.5%), PD 2/8 (25.0%) | Not defined | RECIST 1.1 |
| de Jager, 2024 | 57 | On label 17.7 (IQR 5.1-23.7), Off label 15.8 (IQR 6.4-34.2) | On label 5.1 (IQR 1.9-7.3), Off label 6.3 (IQR 2.9-14.9) | ORR 29 (50.9%), DCR 35 (61.4%), CR 3 (5.3%), PR 26 (45.6%), SD 9 (15.8%), PD 13 (22.8%), NE 3 (5.3%), missing 3 (5.3%) | Treatment | RECIST 1.1 |
| Dreikhausen, 2024 | 13 | N.R. | 8.8 (range 2.3–67.3) | N.R. | Treatment | N.A.^b^ |
| Gremke, 2024 | MTB: 23  Comp: 29 | N.R. | MTB: 5.5  Comp: 3.5 | major clinical benefit (PFSr ≥ 1.3 and PR/SD ≥ 6 months) 11 (47.8%) | Treatment | RECIST 1.1 |
| Louie, 2024 | 112 | 12.0 (95% CI 9.2-17.6) | 4.4 (95% CI 3.4-5.87) | ORR 43/106 (40.6%), DCR 47/106 (44.3%), CR 9/106 (8.5%), PR 34/106 (32.1%), SD 4/106 (3.8%), PD 62/106 (58.5%) | Treatment | RECIST 1.1 |
| Perez, 2024 | MTB: 7  Comp: 18 | MTB: 10.9 (95% CI 2.4–19.5)  Comp: 30.2 (95% CI 18.9-41.5) | MTB: 7.3 (95% CI 6.7–7.9) | ORR 3 (42.9%), DCR 6 (85.7%), PR 3 (42.9%), SD 3 (42.9%), PD 1 (14.3%) | Not defined | Not defined |
| Boscolo Bielo, 2025 | MTB: 33  Comp: 17 | MTB: 14.9 (95% CI 7.49-35)  Comp: Not reached (95% CI 6.80-N.E.) | MTB: 4.07 (95% CI 2.14-6.34)  Comp: 3.91 (95% CI 1.84-N.E.) | MTB: DCR 14 (42.4%)  Comp: DCR 7/16 (43.8%) | Treatment | RECIST 1.1 |

^a^=data was not reportable as they did not evaluate treatment response; ^b^=data was not reportable as they did not evaluate survival time outcomes;

N.A. = Not Applicable, N.R. = Not Reported, N.E. = Not Estimable, ORR = Overall Response Rate, CR = Complete Response, PR = Partial Response, SD = Stable Disease, PD = Progressive Disease, CB = Clinical Benefit, NE = Not Evaluated, DCR = Disease Control Rate, MR = Mixed Response, irRC = Immune-related Response Criteria, RECIST = Response Evaluation Criteria In Solid Tumors, iRECIST = Immune RECIST, mRECIST = Modified RECIST, PCWG3 = Prostate Cancer Clinical Trials Working Group 3, PERCIST = PET Response Criteria in Solid Tumors , RANO = Response Assessment in Neuro-Oncology, MCBS = Magnitude of Clinical Benefit Scale, ELN = European LeukemiaNet

**Table G.** Leave-one-out sensitivity analysis for the OS.

|  | **HR** | **95% CI** | **p-value** | **I²** |
| --- | --- | --- | --- | --- |
| **RCT** |  |  |  |  |
| Le Tourneau, 2015 | 0.88 | [0.75-1.04] | 0.126 | 0.0 |
| Schneider, 2021 | 0.88 | [0.76-1.03] | 0.102 | 0.0 |
| Andrè, 2022 | 0.86 | [0.73-1.00] | 0.056 | 0.0 |
| Krämer, 2024 | 0.89 | [0.75-1.06] | 0.197 | 0.0 |
| Marchetti, 2025 | 0.85 | [0.71-1.02] | 0.077 | 0.0 |
| Pooled estimate | 0.87 | [0.76-1.01] | 0.069 | 0.0 |
| **Non-randomized clinical trial** |  |  |  |  |
| Varnier, 2019 | 0.79 | [0.59-1.05] | 0.106 | 0.0 |
| Hlevjak, 2021 | 0.85 | [0.64-1.12] | 0.237 | 0.0 |
| Billon, 2022 | 0.85 | [0.66-1.10] | 0.209 | 0.0 |
| Louie, 2022 | 0.82 | [0.63-1.07] | 0.139 | 0.0 |
| Debien, 2023 | 0.82 | [0.61-1.09] | 0.169 | 0.0 |
| Pooled estimate | 0.83 | [0.65-1.05] | 0.124 | 0.0 |
| **Observational Prospective** |  |  |  |  |
| Huang B, 2021 | 0.67 | [0.50-0.92] | 0.012 | 50.6 |
| Charo, 2022 | 0.57 | [0.35-0.93] | 0.023 | 76.9 |
| Scheiter, 2022 | 0.50 | [0.33-0.77] | 0.002 | 68.4 |
| Pinet, 2023 | 0.52 | [0.33-0.83] | 0.006 | 76.0 |
| Mosteiro, 2023 | 0.59 | [0.37-0.95] | 0.028 | 75.3 |
| Kato, 2024 | 0.54 | [0.33-0.89] | 0.015 | 77.3 |
| Lau, 2024 | 0.54 | [0.32-0.88] | 0.013 | 76.5 |
| Passiglia, 2024 | 0.54 | [0.33-0.89] | 0.016 | 77.2 |
| Mapendano, 2025 | 0.58 | [0.35-0.95] | 0.030 | 75.5 |
| Pooled estimate | 0.56 | [0.36-0.86] | 0.009 | 74.1 |
| **Observational retrospective** |  |  |  |  |
| Hoefflin, 2018 | 0.62 | [0.56-0.69] | 0.000 | 14.0 |
| Kato, S., 2020 | 0.61 | [0.55-0.68] | 0.000 | 15.9 |
| Niogret, 2021 | 0.63 | [0.57-0.71] | 0.000 | 2.6 |
| Slootbeek, 2022 | 0.61 | [0.52-0.71] | 0.000 | 18.6 |
| Ida, 2022 | 0.61 | [0.55-0.68] | 0.000 | 16.6 |
| El Helali, 2023 | 0.63 | [0.56-0.70] | 0.000 | 0.0 |
| Fukada, 2023 | 0.60 | [0.54-0.67] | 0.000 | 0.0 |
| Repetto, 2023 | 0.63 | [0.56-0.70] | 0.000 | 5.5 |
| Zhang, 2023 | 0.62 | [0.56-0.69] | 0.000 | 15.5 |
| Dorman, 2023 | 0.62 | [0.55-0.70] | 0.000 | 18.9 |
| Ghanem, 2023 | 0.61 | [0.55-0.68] | 0.000 | 13.2 |
| Boscolo Bielo, 2025 | 0.61 | [0.55-0.68] | 0.000 | 12.8 |
| Pooled estimate | 0.62 | [0.56-0.69] | 0.000 | 10.8 |

OS = Overall Survival, HR = hazard ratio, CI = confidence interval. P-values were derived from Z-tests (Wald-type tests) for pooled effects.

**Table H.** Leave-one-out sensitivity analysis for the PFS.

|  | **HR** | **95% CI** | **p-value** | **I²** |
| --- | --- | --- | --- | --- |
| **RCT** |  |  |  |  |
| Le Tourneau, 2015 | 0.70 | [0.61-0.81] | 0.000 | 0.0 |
| Andrè, 2022 | 0.73 | [0.63-0.84] | 0.000 | 12.7 |
| Krämer, 2024 | 0.74 | [0.62-0.89] | 0.001 | 15.8 |
| Marchetti, 2025 | 0.78 | [0.66-0.92] | 0.003 | 0.0 |
| Pooled estimate | 0.73 | [0.64-0.84] | 0.000 | 0.0 |
| **Non-randomized clinical trial** |  |  |  |  |
| Bertucci, 2021 | 0.54 | [0.33-0.87] | 0.012 | 0.0 |
| Billon, 2022 | 0.72 | [0.50-1.04] | 0.082 | 30.9 |
| Louie, 2022 | 0.77 | [0.60-1.00] | 0.050 | 0.0 |
| Pooled estimate | 0.70 | [0.51-0.96] | 0.025 | 3.8 |
| **Observational Prospective** |  |  |  |  |
| Dalton, 2017 | 0.73 | [0.60-0.90] | 0.003 | 0.0 |
| Charo, 2022 | 0.77 | [0.63-0.94] | 0.011 | 0.0 |
| Scheiter, 2022 | 0.73 | [0.59-0.90] | 0.004 | 0.0 |
| Kato, 2024 | 0.73 | [0.59-0.89] | 0.002 | 0.0 |
| Lau, 2024 | 0.68 | [0.54-0.85] | 0.001 | 0.0 |
| Passiglia, 2024 | 0.74 | [0.60-0.91] | 0.004 | 0.0 |
| Pooled estimate | 0.73 | [0.60-0.88] | 0.001 | 0.0 |
| **Observational retrospective** |  |  |  |  |
| Kato, S., 2020 | 0.62 | [0.51-0.74] | 0.000 | 8.1 |
| Gambardella, 2021 | 0.64 | [0.55-0.76] | 0.000 | 5.3 |
| Niogret, 2021 | 0.66 | [0.55-0.78] | 0.000 | 2.3 |
| Tarawneh, 2022 | 0.65 | [0.56-0.77] | 0.000 | 0.0 |
| Repetto, 2023 | 0.63 | [0.53-0.74] | 0.000 | 10.5 |
| Ghanem, 2023 | 0.63 | [0.54-0.74] | 0.000 | 7.7 |
| Gremke, 2024 | 0.64 | [0.54-0.75] | 0.000 | 12.1 |
| Boscolo Bielo, 2025 | 0.61 | [0.52-0.72] | 0.000 | 0.0 |
| Pooled estimate | 0.63 | [0.54-0.74] | 0.000 | 0.0 |

PFS = Progression Free Survival, HR = hazard ratio, CI = confidence interval. P-values were derived from Z-tests (Wald-type tests) for pooled effects.

**Table I.** Leave-one-out sensitivity analysis for the PFS ratio ≥ 1.3.

|  | **Proportion** | **95% CI** | **I²** |
| --- | --- | --- | --- |
| **RCT** |  |  |  |
| **Non-randomized clinical trial** |  |  |  |
| Massard, 2017 | 0.333 | [0.259-0.412] | 54.4 |
| Sicklick, 2019 | 0.341 | [0.274-0.412] | 50.2 |
| Reda, 2020 | 0.342 | [0.278-0.409] | 48.5 |
| Bertucci, 2021 | 0.327 | [0.256-0.402] | 53.3 |
| Hlevjak, 2021 | 0.336 | [0.267-0.409] | 53.9 |
| Billon, 2022 | 0.329 | [0.267-0.393] | 53.0 |
| Miller, 2022 | 0.309 | [0.269-0.351] | 0.0 |
| Debien, 2023 | 0.340 | [0.274-0.410] | 50.7 |
| Pooled estimate | 0.331 | [0.272-0.393] | 47.0 |
| **Observational Prospective** |  |  |  |
| Horak, 2021 | 0.408 | [0.301-0.519] | 37.8 |
| Sultova, 2021 | 0.375 | [0.328-0.423] | 8.9 |
| Scheiter, 2022 | 0.384 | [0.336-0.433] | 36.7 |
| Renovanz, 2023 | 0.394 | [0.343-0.446] | 16.5 |
| Lau, 2024 | 0.378 | [0.330-0.428] | 36.3 |
| Tsibulak, 2025 | 0.377 | [0.331-0.425] | 0.0 |
| Pooled estimate | 0.381 | [0.335-0.429] | 22.6 |
| **Observational Retrospective** |  |  |  |
| Parker, 2015 | 0.435 | [0.351-0.521] | 0.0 |
| Hoefflin, 2018 | 0.418 | [0.338-0.500] | 0.0 |
| Gambardella, 2021 | 0.450 | [0.363-0.538] | 0.0 |
| Berclaz, 2023 | 0.420 | [0.341-0.501] | 0.0 |
| Giacomini, 2023 | 0.425 | [0.343-0.508] | 0.0 |
| Repetto, 2023 | 0.462 | [0.366-0.560] | 0.0 |
| Zhang D., 2023 | 0.419 | [0.339-0.500] | 0.0 |
| Blobner, 2023 | 0.440 | [0.361-0.520] | 0.0 |
| Dang, 2024 | 0.421 | [0.343-0.501] | 0.0 |
| Pooled estimate | 0.430 | [0.353-0.509] | 0.0 |

PFS = Progression Free Survival, CI = confidence interval.

**Table J.** Leave-one-out sensitivity analysis for the RR of ORR.

|  | **RR** | **95% CI** | **p-value** | **I²** |
| --- | --- | --- | --- | --- |
| **RCT** |  |  |  |  |
| Le Tourneau, 2015 | 1.79 | [1.26-2.54] | 0.001 | 0.0 |
| Andrè, 2022 | 1.84 | [1.24-2.72] | 0.002 | 0.0 |
| Krämer, 2024 | 1.62 | [1.08-2.41] | 0.018 | 0.0 |
| Marchetti, 2025 | 1.75 | [1.10-2.77] | 0.018 | 0.0 |
| Pooled estimate | 1.75 | [1.24-2.47] | 0.001 | 0.0 |
| **Non-randomized clinical trial** |  |  |  |  |
| Bertucci, 2021 | 2.56 | [0.68-9.61] | 0.165 |  |
| Billon, 2022 | 0.76 | [0.46-1.28] | 0.306 |  |
| Pooled estimate | 1.19 | [0.38-3.72] | 0.765 | 64.0 |
| **Observational Prospective** |  |  |  |  |
| Scheiter, 2022 | 3.63 | [0.49-26.93] | 0.207 | 75.3 |
| Lau, 2024 | 1.00 | [0.37-2.70] | 1.000 | 76.8 |
| Passiglia, 2024 | 2.38 | [0.11-49.48] | 0.575 | 87.9 |
| Pooled estimate | 1.84 | [0.39-8.76] | 0.444 | 79.7 |
| **Observational retrospective** |  |  |  |  |
| Niogret J., 2021 | 3.26 | [1.77-6.04] | 0.000 | 0.0 |
| Ghanem, 2023 | 3.19 | [2.13-4.78] | 0.000 | 0.0 |
| Repetto, 2023 | 3.54 | [2.21-5.66] | 0.000 | 0.0 |
| Pooled estimate | 3.32 | [2.25-4.89] | 0.000 | 0.0 |

ORR = Objective Response Rate, RR = Relative Risk, CI = confidence interval. P-values were derived from Z-tests (Wald-type tests) for pooled effects.

**Table K.** Leave-one-out sensitivity analysis for the RR of DCR.

|  | **RR** | **95% CI** | **p-value** | **I²** |
| --- | --- | --- | --- | --- |
| **RCT** |  |  |  |  |
| Andrè, 2022 | 1.17 | [0.95-1.44] | 0.144 | 39.7 |
| Krämer, 2024 | 1.32 | [1.10-1.58] | 0.003 | 0.0 |
| Marchetti, 2025 | 1.16 | [0.97-1.39] | 0.100 | 37.1 |
| Pooled estimate | 1.20 | [1.03-1.40] | 0.018 | 19.9 |
| **Non-randomized clinical trial** |  |  |  |  |
| Bertucci, 2021 | 2.52 | [1.25-5.10] | 0.010 | 0.0 |
| Billon, 2022 | 1.29 | [0.64-2.60] | 0.471 | 44.1 |
| Louie, 2022 | 1.52 | [0.61-3.78] | 0.371 | 72.2 |
| Pooled estimate | 1.62 | [0.83-3.20] | 0.160 | 58.8 |
| **Observational Prospective** |  |  |  |  |
| Scheiter, 2022 | 1.66 | [1.17-2.34] | 0.004 | 0.0 |
| Lau, 2024 | 1.10 | [0.48-2.52] | 0.815 | 69.2 |
| Kato, 2024 | 1.13 | [0.55-2.30] | 0.742 | 85.6 |
| Pooled estimate | 1.26 | [0.73-2.18] | 0.412 | 75.3 |
| **Observational retrospective** |  |  |  |  |
| Niogret J., 2021 | 1.50 | [0.76-2.97] | 0.244 | 80.7 |
| Ghanem, 2023 | 1.88 | [1.07-3.33] | 0.029 | 70.0 |
| Repetto, 2023 | 1.39 | [0.86-2.23] | 0.176 | 70.4 |
| Boscolo Bielo, 2025 | 1.88 | [1.12-3.18] | 0.017 | 76.6 |
| Pooled estimate | 1.65 | [1.02-2.68] | 0.042 | 74.7 |

DCR = Disease Control Rate, RR = Relative Risk, CI = confidence interval. P-values were derived from Z-tests (Wald-type tests) for pooled effects.

**Table L.** Summary of meta-regression findings for study design and other covariates across outcomes

|  |  | **OS** |  |  |  | **PFS** |  |  |  | **PFS ratio ≥ 1.3** |  |  | **ORR** |  |  |  | **DCR** |  |  |
| --- | --- | --- | --- | --- | --- | --- | --- | --- | --- | --- | --- | --- | --- | --- | --- | --- | --- | --- | --- |
| **Covariate** | **Model 1** | **Model 2** | **Model 3** | **Model 4** | **Model 1** | **Model 2** | **Model 3** | **Model 4** | **Model 1** | **Model 2** | **Model 3** | **Model 1** | **Model 2** | **Model 3** | **Model 4** | **Model 1** | **Model 2** | **Model 3** | **Model 4** |
| **Study design** |  |  |  |  |  |  |  |  |  |  |  |  |  |  |  |  |  |  |  |
| RCT | Ref | Ref | Ref | Ref | Ref | Ref | Ref | Ref | Ref | Ref | Ref | Ref | Ref | Ref | Ref | Ref | Ref | Ref | Ref |
| Non-randomized clinical trial | 0.94  (0.69-1.30) | 0.92  (0.65-1.30) | 0.99  (0.70-1.40) | 0.99  (0.64-1.53) | 1.00  (0.76-1.31) | 1.00  (0.76-1.31) | 0.98  (0.75-1.30) | 1.03  (0.78-1.36) | 0.96  (0.83-1.12) | 0.96  (0.82-1.13) | 0.99  (0.82-1.20) | 0.54  (0.27-1.10) | 0.60  (0.25-1.46) | 0.65  (0.31-1.36) | 0.53  (0.27-1.04) | 1.24  (0.62-2.48) | 1.28  (0.61-2.69) | 1.30  (0.54-3.12) | 1.05  (0.60-1.84) |
| Observational prospective | 0.71  (0.54-0.93) | 0.67  (0.49-0.91) | 0.70  (0.52-0.94) | 0.72  (0.51-1.02) | 1.00  (0.79-1.26) | 1.00  (0.79-1.26) | 1.00  (0.79-1.26) | 1.04  (0.82-1.33) | 1.02  (0.88-1.20) | 1.02  (0.87-1.21) | 1.05  (0.86-1.27) | 0.74  (0.39-1.42) | 0.76  (0.34-1.71) | 0.74  (0.40-1.38) | 0.64  (0.34-1.20) | 1.01  (0.54-1.89) | 1.03  (0.51-2.08) | 1.02  (0.51-2.03) | 0.66  (0.35-1.23) |
| Observational retrospective | 0.70  (0.56-0.88) | 0.68  (0.53-0.87) | 0.73  (0.57-0.93) | 0.71  (0.54-0.92) | 0.87  (0.71-1.06) | 0.87  (0.71-1.06) | 0.86  (0.70-1.05) | 0.88  (0.72-1.08) | 1.08  (0.91-1.27) | 1.07  (0.90-1.27) | 0.94  (0.80-1.12) | 1.92  (1.04-3.55) | 1.95  (0.90-4.22) | 2.19  (1.18-4.06) | 1.92  (1.07-3.43) | 1.36  (0.77-2.40) | 1.34  (0.72-2.51) | 1.36  (0.71-2.60) | 1.38  (0.89-2.15) |
| **Additional covariates** |  |  |  |  |  |  |  |  |  |  |  |  |  |  |  |  |  |  |  |
| Year of publication | - | 0.96  (0.91-1.01) | - | - | - | 0.98  (0.80-1.22) | - | - | - | 1.04  (0.92-1.17) | - | - | 1.04  (0.53-2.04) | - | - | - | 1.04  (0.58-1.86) | - | - |
| Single vs multiple tumor types | - | - | 0.88  (0.72-1.08) | - | - | - | 0.99  (0.96-1.02) | - | - | - | 1.01  (0.99-1.03) | - | - | 1.10  (0.95-1.26) | - | - | - | 1.01  (0.82-1.25) | - |
| Non-target therapy vs other | - | - | - | 0.95  (0.72-1.26) | - | - | - | 0.82  (0.60-1.13) |  |  |  | - | - | - | 11.57  (1.49-89.70) | - | - | - | 2.03  (1.04-3.98) |
| **Model fit** |  |  |  |  |  |  |  |  |  |  |  |  |  |  |  |  |  |  |  |
| **I²** | 17.3% | 27.9% | 25.4% | 29.8% | 0% | 0% | 0% | 0% | 21.9% | 26.7% | 26.6% | 18.4% | 38.4% | 13.2% | 13.9% | 76.4% | 76.4% | 77.9% | 61.2% |
| **R²** | 79.2% | 59.9% | 65.2% | 43.4% | 0% | 0% | 0% | 0% | 0% | 0% | 0% | 86.3% | 59.3% | 90.9% | 91.1% | 0% | 0% | 0% | 39.9% |

Values represent exponentiated coefficients from mixed-effects meta-regression models, reported as odds ratios (ORs) with 95% confidence intervals (CIs). I² indicates residual heterogeneity, and R² represents the proportion of between-study heterogeneity explained by each model. Model 1 included study design only; Models 2-4 additionally included, respectively, year of publication, inclusion of single versus multiple tumor types, and comparator type.

**Table M.** P-value of the Egger’s test for the meta-analyzed outcome by stratified study design

|  | **RCT** | **Non-Randomized Clinical Trial** | **Observational Prospective** | **Observational Retrospective** |
| --- | --- | --- | --- | --- |
| **OS** | 0.630 | 0.483 | 0.120 | 0.585 |
| **PFS** | 0.179 | 0.159 | 0.195 | 0.710 |
| **PFS ratio ≥ 1.3** | - | 0.636 | 0.139 | 0.141 |
| **ORR RR** | 0.638 | - | 0.144 | 0.599 |
| **DCR RR** | 0.947 | 0.923 | 0.902 | 0.861 |

**Table N.** P-value of the excess significance test for the meta-analyzed outcome stratified by study design

|  | **RCT** | **Non-Randomized Clinical Trial** | **Observational Prospective** | **Observational Retrospective** |
| --- | --- | --- | --- | --- |
| **OS** | 0.964 | 0.940 | 0.941 | 0.504 |
| **PFS** | 0.943 | 0.548 | 0.942 | 0.954 |
| **ORR RR** | 0.177 | 0.945 | 0.551 | 0.080 |
| **DCR RR** | 0.017 | 0.295 | 0.403 | 0.877 |

**Table O.** Risk of Bias Assessment for Observational and Non-Randomized Clinical Trials Using ROBINS-I

| **Studies with comparator (N=35)** | | | | | | | | | |
| --- | --- | --- | --- | --- | --- | --- | --- | --- | --- |
|  | **ROBINS-I domains** | | | | | | | | |
| **Judgement** | Bias due to confounding | Bias in selection of participants into the study | Bias in classification of interventions | Bias due to deviations from intended interventions | Bias due to missing data | Bias in measurement of outcomes | Bias in selection of the reported result | Overall Bias |  |
| Low | 10 (28.6%) | 13 (37.1%) | 28 (80.0%) | 4 (11.4%) | 17 (48.6%) | 35 (100.0%) | 14 (40.0%) | 1 (2.9%) |  |
| Moderate | 8 (22.9%) | 4 (11.4%) | 7 (20.0%) | 20 (57.1%) | 3 (8.6%) | 0 (0.0%) | 18 (51.4%) | 9 (25.7%) |  |
| Serious | 17 (48.6%) | 18 (51.4%) | 0 (0.0%) | 11 (31.4%) | 14 (40.0%) | 0 (0.0%) | 3 (8.6%) | 24 (68.6%) |  |
| Critical | 0 (0.0%) | 0 (0.0%) | 0 (0.0%) | 0 (0.0%) | 1 (2.9%) | 0 (0.0%) | 0 (0.0%) | 1 (2.9%) |  |
| **Studies without comparator (N=36)** | | | | | | | | | |
|  | **ROBINS-I domains** | | | | | | | | |
| Low | NA | 8 (22.2%) | 30 (83.3%) | 2 (5.6%) | 5 (13.9%) | 32 (88.9%) | 18 (50.0%) | 0 (0.0%) |  |
| Moderate | NA | 5 (13.9%) | 4 (11.1%) | 10 (27.8%) | 15 (41.7%) | 4 (11.1%) | 13 (36.1%) | 3 (8.3%) |  |
| Serious | NA | 22 (61.1%) | 2 (5.6%) | 23 (63.9%) | 15 (41.7%) | 0 (0.0%) | 5 (13.9%) | 30 (83.3%) |  |
| Critical | NA | 1 (2.8%) | 0 (0.0%) | 1 (2.8%) | 1 (2.8%) | 0 (0.0%) | 0 (0.0%) | 3 (8.3%) |  |

**Table P.** Detail of the Risk of Bias for observational and non-randomized clinical trial studies using ROBINS-I.

| **Studies with comparator (n=35)** | | | | | | | | |
| --- | --- | --- | --- | --- | --- | --- | --- | --- |
| **Study** | **Bias due to confounding** | **Bias in selection of participants into the study** | **Bias in classification of interventions** | **Bias due to deviations from intended interventions** | **Bias due to missing data** | **Bias in measurement of outcomes** | **Bias in selection of the reported result** | **Overall Bias** |
| **Boscolo Bielo, 2025** | Moderate | Serious | Low | Serious | Moderate | Low | Low | Serious |
| **Mapendano, 2025** | Moderate | Serious | Low | Moderate | Low | Low | Low | Serious |
| **Dang, 2024** | Serious | Serious | Low | Moderate | Critical | Low | Moderate | Critical |
| **Gremke, 2024** | Serious | Serious | Low | Serious | Low | Low | Moderate | Serious |
| **Kato, 2024** | Serious | Serious | Low | Moderate | Serious | Low | Low | Serious |
| **Lau, 2024** | Serious | Serious | Low | Serious | Moderate | Low | Moderate | Serious |
| **Passiglia, 2024** | Serious | Serious | Low | Serious | Low | Low | Moderate | Serious |
| **Perez, 2024** | Serious | Serious | Low | Serious | Low | Low | Moderate | Serious |
| **Debien, 2023** | Moderate | Low | Low | Moderate | Low | Low | Serious | Serious |
| **Dorman, 2023** | Serious | Serious | Low | Moderate | Serious | Low | Moderate | Serious |
| **El Helali, 2023** | Serious | Serious | Low | Moderate | Serious | Low | Moderate | Serious |
| **Fukada, 2023** | Moderate | Low | Low | Moderate | Low | Low | Low | Moderate |
| **Ghanem, 2023** | Serious | Serious | Moderate | Serious | Serious | Low | Serious | Serious |
| **Mosteiro, 2023** | Moderate | Serious | Low | Serious | Low | Low | Moderate | Serious |
| **Pinet, 2023** | Moderate | Moderate | Low | Moderate | Serious | Low | Moderate | Serious |
| **Repetto, 2023** | Low | Low | Low | Low | Low | Low | Moderate | Moderate |
| **Zhang, 2023** | Moderate | Moderate | Moderate | Moderate | Low | Low | Low | Moderate |
| **Billon, 2022** | Moderate | Low | Moderate | Moderate | Low | Low | Low | Moderate |
| **Charo, 2022** | Low | Low | Low | Moderate | Low | Low | Low | Moderate |
| **Ida, 2022** | Serious | Serious | Low | Moderate | Serious | Low | Moderate | Serious |
| **Louie, 2022** | Low | Moderate | Low | Moderate | Low | Low | Low | Moderate |
| **Scheiter, 2022** | Low | Low | Moderate | Low | Low | Low | Moderate | Moderate |
| **Slootbeek, 2022** | Serious | Serious | Low | Moderate | Serious | Low | Moderate | Serious |
| **Tarawneh, 2022** | Low | Low | Moderate | Serious | Serious | Low | Low | Serious |
| **Bertucci, 2021** | Low | Low | Moderate | Moderate | Low | Low | Low | Moderate |
| **Gambardella, 2021** | Serious | Serious | Low | Moderate | Serious | Low | Moderate | Serious |
| **Hlevjak, 2021** | Low | Low | Moderate | Moderate | Serious | Low | Low | Serious |
| **Hoefflin, 2021** | Serious | Serious | Low | Serious | Serious | Low | Serious | Serious |
| **Huang, 2021** | Serious | Moderate | Low | Low | Low | Low | Moderate | Serious |
| **Niogret, 2021** | Low | Low | Low | Moderate | Low | Low | Low | Moderate |
| **Kato,S. 2020** | Serious | Serious | Low | Moderate | Serious | Low | Moderate | Serious |
| **Reda, 2020** | Low | Low | Low | Moderate | Moderate | Low | Low | Moderate |
| **Varnier, 2019** | Low | Low | Low | Serious | Serious | Low | Low | Serious |
| **Hoefflin, 2018** | Serious | Serious | Low | Serious | Serious | Low | Moderate | Serious |
| **Dalton, 2017** | Serious | Low | Low | Moderate | Moderate | Low | Moderate | Serious |
| **Studies withouth comparator (N=36)** | | | | | | | | |
| **Study** | **Bias due to confounding** | **Bias in selection of participants into the study** | **Bias in classification of interventions** | **Bias due to deviations from intended interventions** | **Bias due to missing data** | **Bias in measurement of outcomes** | **Bias in selection of the reported result** | **Overall Bias** |
| **Tsibulak, 2025** | NI | Low | Low | Moderate | Serious | Low | Moderate | Serious |
| **Blanc-Durand, 2024** | NI | Low | Moderate | Serious | Serious | Low | Moderate | Serious |
| **Boer, 2024** | NI | Low | Low | Moderate | Serious | Low | Low | Serious |
| **de Jager, 2024** | NI | Serious | Low | Moderate | Low | Low | Moderate | Serious |
| **Dreikhausen, 2024** | NI | Moderate | Low | Serious | Critical | Low | Moderate | Critical |
| **Kim, 2024** | NI | Low | Low | Moderate | Serious | Low | Serious | Serious |
| **Louie, 2024** | NI | Serious | Low | Low | Serious | Low | Serious | Serious |
| **Sholler, 2024** | NI | Low | Low | Low | Moderate | Low | Low | Moderate |
| **Vitale, 2024** | NI | Low | Low | Moderate | Low | Low | Low | Moderate |
| **Berclaz, 2023** | NI | Serious | Low | Serious | Moderate | Low | Low | Serious |
| **Blobner, 2023** | NI | Moderate | Low | Moderate | Serious | Low | Moderate | Serious |
| **Giacomini, 2023** | NI | Serious | Low | Serious | Moderate | Low | Low | Serious |
| **Ladekarl, 2023** | NI | Serious | Moderate | Serious | Serious | Low | Moderate | Serious |
| **Limousin, 2023** | NI | Low | Low | Moderate | Serious | Low | Low | Serious |
| **Renovanz, 2023** | NI | Serious | Low | Serious | Serious | Low | Moderate | Serious |
| **Shaya, 2023** | NI | Serious | Low | Moderate | Moderate | Low | Low | Serious |
| **Weiss, 2023** | NI | Serious | Moderate | Critical | Moderate | Low | Serious | Critical |
| **Malani, 2022** | NI | Serious | Low | Serious | Serious | Low | Serious | Serious |
| **Martin-Romano, 2022** | NI | Moderate | Low | Serious | Moderate | Low | Low | Serious |
| **Miller, 2022** | NI | Serious | Low | Serious | Low | Low | Low | Serious |
| **Angel, 2021** | NI | Critical | Serious | Serious | Moderate | Low | Low | Critical |
| **Horak, 2021** | NI | Serious | Low | Serious | Moderate | Low | Low | Serious |
| **Kikuchi, 2021** | NI | Moderate | Moderate | Serious | Moderate | Moderate | Moderate | Serious |
| **Sultova, 2021** | NI | Serious | Low | Serious | Low | Low | Moderate | Serious |
| **Bitzer, 2020** | NI | Serious | Low | Serious | Serious | Low | Moderate | Serious |
| **Koopman, 2020** | NI | Serious | Low | Serious | Serious | Low | Low | Serious |
| **Sicklick, 2019** | NI | Serious | Low | Serious | Moderate | Low | Low | Serious |
| **Tredan, 2019** | NI | Low | Low | Moderate | Moderate | Low | Moderate | Moderate |
| **Trivedi, 2019** | NI | Serious | Low | Serious | Moderate | Low | Moderate | Serious |
| **Powell, 2018** | NI | Moderate | Low | Serious | Moderate | Low | Low | Serious |
| **Rodriguez-Rodriguez, 2018** | NI | Serious | Low | Serious | Serious | Moderate | Low | Serious |
| **Burkard, 2017** | NI | Serious | Low | Serious | Serious | Moderate | Moderate | Serious |
| **Massard, 2017** | NI | Serious | Low | Moderate | Low | Low | Low | Serious |
| **Kaderbhai, 2016** | NI | Serious | Serious | Serious | Serious | Low | Serious | Serious |
| **Parker, 2015** | NI | Serious | Low | Serious | Moderate | Low | Low | Serious |
| **Schwaederle, 2014** | NI | Serious | Low | Serious | Moderate | Moderate | Low | Serious |
