# Supplementary material for: Molecular Tumor Boards clinical impact on patient care and structural features: A systematic review and meta-analysis
Source: PLoS Med. 2026 Jun 9;23(6):e1005125. doi: 10.1371/journal.pmed.1005125 (PMC13274928; doi:10.1371/journal.pmed.1005125)
Supplement: S2 Appendix — Fig A. Funnel plot stratified by study design for overall survival (OS). Fig B. Funnel plot stratified by study design for progression-free survival (PFS). Fig C. Funnel plot stratified by study design for progression-free survival ratio (PFS ratio) ≥1.3. Fig D. Funnel plot stratified by study design for Objective Response Rate (ORR). Fig E. Funnel plot stratified by study design for Disease Control Rate (DCR). Fig F. Sensitivity meta-analysis of overall survival (OS) stratified by study design, including only studies in which survival time was measured from the start of treatment. HR, hazard ratio, CI, confidence interval. P-values were derived from Cochran’s Q test (for heterogeneity) and Z-tests (Wald-type tests) for pooled effects. Fig G. Sensitivity meta-analysis of progression-free survival (PFS) stratified by study design, including only studies in which survival time was measured from the start of treatment. HR, hazard ratio, CI, confidence interval. P-values were derived from Cochran’s Q test (for heterogeneity) and Z-tests (Wald-type tests) for pooled effects. Fig H. Sensitivity meta-analysis of progression-free survival ratio (PFS ratio) ≥1.3 stratified by study design, including only studies in which survival time was measured from the start of treatment. CI, confidence interval. P-values were derived from Cochran’s Q test (for heterogeneity). Fig I. Sensitivity meta-analysis of relative risk (RR) of Objective Response Rate (ORR) (panel A) and Disease Control Rate (DCR) (panel B) stratified by study design, including only studies using RECIST 1.1 criteria. CI, confidence interval; RR, relative risk. P-values were derived from Cochran’s Q test (for heterogeneity) and Z-tests (Wald-type tests) for pooled effects. Fig J. Sensitivity meta-analysis of overall survival (OS) stratified by study design, excluding studies with hazard ratios (HRs) reconstructed from Kaplan–Meier curves. HR, hazard ratio; CI, confidence interval. P-values were derived from Cochran’ [file pmed.1005125.s003.docx]

**Fig A.** Funnel plot stratified by study design for overall survival (OS)

| RCT  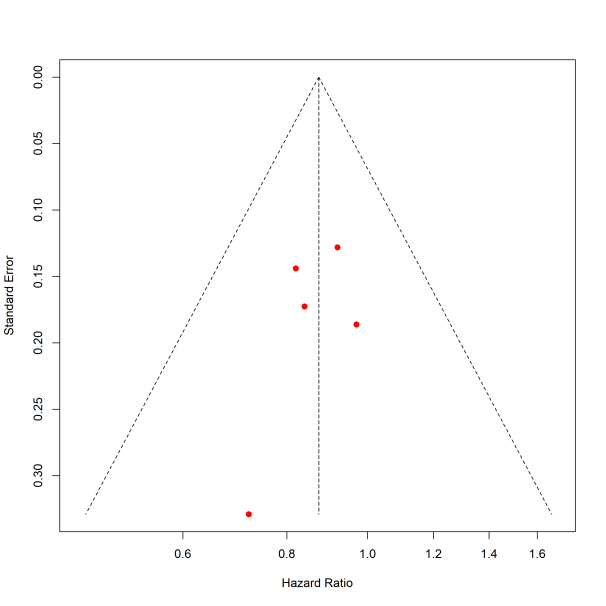 | Non-Randomized Clinical Trial 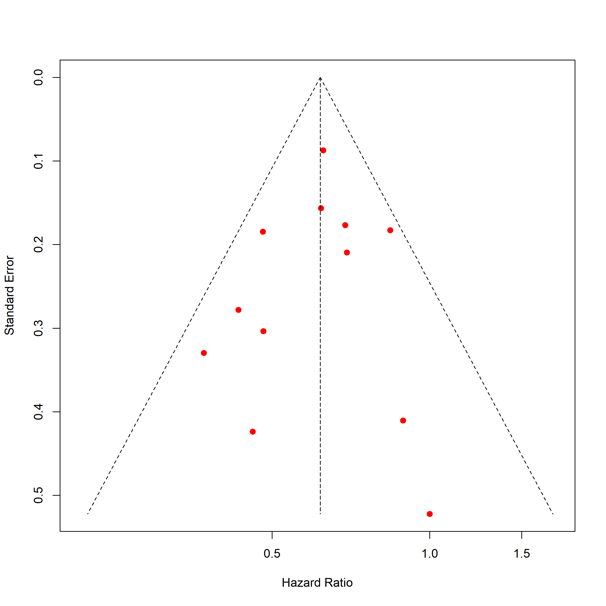 |
| --- | --- |
| Observational Prospective  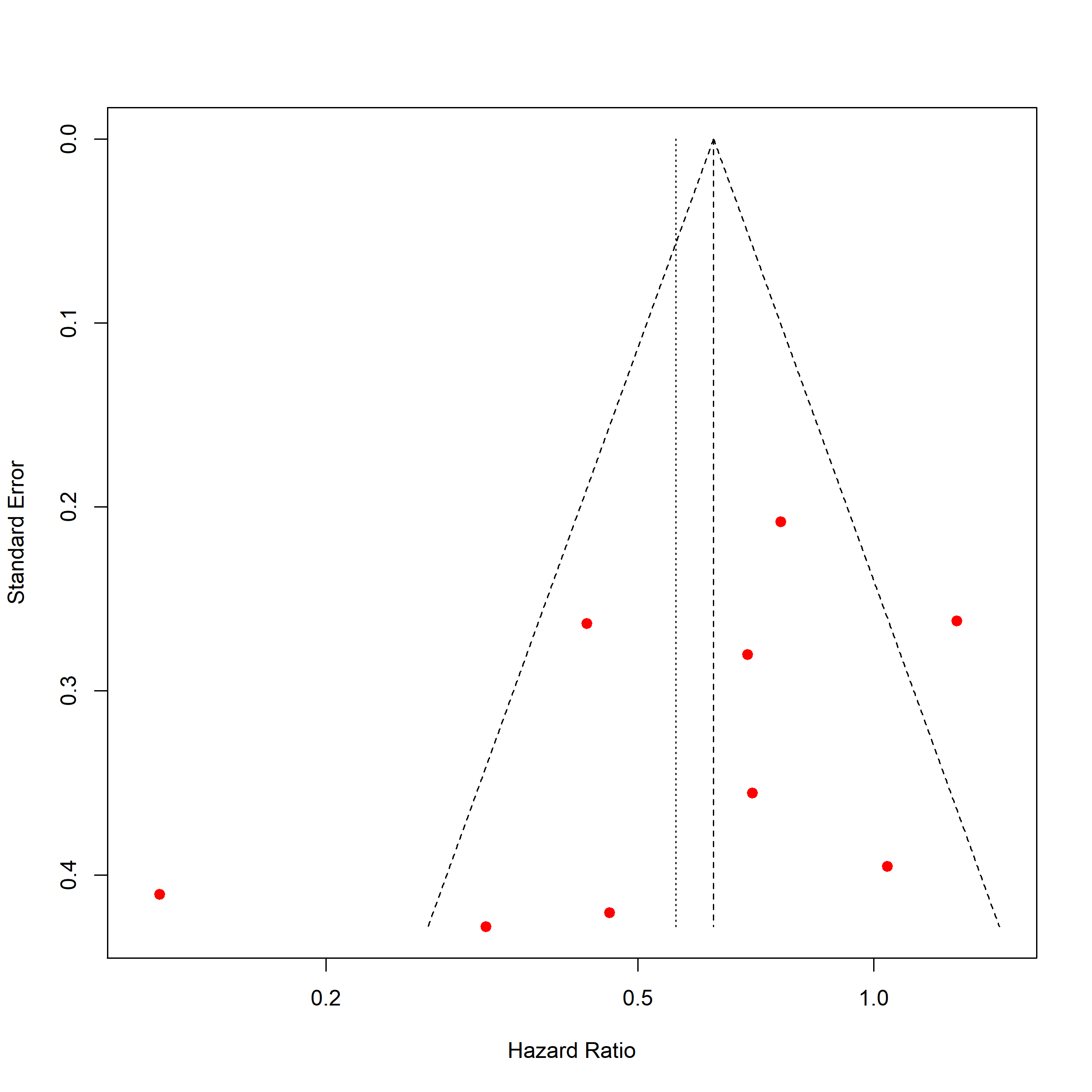 | Observational Retrospective  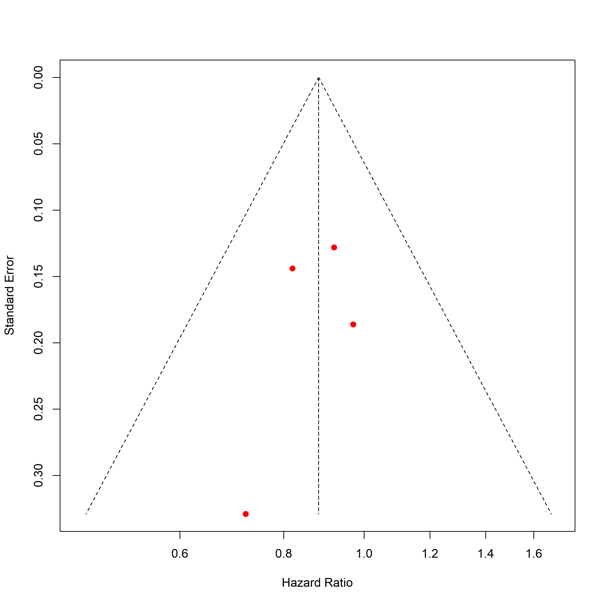 |

**Fig B.** Funnel plot stratified by study design for progression free survival (PFS)

| RCT  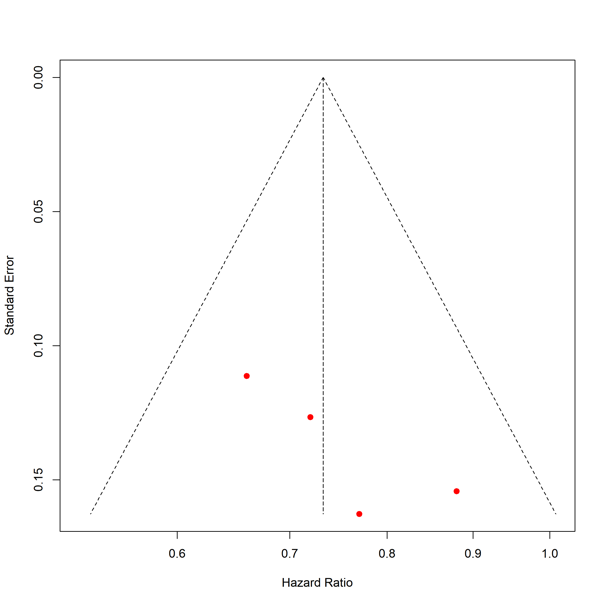 | Non-Randomized Clinical Trial 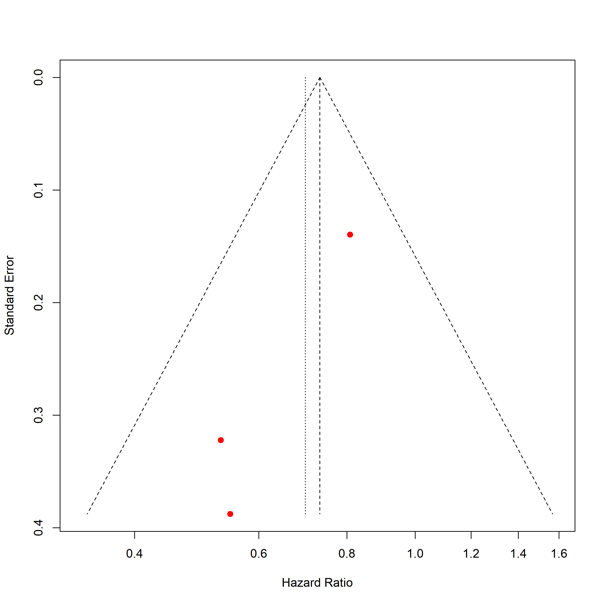 |
| --- | --- |
| Observational Prospective  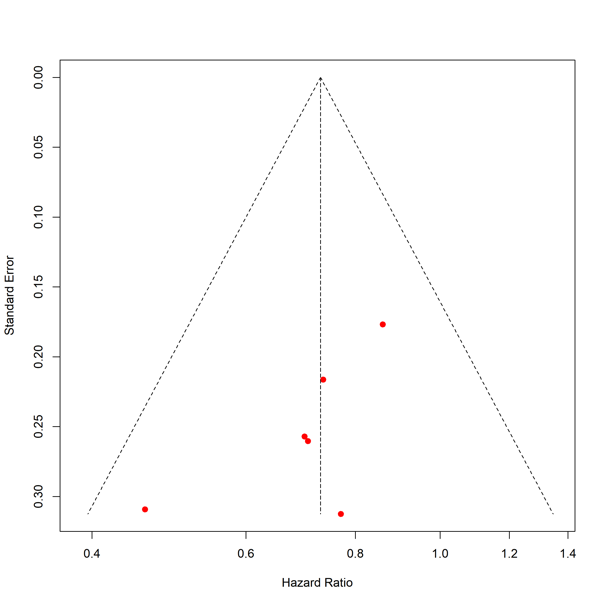 | Observational Retrospective  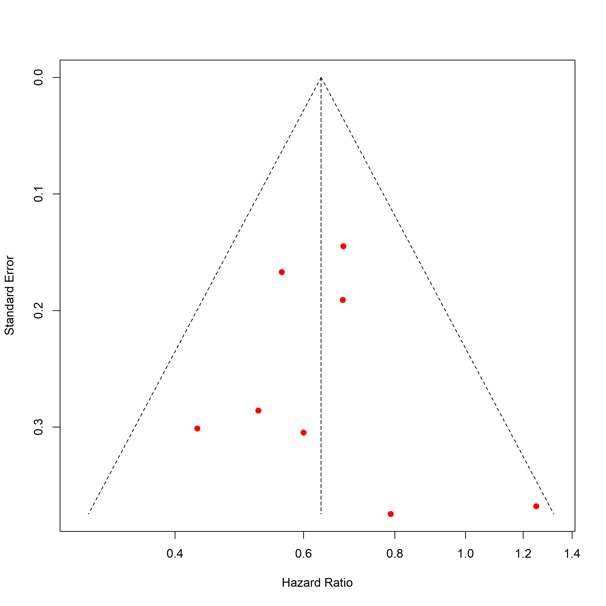 |

**Fig C.** Funnel plot stratified by study design for Progression Free Survival ratio (PFS ratio) ≥1.3.

| RCT | Non-Randomized Clinical Trial  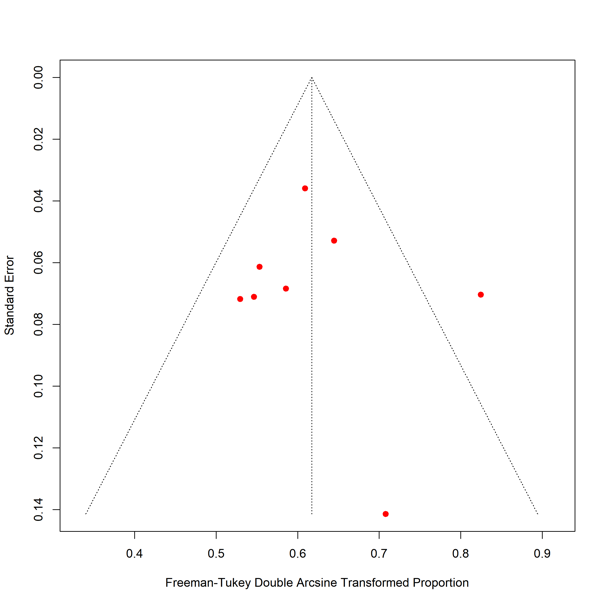 |
| --- | --- |
| Observational Prospective  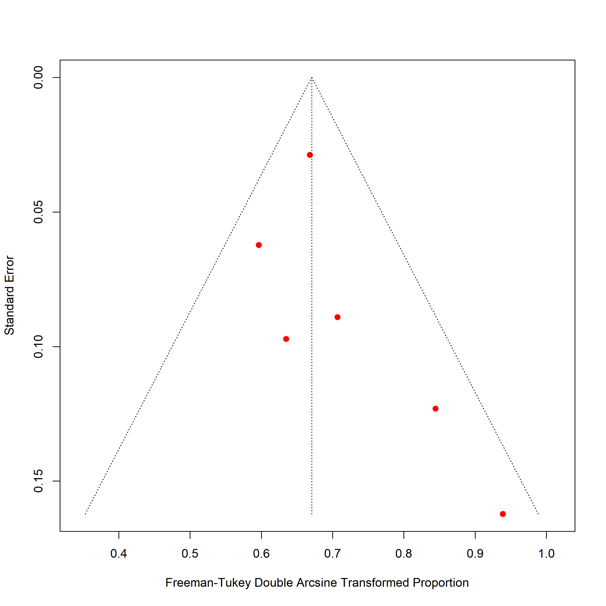 | Observational Retrospective  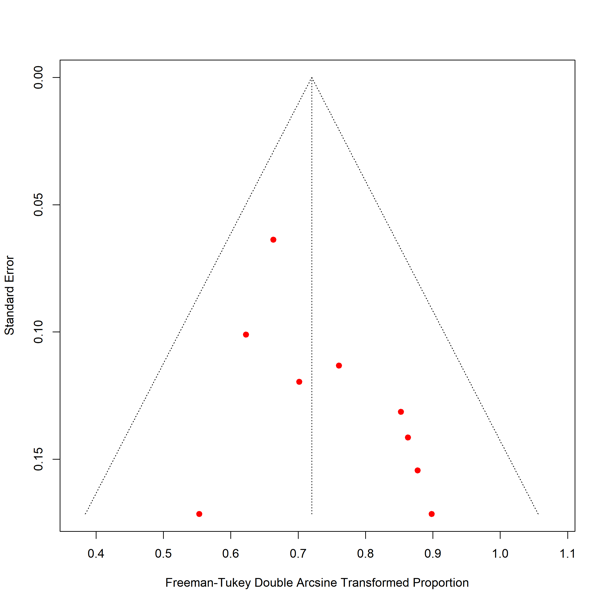 |

**Fig D.** Funnel plot stratified by study design for Objective Response Rate (ORR)

| RCT  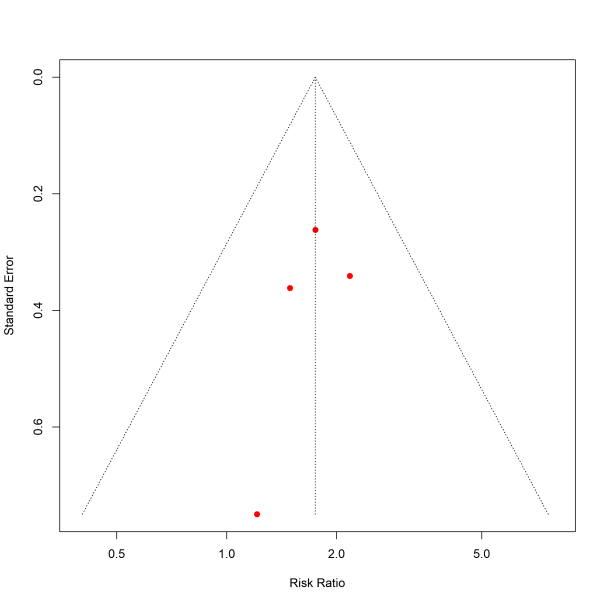 | Non-Randomized Clinical Trial  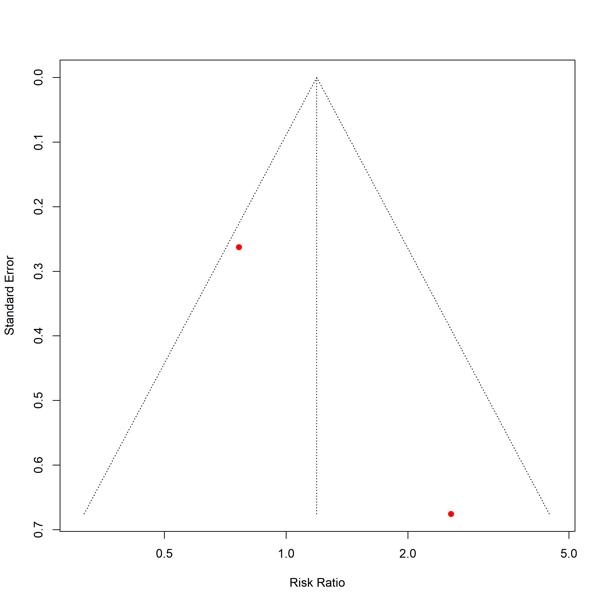 |
| --- | --- |
| Observational Prospective  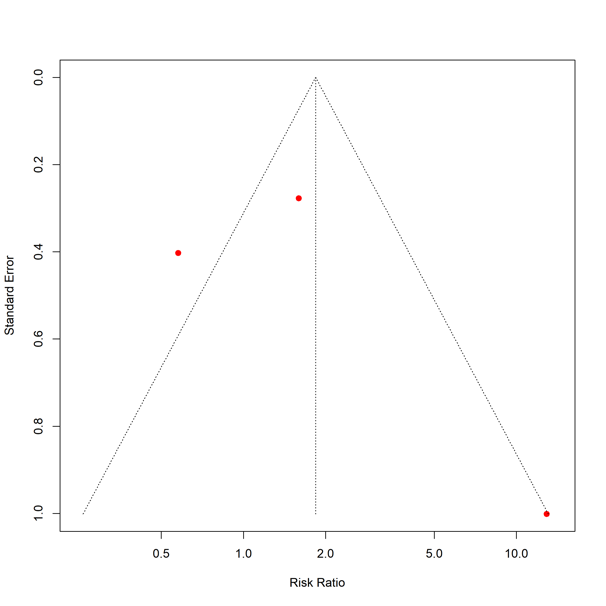 | Observational Retrospective  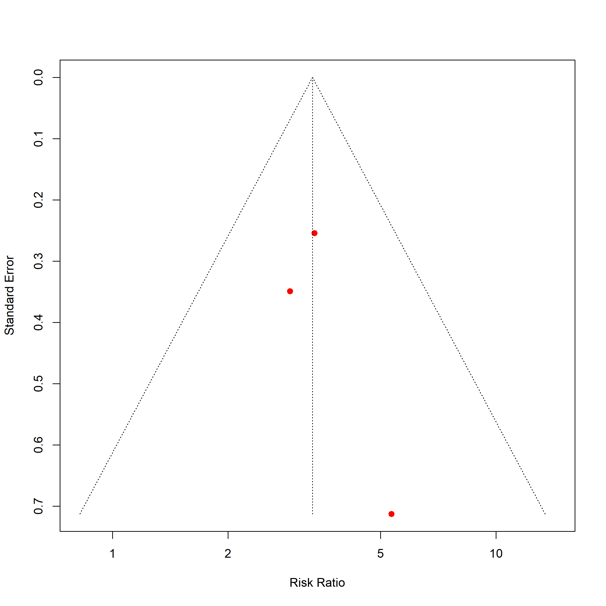 |

**Fig E.** Funnel plot stratified by study design for Disease Control Rate (DCR)

| RCT  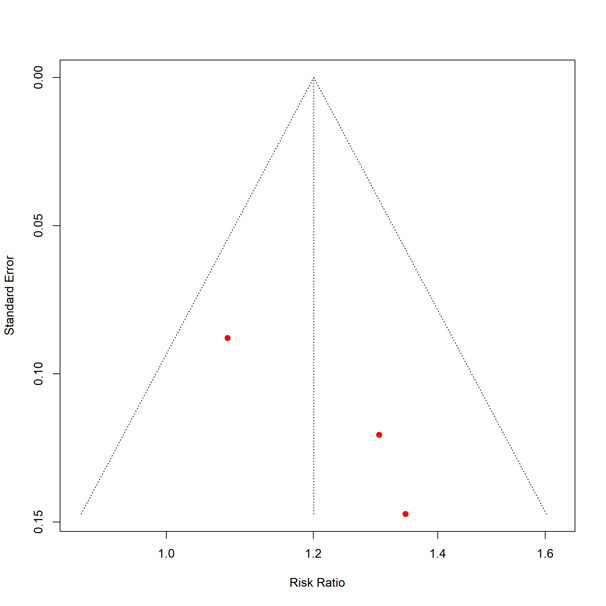 | Non-Randomized Clinical Trial  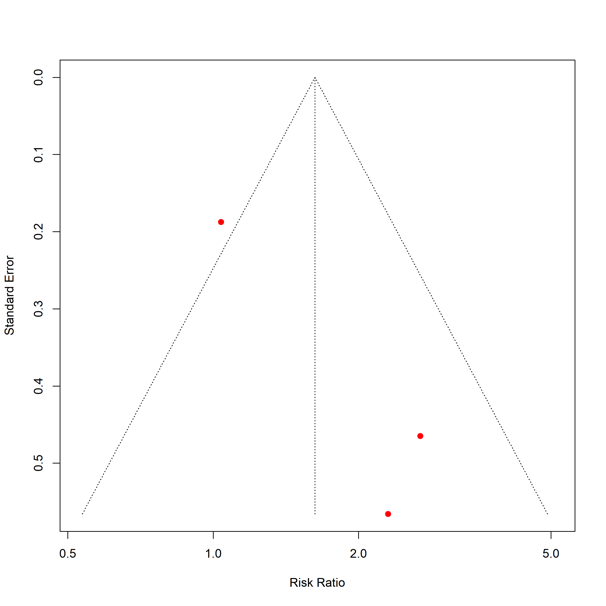 |
| --- | --- |
| Observational Prospective  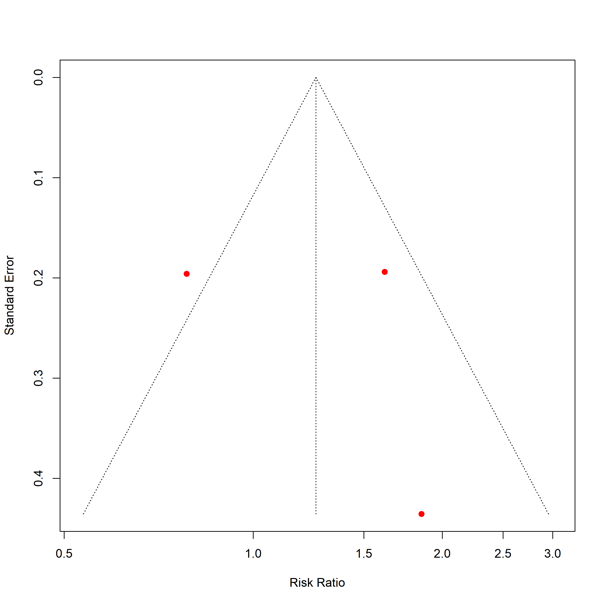 | Observational Retrospective  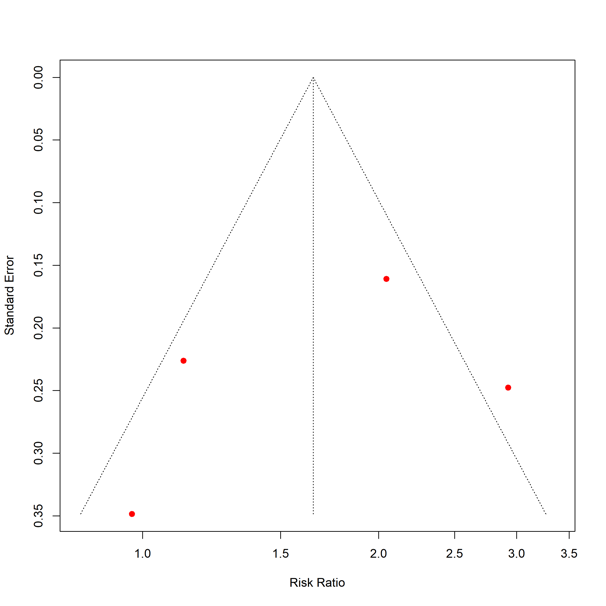 |

**Fig F.** Sensitivity meta-analysis of overall survival (OS) stratified by study design, including only studies in which survival time was measured from the start of treatment.


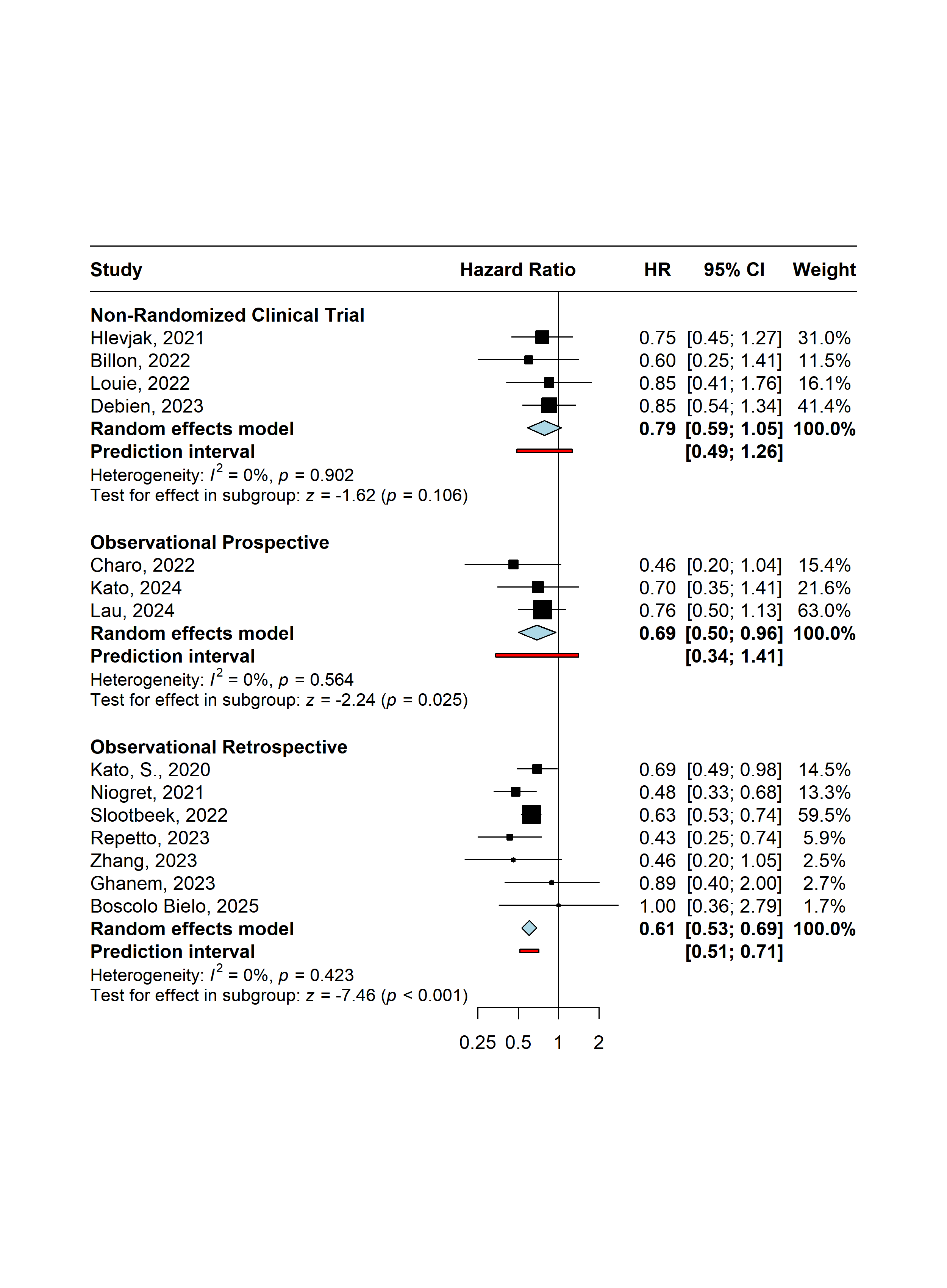


HR = hazard ratio, CI = confidence interval. P-values were derived from Cochran’s Q test (for heterogeneity) and Z-tests (Wald-type tests) for pooled effects.

**Fig G.** Sensitivity meta-analysis of progression free survival (PFS) stratified by study design, including only studies in which survival time was measured from the start of treatment.
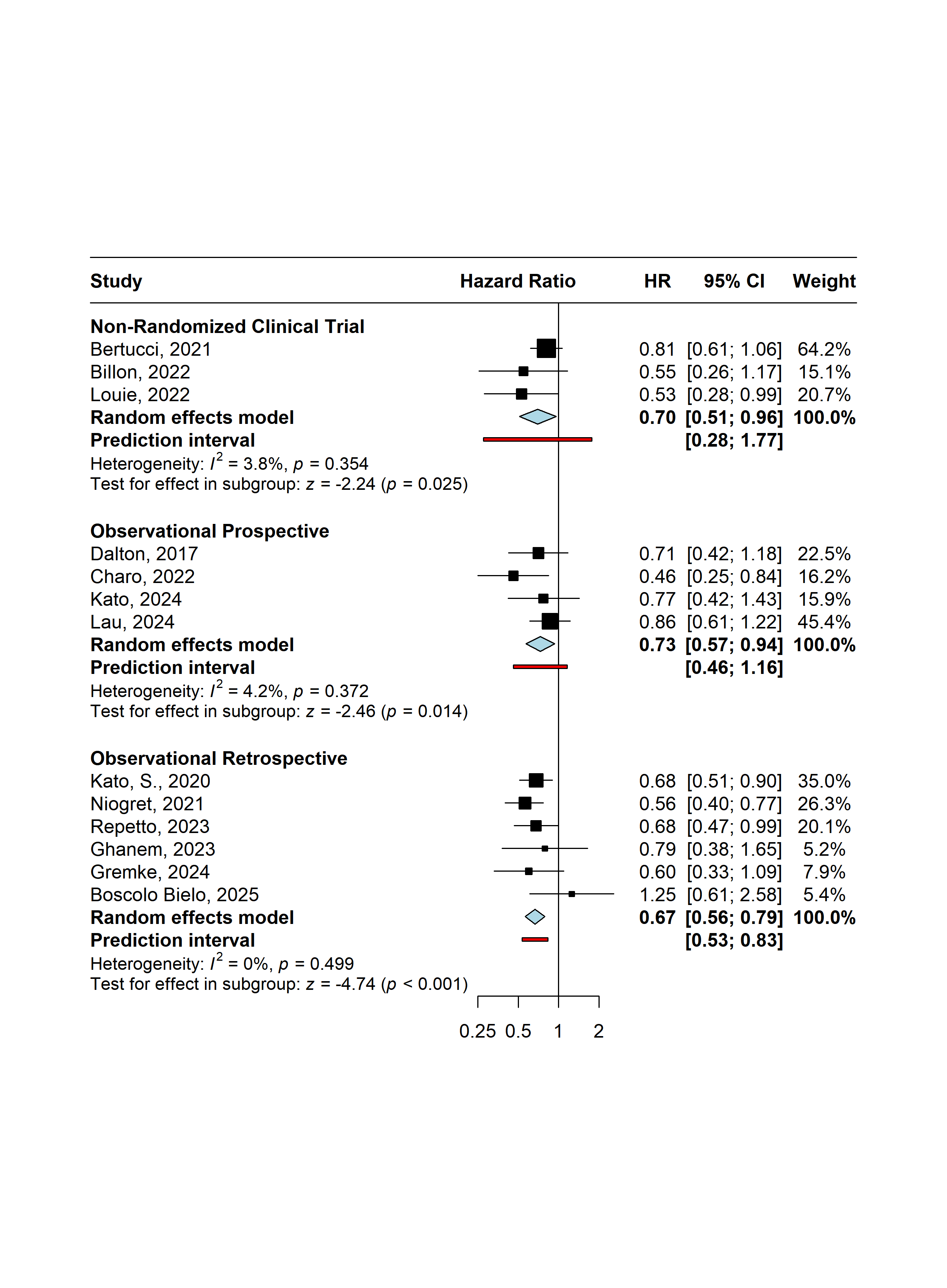


HR = hazard ratio, CI = confidence interval. P-values were derived from Cochran’s Q test (for heterogeneity) and Z-tests (Wald-type tests) for pooled effects.

**Fig H.** Sensitivity meta-analysis of Progression Free Survival ratio (PFS ratio) ≥ 1.3 stratified by study design, including only studies in which survival time was measured from the start of treatment.


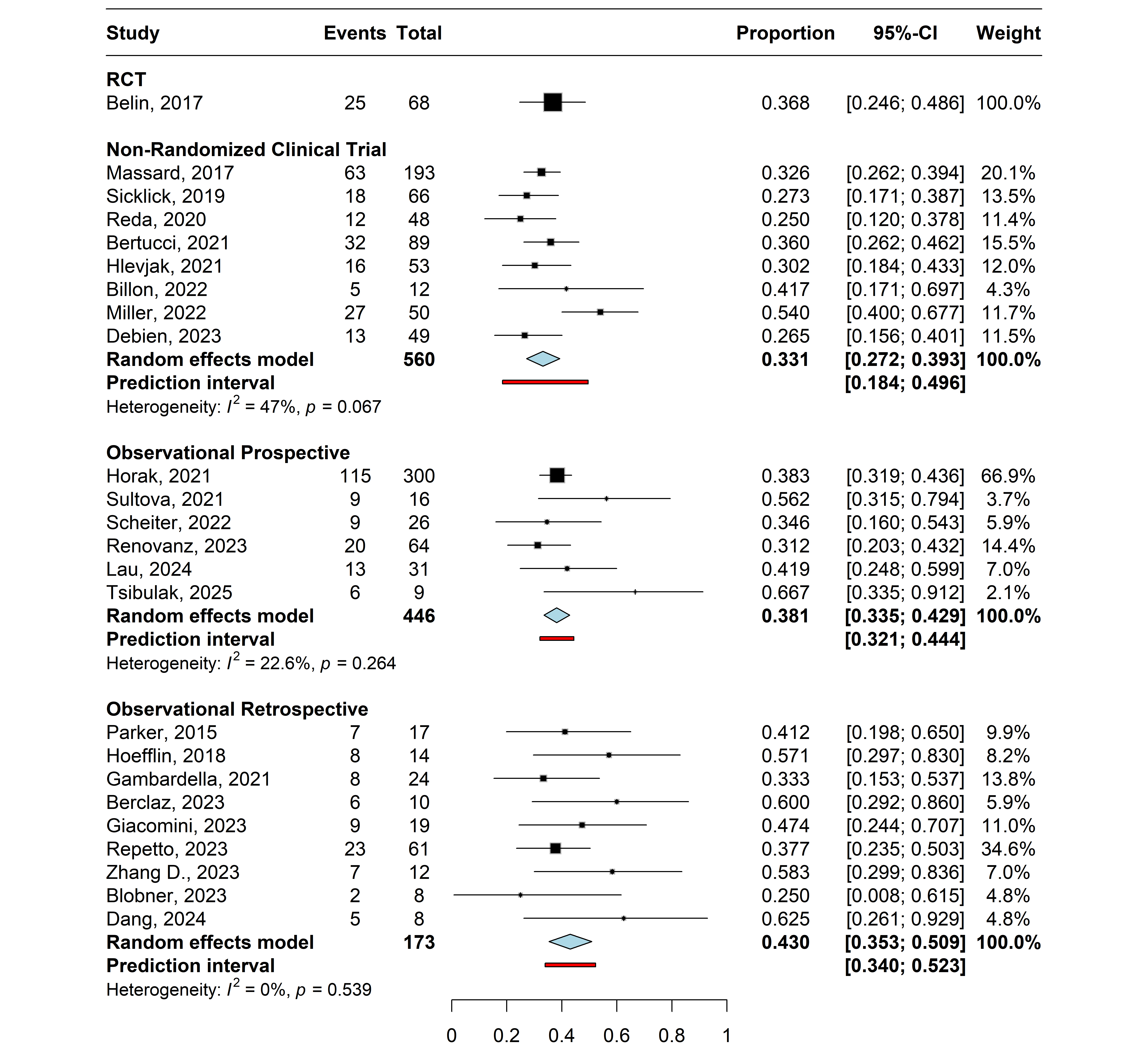


CI = confidence interval. P-values were derived from Cochran’s Q test (for heterogeneity).

**Fig I.** Sensitivity meta-analysis of Relative Risk (RR) of Objective Response Rate (ORR) (panel A) and Disease Control Rate (DCR) (panel B) stratified by study design, including only studies using RECIST 1.1 criteria.

| **A)**  **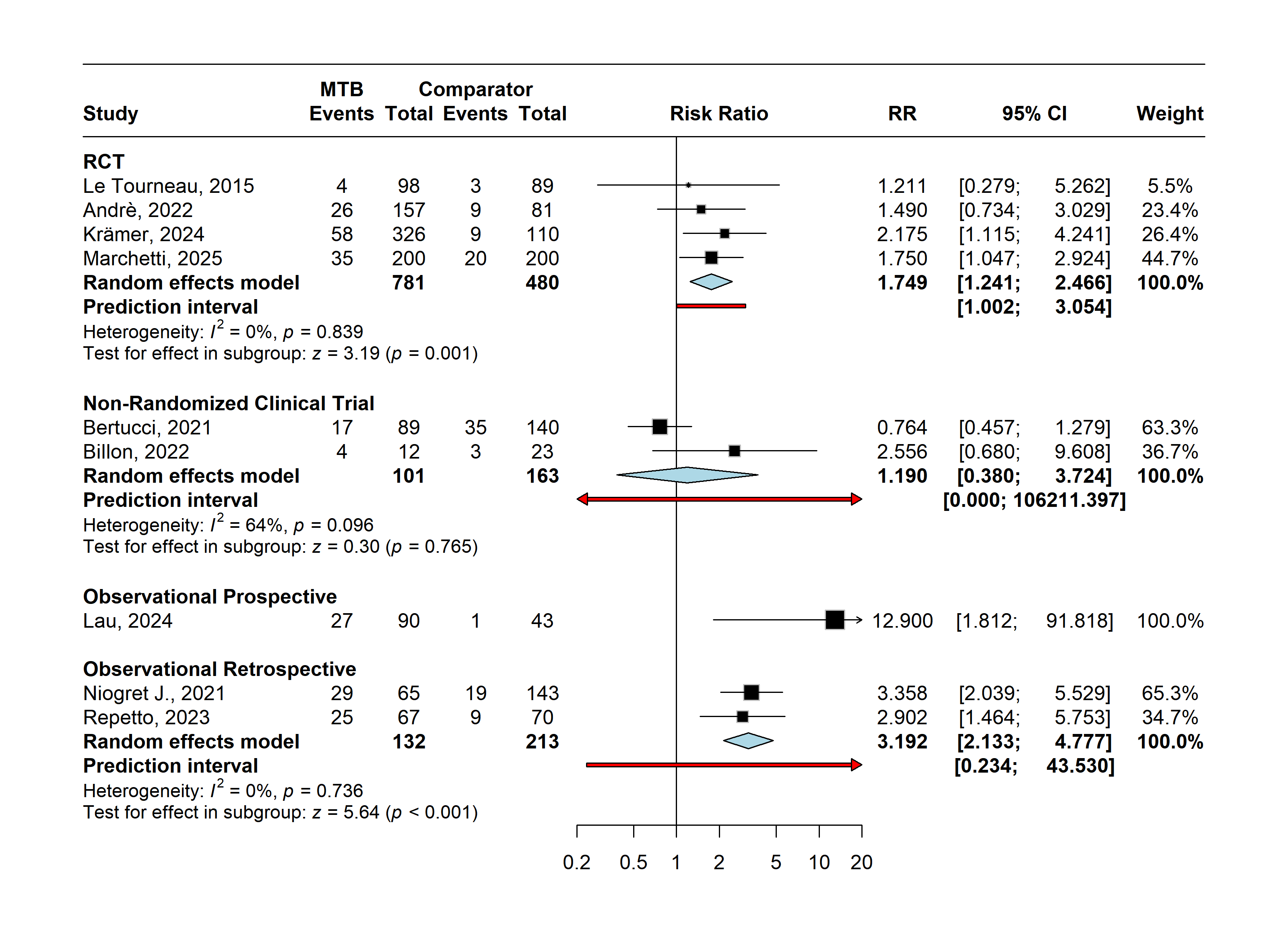** |
| --- |
| **B)**  **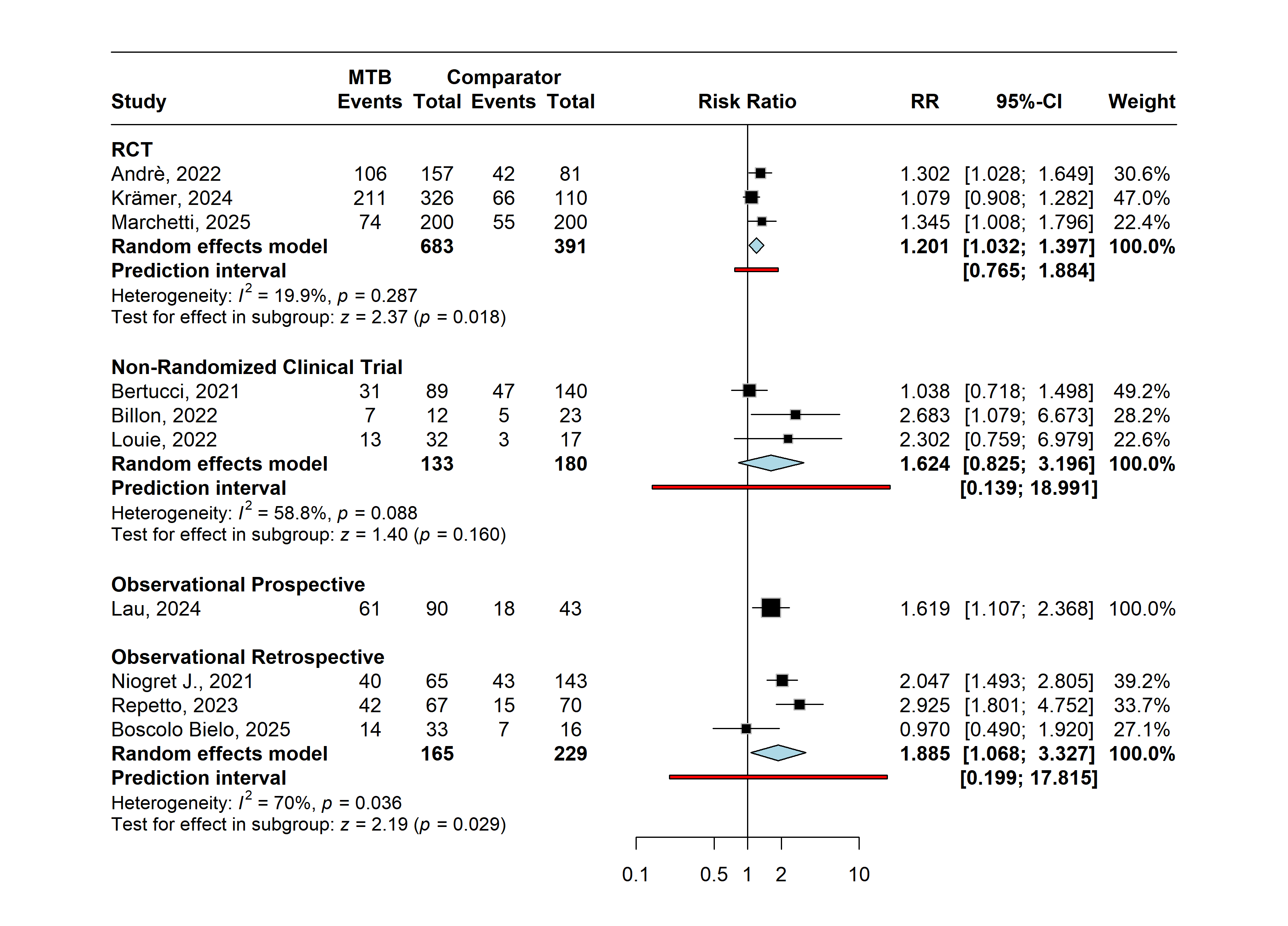** |
| CI = confidence interval, RR = Relative Risk. P-values were derived from Cochran’s Q test (for heterogeneity) and Z-tests (Wald-type tests) for pooled effects. |

**Fig J.** Sensitivity meta-analysis of overall survival (OS) stratified by study design, excluding studies with hazard ratios (HRs) reconstructed from Kaplan-Meier curves.


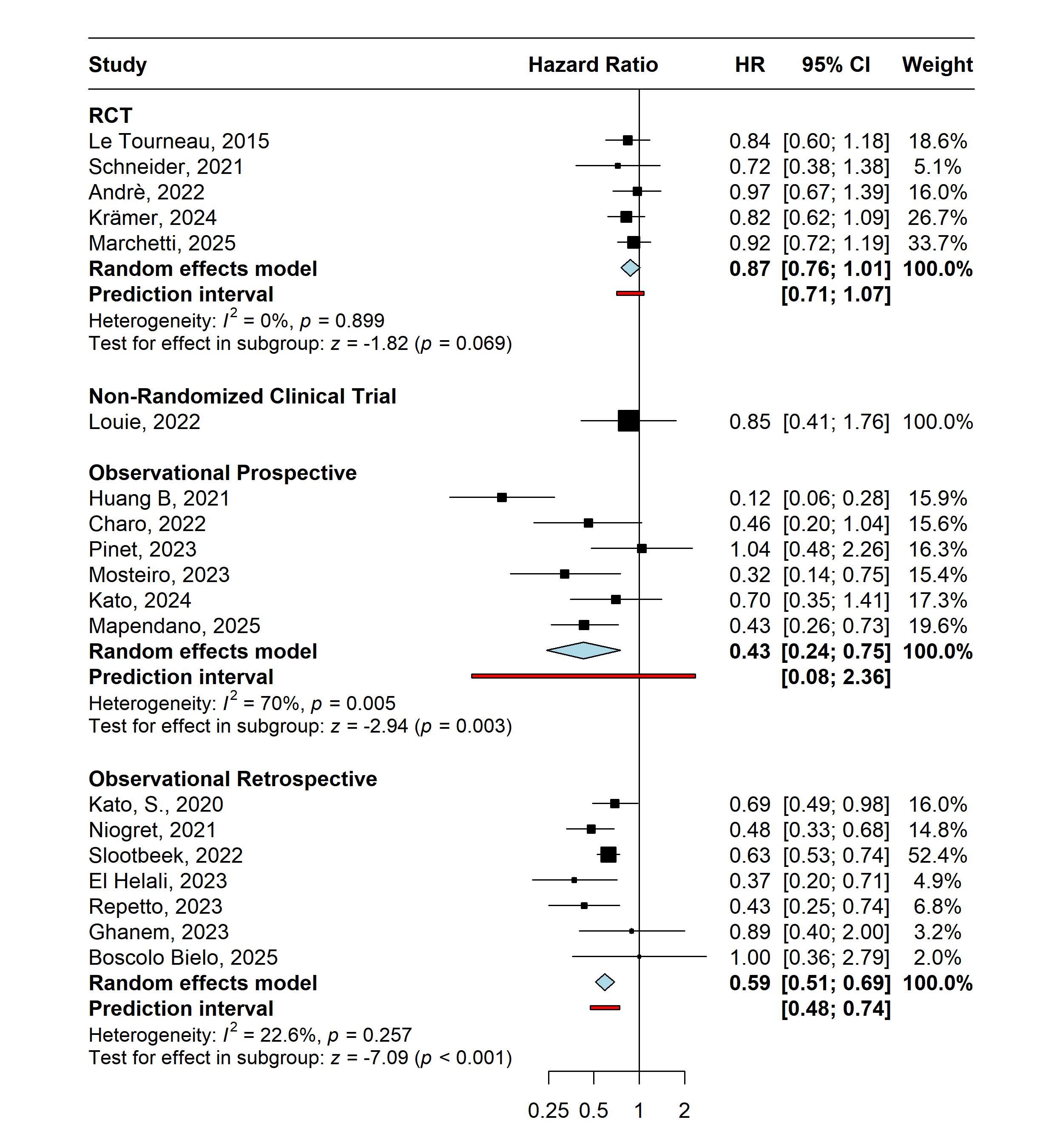


HR = hazard ratio, CI = confidence interval. P-values were derived from Cochran’s Q test (for heterogeneity) and Z-tests (Wald-type tests) for pooled effects.

**Fig K.** Sensitivity meta-analysis of progression free survival (PFS) stratified by study design, excluding studies with hazard ratios (HRs) reconstructed from Kaplan-Meier curves.


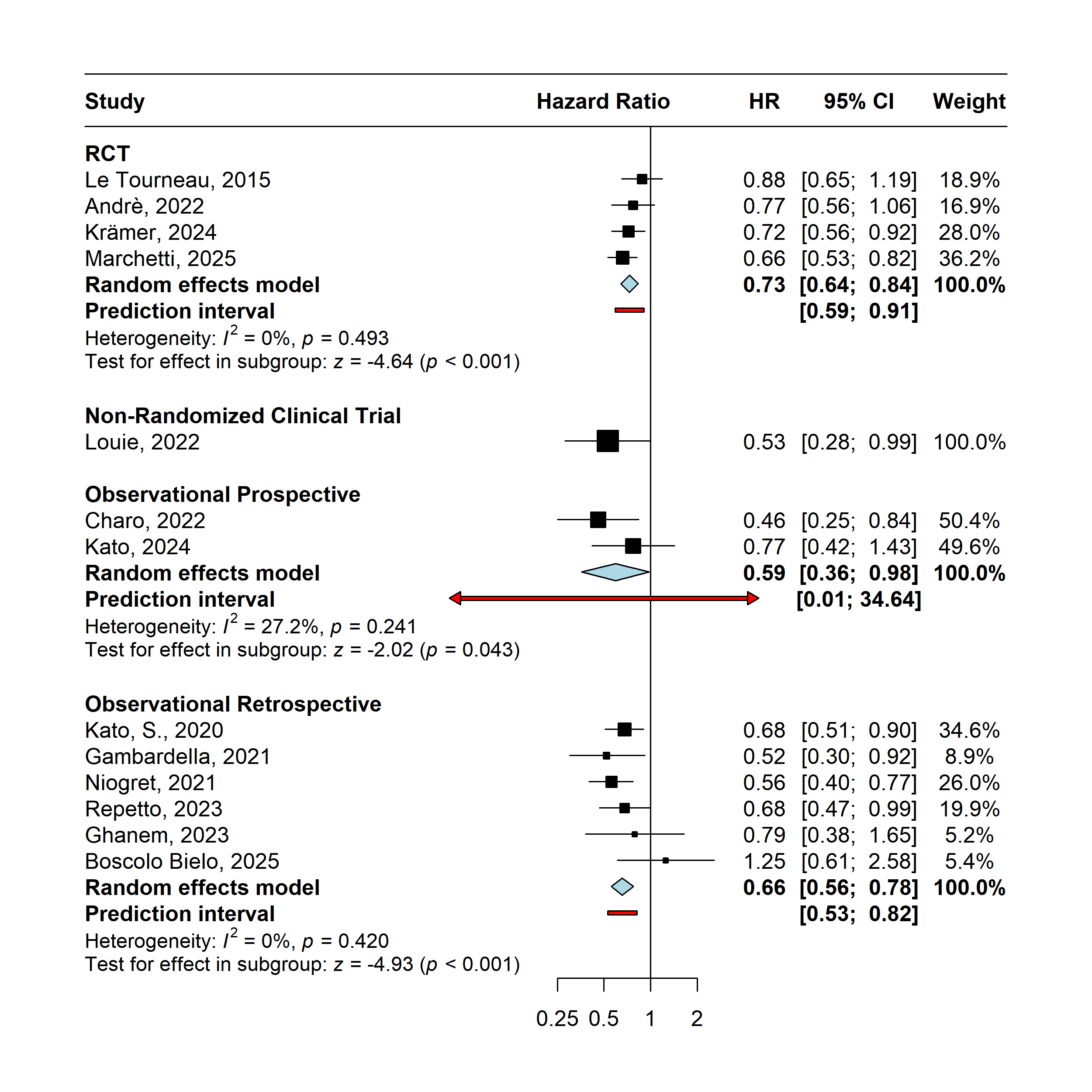


HR = hazard ratio, CI = confidence interval. P-values were derived from Cochran’s Q test (for heterogeneity) and Z-tests (Wald-type tests) for pooled effects.

**Fig L.** Risk of bias for Randomized Controlled Trials (RCTs) using RoB 2.


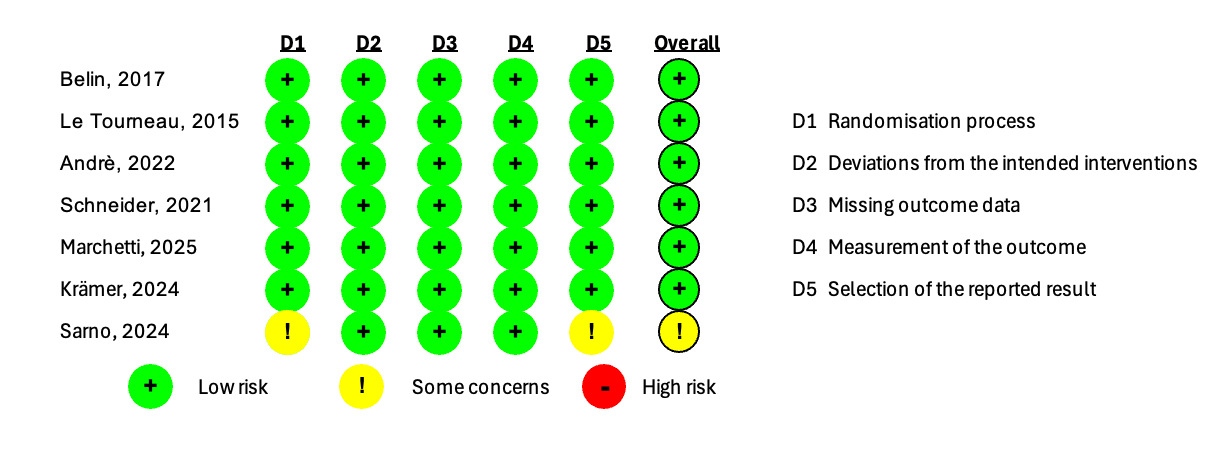


**Fig M.** Sensitivity meta-analysis of overall survival (OS) stratified by study design, excluding studies with serious or critical risk of bias.


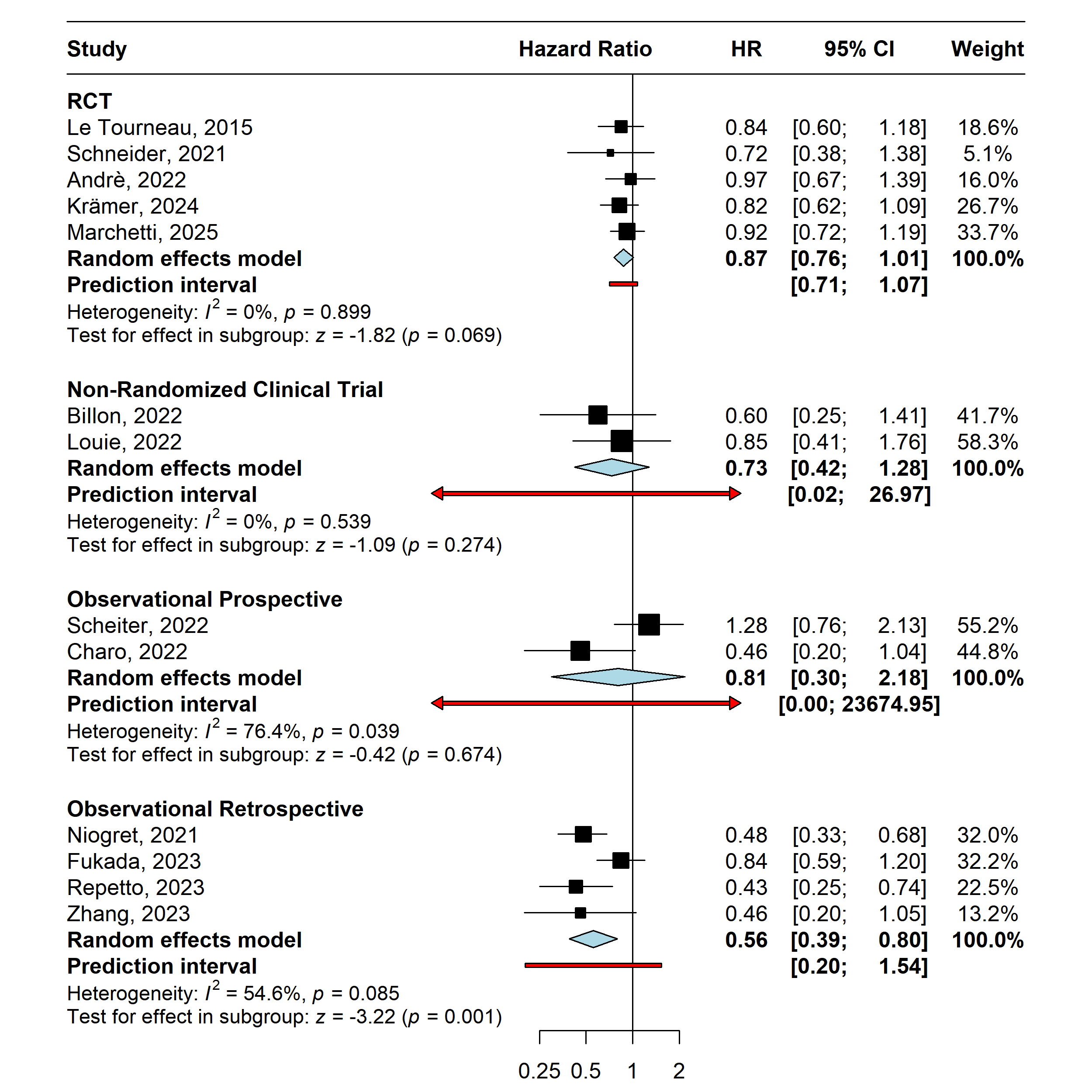


HR = hazard ratio, CI = confidence interval. P-values were derived from Cochran’s Q test (for heterogeneity) and Z-tests (Wald-type tests) for pooled effects.

**Fig N.** Sensitivity meta-analysis of progression free survival (PFS) stratified by study design, excluding studies with serious or critical risk of bias.
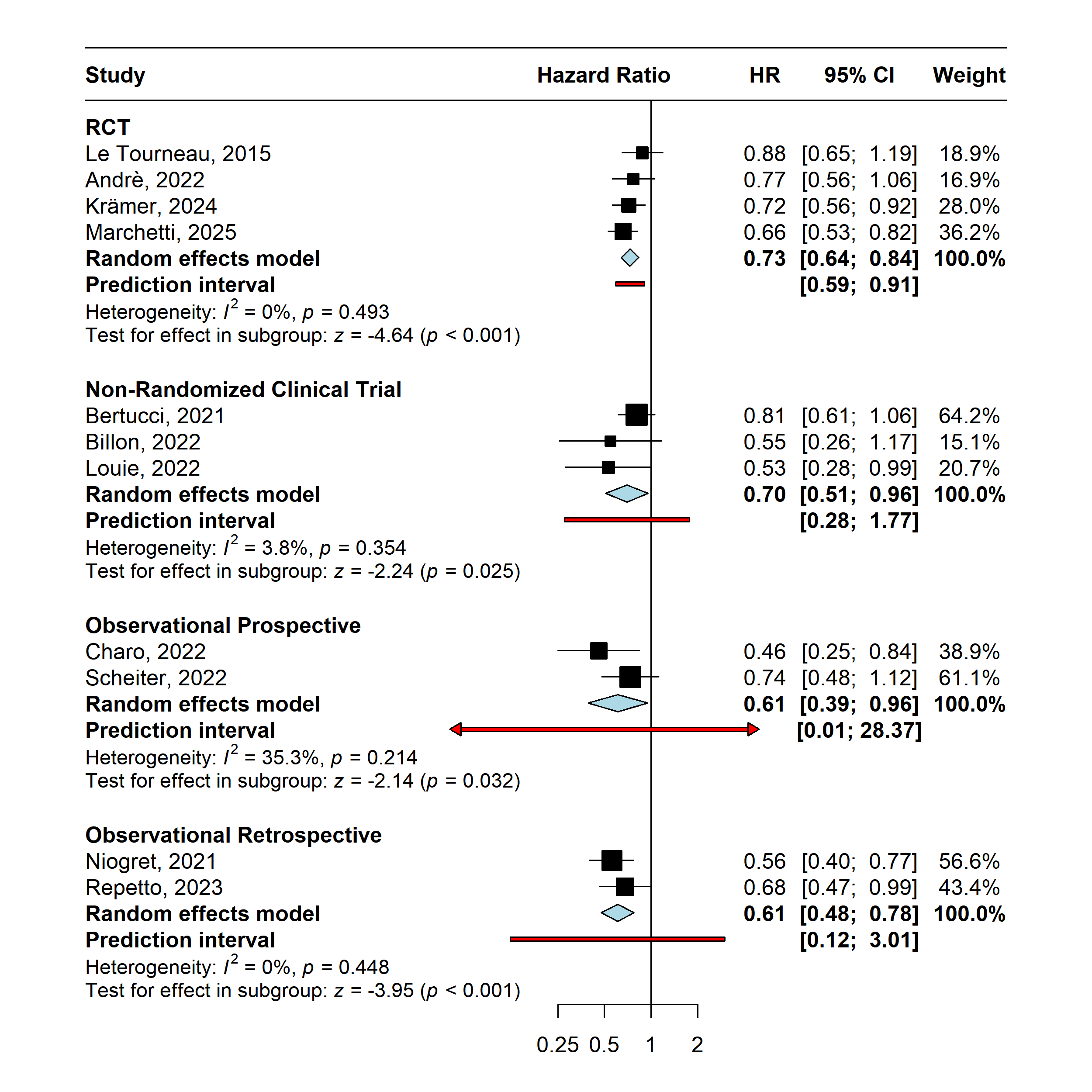


HR = hazard ratio, CI = confidence interval. P-values were derived from Cochran’s Q test (for heterogeneity) and Z-tests (Wald-type tests) for pooled effects.

**Fig O.** Sensitivity meta-analysis of Progression Free Survival ratio (PFS ratio) ≥ 1.3 stratified by study design, excluding studies with serious or critical risk of bias.
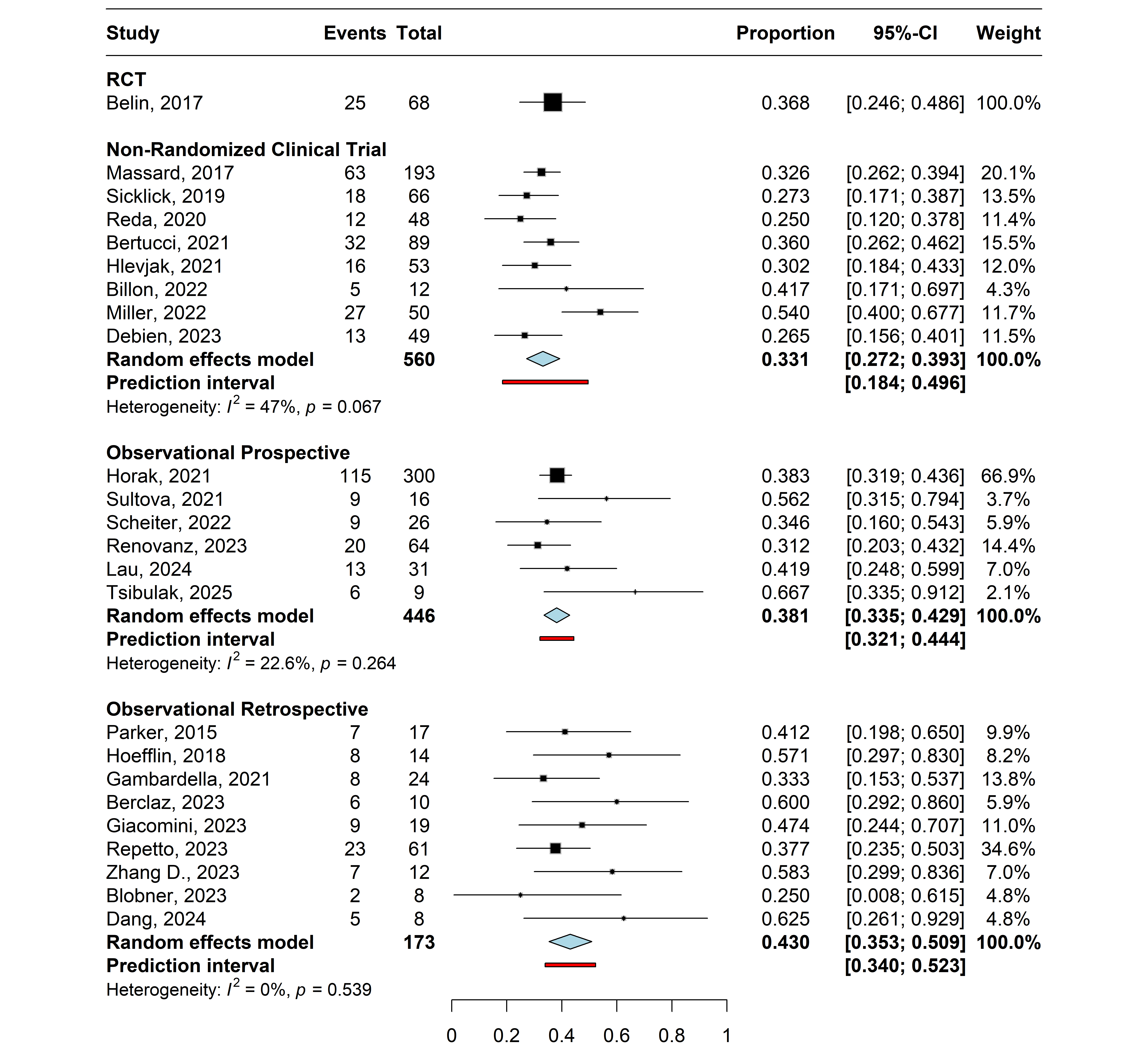


CI = confidence interval. P-values were derived from Cochran’s Q test (for heterogeneity).

**Fig P.** Sensitivity meta-analysis of Relative Risk (RR) Objective Response Rate (ORR) (panel A) and Disease Control Rate (DCR) (panel B) stratified by study design, excluding studies with serious or critical risk of bias.

| **A)**  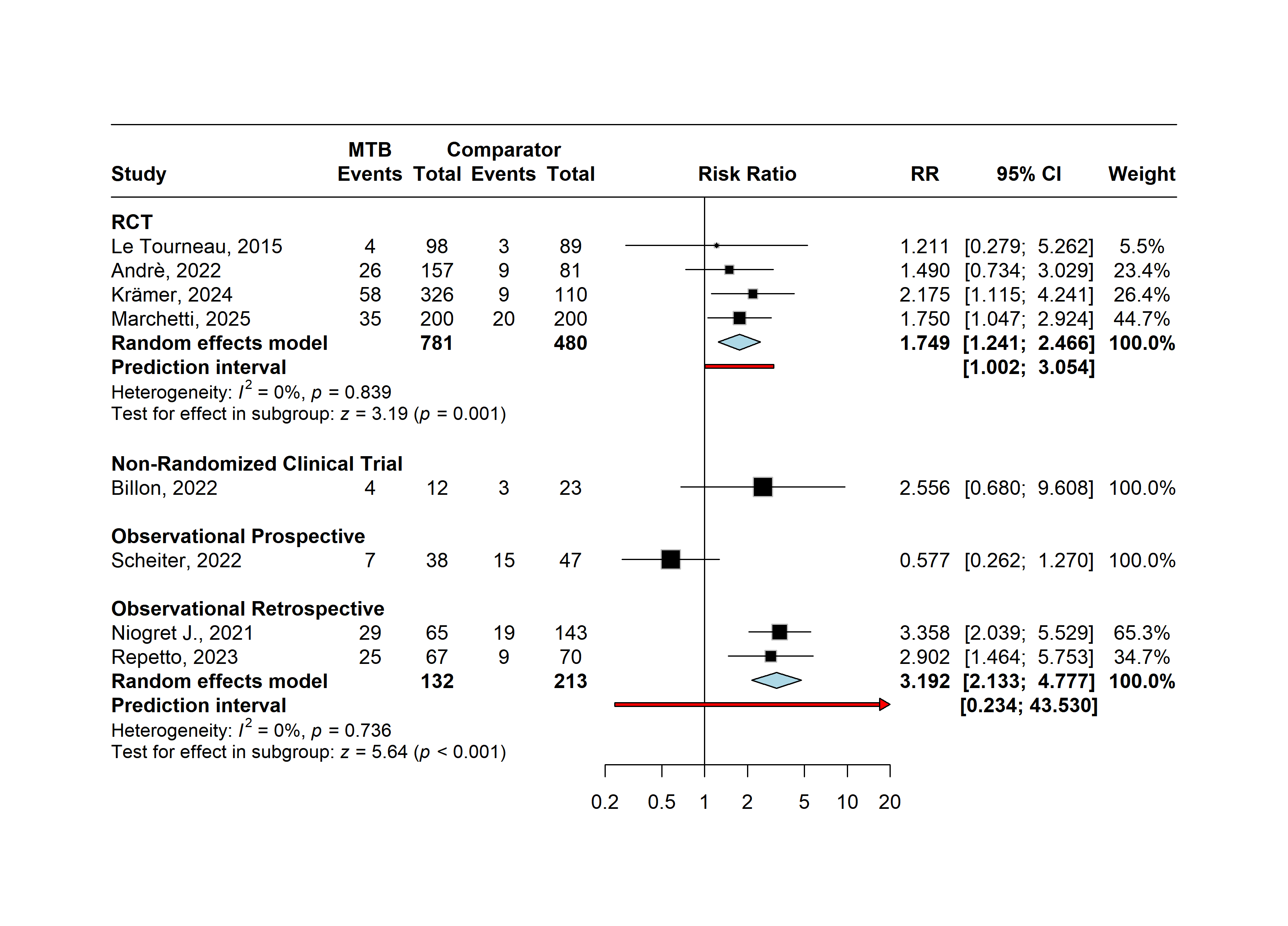 |
| --- |
| **B)**  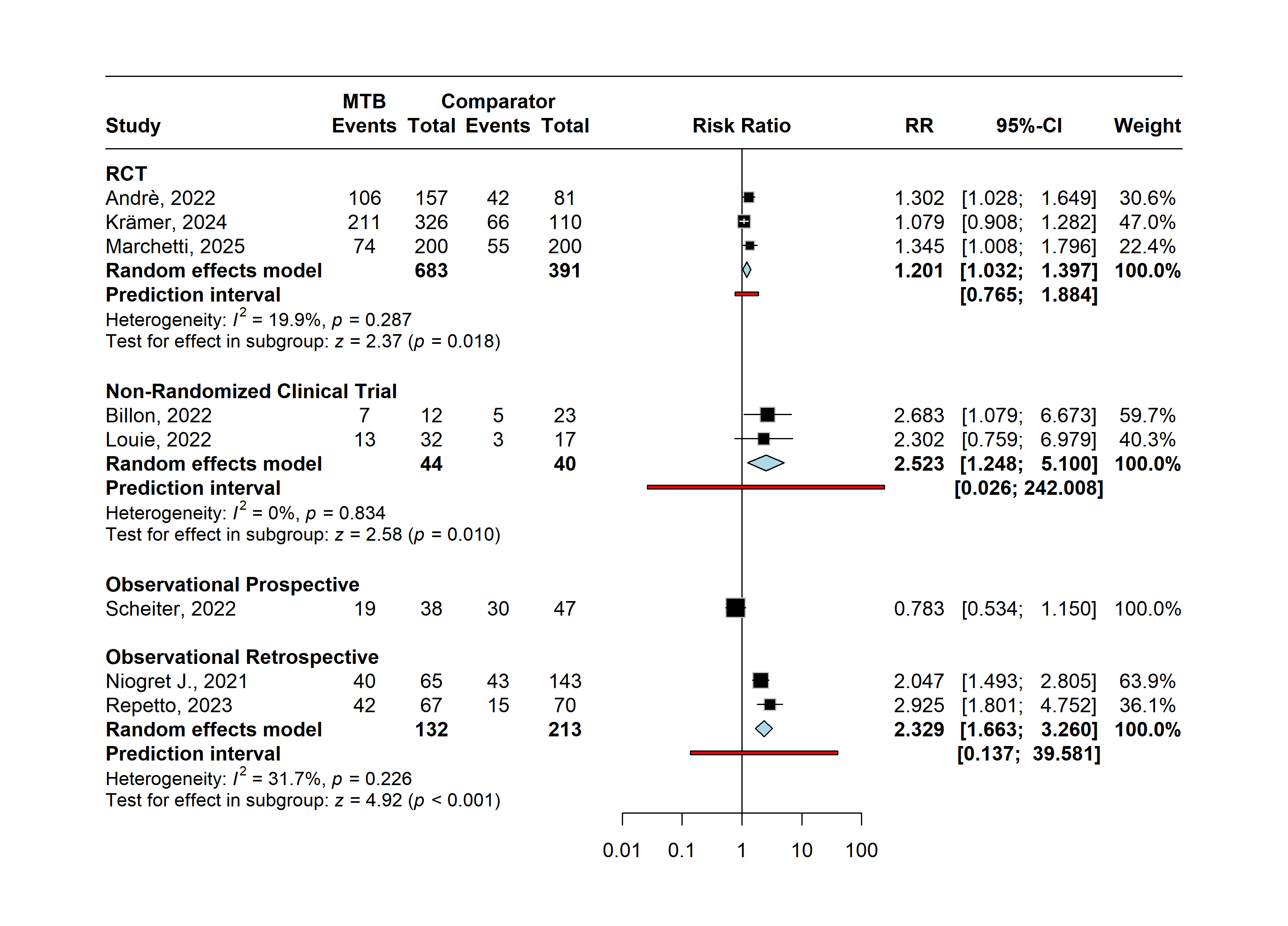 |

CI = confidence interval, RR = Relative Risk. P-values were derived from Cochran’s Q test (for heterogeneity) and Z-tests (Wald-type tests) for pooled effects.
