# Supplementary material for: Molecular Tumor Boards clinical impact on patient care and structural features: A systematic review and meta-analysis
Source: PLoS Med. 2026 Jun 9;23(6):e1005125. doi: 10.1371/journal.pmed.1005125 (PMC13274928; doi:10.1371/journal.pmed.1005125)
Supplement: S1 File — (PDF) [file pmed.1005125.s004.pdf]

**Supplementary File 1.** Excluded records at full text screening (n= 114).

| Authors | Year | Title | Source/ | Meets inclusion criteria? | Exclusion reason |
|---|---|---|---|---|---|
| Parker, BA et al. | 2013 | UC San Diego molecular tumor board: Experience in breast cancer | CANCER RESEARCH | No | Abstract or Poster |
| NCT02162732 | 2014 | Molecular-Guided Therapy for Childhood Cancer | ClinicalTrials.gov | No | Same population already included |
|  | 2015 | The impact of a molecular tumor board on treatment decisions for 35 patients: The Dartmouth experience | JOURNAL OF CLINICAL ONCOLOGY | No | Abstract or Poster |
| Tafe, LJ et al. | 2015 | Implementation of a molecular tumor board: The impact on treatment decisions for 35 patients evaluated at Dartmouth-Hitchcock medical center | Oncologist | No | No measurable clinical outcomes |
| Tafe, LJ et al. | 2015 | Implementation of a Molecular Tumor Board: The Impact on Treatment Decisions for NSCLC Patients Evaluated at Dartmouth-Hitchcock in One Year | Oncologist | No | No measurable clinical outcomes |
| Armeanu-Ebinger, S et al. | 2016 | Implementation of an interdisciplinary molecular tumor board in managing of advanced stage breast cancer | ANNALS OF ONCOLOGY | No | Abstract or Poster |
| Bernhardt, E et al. | 2016 | Implementation of a Molecular Tumor Board at Dartmouth-Hitchcock Medical Center: the impact on treatment decisions over a two year period | Journal of Clinical Oncology | No | Abstract or Poster |
| Hirshfield, KH et al. | 2016 | Clinical Actionability of Comprehensive Genomic Profiling for Management of Rare or Refractory Cancers | ONCOLOGIST | No | No measurable clinical outcomes |

| | | | | | |
|---|---|---|---|---|---|
| Siniard, RC et al. | 2016 | The Molecular Tumor Board: A Two Year Experience at the University of Alabama at Birmingham | JOURNAL OF MOLECULAR DIAGNOSTICS | No | Abstract or Poster |
| | 2017 | Initial experience with a virtual molecular tumor board in a pragmatic precision medicine study | TRIALS | No | No full text available |
| Marks, LJ et al. | 2017 | Precision medicine in children and young adults with hematologic malignancies and blood disorders: The Columbia university experience | Frontiers in Pediatrics | No | No measurable clinical outcomes |
| Pagès, A et al. | 2017 | The cost of molecular-guided therapy in oncology: A prospective cost study alongside the MOSCATO trial | Genetics in Medicine | No | No measurable clinical outcomes |
| Parsons, HA et al. | 2017 | Individualized Molecular Analyses Guide Efforts (IMAGE): A Prospective Study of Molecular Profiling of Tissue and Blood in Metastatic Triple-Negative Breast Cancer | CLINICAL CANCER RESEARCH | No | No measurable clinical outcomes |
| | 2017 | PROFILER 02 - A multicentric, prospective cohort study aiming to evaluate the added value of a large molecular profiling panel (315 cancer-related gene panel [FoundationOne]) versus a limited molecular profiling panel (74 cancer-related gene panel [CONTROL]) in advanced solid tumours | COCHRANE | No | Same population already included |
| Elst, AT et al. | 2017 | Treatment decision-making of rare <i>ERBB2</i> (HER2) mutations in lung cancer | Cancer Research | No | Abstract or Poster |
| NCT03546127 | 2017 | Molecular Profiling to Improve Outcome of Patients in Cancer. A Pilot Study | ClinicalTrials.gov | No | No full text available |

| | | | | | |
|---|---|---|---|---|---|
| NCT03929653 | 2017 | Personalized Therapy of Molecular Tumor Board Participation With the Guidance of Next Generation Sequencing | ClinicalTrials.gov | No | No full text available |
| | 2018 | GENETIC SEQUENCING FOR THE TREATMENT OF ADVANCED SOFT-TISSUE SARCOMAS | | No | Same population already included |
| Besse, C et al. | 2018 | Relevance of a molecular tumour board (MTB) for patients' enrolment in clinical trials: experience of the Institut Curie | ESMO OPEN | No | No measurable clinical outcomes |
| Angel, MO et al. | 2018 | Implementation of a Molecular Tumor Board: one year experience in a routine setting | ONCOLOGY RESEARCH AND TREATMENT | No | No full text available |
| Chu, L et al. | 2018 | Outcomes of Patients with Metastatic Lung Cancer Presented in a Multidisciplinary Molecular Tumor Board | JOURNAL OF THORACIC ONCOLOGY | No | Abstract or Poster |
| Rolfo, CD et al. | 2018 | Effects of molecular tumor board and different NGS panels implementation for the treatment of patients with cancer | JOURNAL OF CLINICAL ONCOLOGY | No | Abstract or Poster |
| Rolfo, R et al. | 2018 | Integrating Liquid Biopsy in Molecular Tumor Board Decision for Lung Cancer Patients | JOURNAL OF THORACIC ONCOLOGY | No | Wrong study design |
| Rolfo, R et al. | 2018 | Multidisciplinary molecular tumour board: A tool to improve clinical practice and selection accrual for clinical trials in patients with cancer | ESMO Open | No | No measurable clinical outcomes |
| Singer, F et al. | 2018 | SwissMTB: establishing comprehensive molecular cancer diagnostics in Swiss clinics | BMC MEDICAL INFORMATICS AND DECISION MAKING | No | No measurable clinical outcomes |

| | | | | | |
|---|---|---|---|---|---|
| | 2018 | Molecular Tumor Board: Resistance to Checkpoint Inhibitors | ONCOLOGIST | No | |
| | 2018 | MOLECULAR PROFILING OF ADVANCED SOFT-TISSUE SARCOMAS | COCHRANE | | Same population already included |
| Krämer, A et al. | 2018 | Comprehensive profiling and molecularly guided therapy (MGT) for carcinomas of unknown primary (CUP): CUPISCO: a phase II, randomised, multicentre study comparing targeted therapy or immunotherapy with standard platinum-based chemotherapy | ESMO OPEN | No | Abstract or Poster |
| Khater, F et al. | 2019 | Molecular Profiling of Hard-to-Treat Childhood and Adolescent Cancers | JAMA NETWORK OPEN | No | No measurable clinical outcomes |
| | 2019 | CUPISCO study: molecularly guided therapy versus standard chemotherapy in patients with carcinoma of unknown primary | COCHRANE | No | |
| | 2019 | A feasibility study of biologically focused therapy for myelodysplastic syndrome patients refractory to hypomethylating agents | COCHRANE | No | No measurable clinical outcomes |
| NCT03784014 | 2019 | Molecular Profiling of Advanced Soft-tissue Sarcomas | ClinicalTrials.gov | No | Same population already included |
| FGM 2025 Workflow Study Group | 2020 | Feasibility of high-throughput sequencing in clinical routine cancer care: lessons from the cancer pilot project of the France Genomic Medicine 2025 plan | ESMO OPEN | No | No measurable clinical outcomes |
| Luchini, C et al. | 2020 | Molecular Tumor Board for Metastatic Prostate Cancer in Routine Clinical Practice | ONCOLOGY RESEARCH AND TREATMENT | No | No full text available |

| | | | | | |
|---|---|---|---|---|---|
| | 2020 | Personalized Medicine for Neuro-Oncology Patients: Implementation and Outcome of the Molecular Tumor Board Tuebingen | ONCOLOGY RESEARCH AND TREATMENT | No | No full text available |
| Grossman, JE et al. | 2020 | GI oncology molecular tumor board: Fostering collaboration and clinical education for personalized therapy | JOURNAL OF CLINICAL ONCOLOGY | No | Abstract or Poster |
| | 2020 | The CCC Munichlmu Molecular Tumor Board: Clinical and Molecular Characteristics of the First 450 Patients | ONCOLOGY RESEARCH AND TREATMENT | No | No full text available |
| Rybkin, II et al. | 2020 | Implementation and impact of the first two years of a systemwide molecular tumor board at Henry Ford Health System (HFHS) | JOURNAL OF CLINICAL ONCOLOGY | No | Abstract or Poster |
| | 2020 | Molecular tumor boards: ethical challenges and practical recommendations | ONCOLOGY RESEARCH AND TREATMENT | No | No full text available |
| Tamborero, D et al. | 2020 | Support systems to guide clinical decision-making in precision oncology: The Cancer Core Europe Molecular Tumor Board Portal | Nature Medicine | No | No measurable clinical outcomes |
| von Baumgarten, L et al. | 2020 | Therapeutic management of neuro-oncologic patients - potential relevance of CSF liquid biopsy | THERANOSTICS | No | No measurable clinical outcomes |
| Walter, C et al. | 2020 | Sequencing for an interdisciplinary molecular tumor board in patients with advanced breast cancer-experiences from a case series | Oncotarget | No | Wrong study design |
| Watson, C et al. | 2020 | The prevalence of germline mutations among patients with solid tumors with genomic alterations identified on tumor testing: Results from a tertiary care academic center molecular tumor board | JOURNAL OF CLINICAL ONCOLOGY | No | Abstract or Poster |

| | | | | | |
|---|---|---|---|---|---|
| | 2020 | The Rome Trial From Histology to Target: the Road to Personalize Target Therapy and Immunotherapy | COCHRANE | No | Same population already included |
| NCT04641676 | 2020 | A Study to Examine the Value of Broad Agnostic Next Generation Sequencing (NGS) Panel Testing Versus Reimbursed Organ-directed NGS: a Belgian Precision Study of the BSMO in Collaboration With the Cancer Center | ClinicalTrials.gov | No | Abstract or Poster |
| NCT04258137 | 2020 | Circulating DNA to Improve Outcome of Oncology PatiEnt. A Randomized Study | ClinicalTrials.gov | No | No MTB |
| NCT06030869 | 2020 | MyCustom:Prospective Master Protocol Trial on Precision Medicine Treatment for Refractory Solid Tumors | ClinicalTrials.gov | No | No full text available |
| Brandão, M et al. | 2021 | Survival Impact and Cost-Effectiveness of a Multidisciplinary Tumor Board for Breast Cancer in Mozambique, Sub-Saharan Africa | ONCOLOGIST | No | No MTB |
| ElNaggar, A et al. | 2021 | Impact of Molecular Tumor Board (MTB) on precision oncology in a community setting | GYNECOLOGIC ONCOLOGY | No | Abstract or Poster |
| Forschner, A et al. | 2021 | Case Report: Combined CDK4/6 and MEK Inhibition in Refractory CDKN2A and NRAS Mutant Melanoma | FRONTIERS IN ONCOLOGY | No | No measurable clinical outcomes |
| Fumagalli, C et al. | 2021 | Making the Most of Complexity to Create Opportunities: Comprehensive Genomic Profiling and Molecular Tumor Board for Patients with Non-Small Cell Lung Cancer (NSCLC) | Cancers (Basel) | No | No measurable clinical outcomes |

| | | | | | |
|---|---|---|---|---|---|
| Green, MF et al. | 2021 | Implementation of a Molecular Tumor Registry to Support the Adoption of Precision Oncology Within an Academic Medical Center: The Duke University Experience | JCO PRECISION ONCOLOGY | No | No measurable clinical outcomes |
| Inagaki, C et al. | 2021 | Clinical Utility of Next-Generation Sequencing-Based Panel Testing under the Universal Health-Care System in Japan: A Retrospective Analysis at a Single University Hospital | CANCERS | No | No measurable clinical outcomes |
| Kondo, T et al. | 2021 | Comprehensive genomic profiling for patients with chemotherapy-naive advanced cancer | CANCER SCIENCE | No | No measurable clinical outcomes |
| | 2021 | Molecular Tumor Board for the Control of Therapy Decisions and Quality Assurance in Patients with Lung Cancer | PNEUMOLOGIE | No | Foreign language |
| Ludwig, SV et al. | 2021 | Impact of comprehensive genomic profiling and molecular tumor board decision on clinical outcome of patients with solid tumors: A single center, retrospective analysis | ANNALS OF ONCOLOGY | No | Abstract or Poster |
| Rybkin, II et al. | 2021 | Molecular tumor board impact at two large health systems | MOLECULAR CANCER THERAPEUTICS | No | Abstract or Poster |
| Sadaps, M et al. | 2021 | The impact of clinical decision making in a molecular tumor board at a tertiary care center | JOURNAL OF CLINICAL ONCOLOGY | No | Abstract or Poster |
| Seet, AOL et al. | 2021 | Individualized Molecular Profiling for Allocation to Clinical Trials Singapore Study-An Asian Tertiary Cancer Center Experience | JCO Precis Oncol | No | No measurable clinical outcomes |

| | | | | | |
|---|---|---|---|---|---|
| Walsh, EM et al. | 2021 | Molecular Tumor Board Guides Successful Treatment of a Rare, Locally Aggressive, Uterine Mesenchymal Neoplasm | JCO PRECISION ONCOLOGY | No | Wrong study design |
| Bonneville-Levard, A et al. | 2021 | Molecular profile to guide personalized medicine in adult patients with primary brain tumors: results from the ProfiLER trial | MED ONCOL. | No | Same population already included |
| NCT05177666 | 2021 | a Prospective Registration Study for Patients With Advanced Refractory Solid Tumors | ClinicalTrials.gov | No | No full text available |
| NCT04859543 | 2021 | Pediatric Prospective Personalized Immune and Target Identification Trial | ClinicalTrials.gov | No | No full text available |
| NCT05741944 | 2021 | The Value of a Risk Prediction Tool (PERSARC) for Effective Treatment Decisions of Soft-tissue Sarcomas Patients | ClinicalTrials.gov | No | No measurable clinical outcomes |
| Amatruda, T et al. | 2022 | Clinical utility of a molecular tumor board (MTB) in a community oncology practice | JOURNAL OF CLINICAL ONCOLOGY | No | No full text available |
| Aoyagi, Y et al. | 2022 | Clinical utility of comprehensive genomic profiling in Japan: Result of PROFILE-F study | PLOS ONE | No | No measurable clinical outcomes |
| Behel, V et al. | 2022 | Impact of Molecular Tumor Board on the Clinical Management of Patients With Cancer | JCO GLOBAL ONCOLOGY | No | No measurable clinical outcomes |
| Cannon, TL et al. | 2022 | Concurrent BRAFV600E and BRCA Mutations in MSS Metastatic Colorectal Cancer: Prevalence and Case Series of mCRC patients with prolonged OS | Cancer Treatment and Research Communications | No | No measurable clinical outcomes |
| Farhangfar, CJ et al. | 2022 | Impact of a Clinical Genomics Program on Trial Accrual for Targeted Treatments: Practical | JCO CLINICAL CANCER INFORMATICS | No | No measurable clinical outcomes |

| | | | | | |
|---|---|---|---|---|---|
| | | Approach Overcoming Barriers to Accrual for Underserved Patients | | | |
| Fukada, I et al. | 2022 | Assessment of a cancer genomic profile test for patients with metastatic breast cancer | SCIENTIFIC REPORTS | No | No measurable clinical outcomes |
| Pruis, MA et al. | 2022 | Personalised selection of experimental treatment in patients with advanced solid cancer is feasible using whole-genome sequencing | British Journal of Cancer | No | No measurable clinical outcomes |
| Riva, RT et al. | 2022 | Implementing next generation sequencing (NGS)/molecular tumor board (MTB)-based precision oncology practice: One-year experience at the Verona University Hospital | ANNALS OF ONCOLOGY | No | Abstract or Poster |
| Rubens, JA et al. | 2022 | Infantile suprasellar tumor diagnosed as a pineoblastoma RB1 subgroup and treatment challenges: A pediatric SNO Molecular Tumor Board | NEURO-ONCOLOGY ADVANCES | No | No measurable clinical outcomes |
| Shreenivas, AV et al. | 2022 | Carcinoma of unknown primary: Molecular tumor board-based therapy | CA Cancer Journal for Clinicians | No | No measurable clinical outcomes |
| Sunami, K et al. | 2022 | Impact of learning program on treatment recommendations by molecular tumor boards and an artificial intelligence-based annotation system: A prospective study | JOURNAL OF CLINICAL ONCOLOGY | No | Abstract or Poster |
| Sunami, K et al. | 2022 | Chronological improvement in precision oncology implementation in Japan | CANCER SCIENCE | No | No measurable clinical outcomes |
| Taskén, K et al. | 2022 | A national precision cancer medicine implementation initiative for Norway | Nat Med | No | No measurable clinical outcomes |

| | | | | | |
|---|---|---|---|---|---|
| | 2022 | Pragmatic Randomized Clinical Trial Comparing Molecular Tumor Board Assisted Care to Usual Care, | COCHRANE | No | No measurable clinical outcomes |
| | 2022 | THE ROME TRIAL: UPDATE ANALYSIS OF THE ACTIVITY OF MOLECULAR TUMOR BOARD | COCHRANE | No | Same population already included |
| | 2022 | Molecular landscape and actionable alterations in a genomic-guided cancer clinical trial: first analysis of the ROME trial | COCHRANE | No | Same population already included |
| | 2022 | Increasing targeted therapy options for patients with relapsed cancer with broader somatic gene panel analysis from the primary tumor: the Profiler02 randomized phase II trial | COCHRANE | No | Abstract or Poster |
| | 2022 | Personalized Medicine for Advanced Biliary Cancer Patients | COCHRANE | No | No measurable clinical outcomes |
| | 2022 | BRE12-158: a Postneoadjuvant, Randomized Phase II Trial of Personalized Therapy Versus Treatment of Physicianâ€™s Choice for Patients With Residual Triple-Negative Breast Cancer | COCHRANE | No | Same population already included |
| | 2022 | 1665P Genomic mutational landscape of solid tumors: preliminary results from ROME trial | COCHRANE | No | Same population already included |
| NCT06076070 | 2022 | GENomic PROfilation for Therapeutic Purposes in SARComas and Molecular Tumor Board (MTB): Retrospective/Prospective Study in Referral Centers | ClinicalTrials.gov | No | No full text available |
| Morfouace, M et al. | 2023 | Comprehensive molecular profiling of sarcomas in adolescent and young adult patients: Results of the | European Journal of Cancer | No | No measurable clinical outcomes |

| | | | | | |
|---|---|---|---|---|---|
| | | EORTC SPECTA-AYA international proof-of-concept study | | | |
| Elsey, R et al | 2023 | Evaluating the impact of a molecular tumor board in a community oncology center: Results from the Avera Cancer Institute's experience | Journal of Clinical Oncology | No | Abstract or Poster |
| Hee Peh, K et al | 2023 | Clinical utility of a regional precision medicine molecular tumor board and challenges to implementation | J Oncol Pharma Pract | No | No measurable clinical outcomes |
| Brahmi, M et al | 2023 | Large versus limited molecular profiling panel screening program in patients with metastatic sarcoma: an exploratory subgroup analysis from the ProfiLER 02 trial | Journal of Clinical Oncology | No | Same population already included |
| Ueda, T et al | 2023 | Comprehensive Genomic Profiling Detects Hereditary Cancers and Confers Survival Advantage in Patients With Gynaecological Cancers | Anticancer Res | No | No full text available |
| Alshammari, K et al. | 2023 | Outcomes of molecular tumor board recommendations for cancer patients with progression on standard of care therapies in Saudi Arabia | CANCER RESEARCH | No | Abstract or Poster |
| Ballatore, Z et al. | 2023 | Molecular Tumour Board (MTB): From Standard Therapy to Precision Medicine | JOURNAL OF CLINICAL MEDICINE | No | No measurable clinical outcomes |
| Benery, M et al. | 2023 | Leveraging Large Language Models for Decision Support in Personalized Oncology | JAMA Network Open | No | No measurable clinical outcomes |
| Botsis, T et al. | 2023 | Precision Oncology Core Data Model to Support Clinical Genomics Decision Making | JCO Clin Cancer Inform | No | No measurable clinical outcomes |

| | | | | | |
|---|---|---|---|---|---|
| Crimini, E et al. | 2023 | Characteristics and Survival Outcomes of Breast Cancer Patients Discussed at Molecular Tumor Board of European Institute of Oncology | BREAST | No | Abstract or Poster |
| Gallagher, D et al. | 2023 | International molecular tumour board: A transatlantic precision oncology collaboration | JOURNAL OF CLINICAL ONCOLOGY | No | No full text available |
| Heinrich, K et al. | 2023 | Lessons learned: the first consecutive 1000 patients of the CCCMunich(LMU) Molecular Tumor Board | J Cancer Res Clin Oncol | No | No measurable clinical outcomes |
| Limousin, W et al. | 2023 | Molecular-based targeted therapies in patients with hepatocellular carcinoma and hepato-cholangiocarcinoma refractory to atezolizumab/bevacizumab | Journal of Hepatology | No | No measurable clinical outcomes |
| Rossi, A et al. | 2023 | From seed to harvest: 18-month experience of molecular tumor board (MTB)-based precision oncology practice at the Verona University Hospital | JOURNAL OF CLINICAL ONCOLOGY | No | Abstract or Poster |
| Shirota, H et al. | 2023 | Clinical decisions by the molecular tumor board on comprehensive genomic profiling tests in Japan: A retrospective observational study | CANCER MEDICINE | No | No measurable clinical outcomes |
| Vingiani, A et al. | 2023 | Molecular Tumor Board as a Clinical Tool for Converting Molecular Data Into Real-World Patient Care | JCO Precis Oncol | No | No measurable clinical outcomes |
| Vodicska, B et al. | 2023 | Real-world performance analysis of a novel computational method in the precision oncology of pediatric tumors | WORLD JOURNAL OF PEDIATRICS | No | No measurable clinical outcomes |
| Wadensten, E et al. | 2023 | Diagnostic Yield From a Nationwide Implementation of Precision Medicine for all Children With Cancer | JCO Precis Oncol | No | No measurable clinical outcomes |

| | | | | | |
|---|---|---|---|---|---|
| | 2023 | Mutational landscape of breast cancer patients in ROME trial: preliminary results | COCHRANE | No | Same population already included |
| | 2023 | Prospective ctDNA genotyping for treatment selection in metastatic castration-resistant prostate cancer (mCRPC): the Canadian Cancer Trials Group phase II PC-BETS umbrella study | COCHRANE | No | Abstract or Poster |
| | 2023 | LBA16 Primary analysis of efficacy and safety in the CUPISCO trial: a randomised, global study of targeted therapy or cancer immunotherapy guided by comprehensive genomic profiling (CGP) vs platinum-based chemotherapy (CTX) in newly diagnosed, unfavourable cancer of unknown primary (CUP) | COCHRANE | No | Abstract or Poster |
| | 2023 | 70MO Genomic profiling to expand precision cancer medicine in the real world: the ROME trial | COCHRANE | No | Same population already included |
| NCT06022276 | 2023 | Nimotuzumab for EGFR-amplified Advanced Pan Solid Tumors | ClinicalTrials.gov | No | No full text available |
| Michaelis, J et al. | 2024 | Primary Results of Patients with Genitourinary Malignancies Presented at a Molecular Tumor Board | Urol Int. | No | No full text available |
| Moik, F et al. | 2024 | Practice patterns and treatment outcomes of molecular tumour board (MTB)-based personalized cancer therapies: A single-center experience | Biomarkers & translational research | No | Abstract or Poster |
| Knox, J.J. et al | 2024 | Early results of the PASS-01 trial: Pancreatic adenocarcinoma signature stratification for treatment-01. | Journal of Clinical Oncology | No | Abstract or Poster |

| | | | | | |
|---|---|---|---|---|---|
| Verret, B et al | 2024 | 1719O Multisarc: a randomized precision medicine study in advanced soft-tissue sarcomas | Sarcoma | No | Abstract or Poster |
| Ursprung, S et al. | 2024 | Standardized Response Assessment in Patients with Advanced Cholangiocarcinoma Treated with Personalized Therapy. | J Pers Med | No | No measurable clinical outcomes |
| Tsimberidou, AM et al | 2024 | Results of IMPACT 2, a randomized study evaluating molecular profiling and targeted agents in metastatic cancer at MD Anderson Cancer Center | Journal of Clinical Oncology | No | Abstract or Poster |
