# Supplementary material for: Molecular Tumor Boards clinical impact on patient care and structural features: A systematic review and meta-analysis
Source: PLoS Med. 2026 Jun 9;23(6):e1005125. doi: 10.1371/journal.pmed.1005125 (PMC13274928; doi:10.1371/journal.pmed.1005125)
Supplement: Attachment — Submitted filename: PLOS Response.docx [file pmed.1005125.s007.docx]

Dear Dr Lentini,

Many thanks for submitting your manuscript "Molecular Tumor Boards clinical impact on patient care and structural features: a systematic review and metanalysis" (PMEDICINE-D-25-04203R1) to PLOS Medicine. The paper has been reviewed by subject experts and a statistician; their comments are included below and can also be accessed here:

After discussing the paper with the editorial team and an academic editor with relevant expertise, I'm pleased to invite you to revise the paper in response to the reviewers' comments. As you will see, the reviewers find your study to be valuable for the field but they have raised some important concerns around methodology and requested some clarification, that we would ask you to address in full.
We plan to send the revised paper to some or all of the original reviewers and additionally to one extra subject expert for input, and we cannot provide any guarantees at this stage regarding publication.

In addition to these revisions, you may need to complete some formatting changes, which you will receive in a follow up email. A member of our team will be in touch with a set of requests shortly. If you do not receive a separate email within a few days, please assume that checks have been completed, and no additional changes are required.

When you upload your revision, please include a point-by-point response that addresses all of the reviewer and editorial points, indicating the changes made in the manuscript and either an excerpt of the revised text or the location (eg: page and line number) where each change can be found. Please also be sure to check the general editorial comments at the end of this letter and include these in your point-by-point response. When you resubmit your paper, please include a clean version of the paper as the main article file and a version with changes tracked as a marked-up manuscript. It may also be helpful to check the guidelines for revised papers at [https://eur03.safelinks.protection.outlook.com/?url=http%3A%2F%2Fjournals.plos.org%2Fplosmedicine%2Fs%2Frevising-your-manuscript&data=05%7C02%7Cnicolo.lentini%40unicatt.it%7C0efbecadbf6c4106051208de698c93ed%7Cb94f7d7481ff44a9b5886682acc85779%7C0%7C0%7C639064249680263304%7CUnknown%7CTWFpbGZsb3d8eyJFbXB0eU1hcGkiOnRydWUsIlYiOiIwLjAuMDAwMCIsIlAiOiJXaW4zMiIsIkFOIjoiTWFpbCIsIldUIjoyfQ%3D%3D%7C0%7C%7C%7C&sdata=%2F6oXATMZKmKH5I7hBs0B1sKZLsEh2MeaLQhhM4sl%2FmE%3D&reserved=0](https://eur03.safelinks.protection.outlook.com/?url=http%3A%2F%2Fjournals.plos.org%2Fplosmedicine%2Fs%2Frevising-your-manuscript&data=05%7C02%7Cluigi.russo12%40icatt.it%7C8686c38b441043738b4808de6990ae2b%7C080496d6a52847a18c2af8860f43342f%7C0%7C0%7C639064267287720843%7CUnknown%7CTWFpbGZsb3d8eyJFbXB0eU1hcGkiOnRydWUsIlYiOiIwLjAuMDAwMCIsIlAiOiJXaW4zMiIsIkFOIjoiTWFpbCIsIldUIjoyfQ%3D%3D%7C0%7C%7C%7C&sdata=V88t%2F5lqYR1%2BTLC5i%2B4iX7FY18Qr4cilQ76YPertJwY%3D&reserved=0) for any that apply to your paper.

We ask that you submit your revision by Mar 04 2026 11:59PM. However, if this deadline is not feasible, please contact me by email, and we can discuss a suitable alternative.

Don't hesitate to contact me directly with any questions (kstarowicz@plos.org).

Best regards,
Katarzyna

Katarzyna Starowicz, PhD
Associate Editor
PLOS Medicine
kstarowicz@plos.org

R: We thank the Editor for the opportunity to revise our manuscript.

We have addressed the reviewers’ comments point by point, as detailed below, and have included the additional analyses requested. All changes have been incorporated into the revised manuscript.

In addition, we have made the required formatting revisions, and we hope that the manuscript is now suitable for publication.

Reviewer #1: The authors have provided a comprehensive review and meta-analysis of the clinical efficacy of MTBs. Major positives include that it is the largest to date and the only one to stratify by study design. They also made intensive efforts to reach out to study PIs to obtain missing information.

Major concerns
1) The number of targeted therapies available have increased dramatically since 2015 when the SHIVA trial was reported (and 2012 when it was conceived). Could date of publication date be used to categorize trials as well?

R: We thank the reviewer for this important point. To account for the substantial evolution in the availability of targeted therapies over time, we conducted additional meta-regression analyses using year of publication as a covariate. This analysis was performed to explore whether the magnitude of the observed effects changed over time, particularly in relation to more recent trials conducted in a context of broader therapeutic options. The results of these analyses have been added to the manuscript (S12 Table and line 353-357).

2) The SHIVA trial had a predefined treatment algorithm that assigned a targeted therapy to a mutation in various pathways. The other studies included used an MTB team that made individual recommendations. The TAPUR and NCI-MATCH studies employed a similar approach to SHIVA (predefined drug/mutation assignment) and not included. Trials like SHIVA with prespecified treatments should be excluded.

R: We thank the Reviewer for this important comment.

We agree that trials relying exclusively on a prespecified drug–mutation assignment algorithm without MTB involvement should be excluded.

Regarding the specific trials mentioned:

TAPUR: Although TAPUR is largely based on predefined matching rules, the protocol specifies that the treating physician may request the support of an MTB in cases where no or multiple treatment matches are identified within the TAPUR matching rules, or when the physician wishes to propose a treatment match outside the predefined rules. Therefore, MTB involvement is not systematic but discretionary. For this reason, and given the lack of standardized MTB-based decision-making for all patients, TAPUR was excluded from our analysis.

NCI-MATCH: This study is primarily based on predefined drug–mutation assignments. To our knowledge, there is no published paper clearly describing whether and under what circumstances an MTB was systematically consulted. Due to this lack of clarity regarding MTB involvement, the trial was not included.

SHIVA: While the SHIVA trial used a predefined algorithm to assign single molecularly targeted agents, it systematically involved a molecular biology board in complex clinical scenarios. As reported by Le Tourneau et al., cases with multiple molecular alterations were reviewed by a molecular biology board to guide treatment selection. Therefore, unlike TAPUR and NCI-MATCH, SHIVA did incorporate structured expert board input in complex molecular situations, which justified its inclusion according to our predefined criteria.

The point raised by the Reviewer had also been previously assessed by our research group, and similar considerations were made. We fully understand the nature of this concern. However, based on our predefined criteria, we consider SHIVA an exception compared with trials that rely exclusively on fixed mutation-drug matching algorithms without systematic expert board involvement. Many such trials have been published in recent years and were not eligible for inclusion in our review.

A few suggestions

1) page 13, I don't think OS was consistently favored in the MTB guided across study designs. It was more of a trend in the randomized designs. Please revise

R: Thank you for the comment, we revised the manuscript accordingly (line 306),

2) The abstract conclusion is very well stated. I suggest revising the discussion to better support the conclusion by including a para on the need for high quality studies, and potentially point out that the lowest quality studies (observational) had the best outcomes as well as a para on the dilemmas facing health systems with regard to MTBs (is the effort worth the outcomes)

R: Thanks, we revised the text accordingly and added the two paragraphs (line 429-431 and 437-440).

Reviewer #2: Major comments

1) The review combines randomised controlled trials, non-randomised clinical trials, and observational (prospective and retrospective) studies within the same quantitative framework. Although stratification by study design is presented in figures and sensitivity analyses, the manuscript does not sufficiently justify the appropriateness of pooling these fundamentally different designs. Given the high risk of confounding and selection bias in non-randomised and observational studies, a clearer rationale is needed for the chosen synthesis strategy, including an explicit discussion of causal estimands and whether the pooled effects are intended to represent causal or associative effects.

R: We thank the reviewer for this important comment. We would like to clarify that we did not pool randomized, non-randomized, and observational studies within the same quantitative estimates. All meta-analyses were conducted separately and explicitly stratified by study design, and no combined pooled effect across different designs was calculated. Studies with different designs are presented together in figures solely for ease of visualization and comparison, not for quantitative synthesis. We have clarified this point in the Methods section (line 190-193) to avoid any misunderstanding. Accordingly, effect estimates should be interpreted as design-specific and with caution, particularly for non-randomized and observational studies, where confounding and selection bias remain important concerns, as we stated in discussion (line 415-427).

2) The definition and use of the Progression-Free Survival (PFS) ratio (≥1.3) as a binary outcome raises methodological concerns. This metric is highly sensitive to the choice of prior line of therapy and assumes comparability between pre- and post-MTB treatment periods, which is unlikely across heterogeneous clinical contexts. The manuscript should more clearly justify the statistical validity of this threshold-based outcome and discuss potential biases such as regression to the mean, immortal time bias, and informative censoring, particularly in observational settings.

R: We agree with the reviewer that the PFS ratio ≥1.3 is a methodologically limited metric and that it is sensitive to the choice of prior therapy and to the assumption of comparability between pre- and post-MTB treatment periods, which may not fully hold across heterogeneous clinical contexts. However, the PFS ratio ≥1.3 represents one of the most consistently reported outcome measures in the MTB literature and has been widely adopted as a patient-level indicator of clinical benefit in precision oncology since its original proposal by Von Hoff et al. (2010; 10.1200/JCO.2009.26.5983). Given its consistent use across studies, providing a pooled estimate helps contextualize the overall magnitude of benefit reported in the literature despite known limitations. We therefore reported only the proportion of patients achieving a PFS ratio ≥1.3 among those receiving MTB-guided therapy, without performing comparative analyses, in line with its original use in this clinical setting. The Methods (line 210-212) and Discussion (line 455-458) sections have been revised to further clarify the rationale for its inclusion and to explicitly acknowledge potential sources of bias, including regression to the mean, immortal time bias, and informative censoring.

3) Survival outcomes (OS and PFS) are synthesised despite substantial heterogeneity in the definition of time origin across studies (e.g., randomisation, treatment initiation, MTB discussion, diagnosis, or consent). Although sensitivity analyses restricted to studies measuring survival from treatment initiation are presented, this issue remains a major threat to internal validity. The primary analyses should more explicitly acknowledge that pooled hazard ratios may not be directly comparable across studies with different time zero definitions, and stronger emphasis should be placed on these sensitivity analyses in the main results and interpretation.

R: We agree with the reviewer that heterogeneity in the definition of the time origin for survival outcomes represents an important threat to internal validity. For this reason, we explicitly acknowledged it in the Methods (line 237-240) and in the main Results section (line 361-363), and we also placed specific emphasis on sensitivity analyses restricted to studies measuring survival from treatment initiation, which yielded consistent results.

In addition, we expanded the Discussion (line 423-425 and 462-465) to explicitly state that pooled hazard ratios may not be directly comparable across studies with different time zero definitions and should therefore be interpreted with appropriate caution.

4) The manuscript reports heterogeneity statistics (I²) but provides limited exploration of its sources beyond stratification by study design. Given the wide clinical and methodological heterogeneity across included studies (tumor types, prior lines of therapy, MTB composition, comparator definitions, sequencing technologies), additional exploratory analyses (e.g., meta-regression or subgroup analyses by key effect modifiers) would be methodologically appropriate, or at minimum, the limitations arising from unaddressed heterogeneity should

We thank the Reviewer for this important comment. Stratification by study design reduced between-study heterogeneity in several analyses, suggesting that study design may represent an important contributor to overall variability. However, residual heterogeneity persisted in some strata, indicating that additional clinical and methodological factors likely influenced the observed effects.

To further explore potential effect modifiers, we conducted meta-regression analyses including study design as a covariate, alongside other study-level characteristics. These analyses were limited by incomplete reporting of several clinically relevant variables (e.g., prior lines of therapy), which constrained a more comprehensive assessment of heterogeneity.

We have clarified the meta-regression strategy in the Methods section (line 229-237) and expanded the Results accordingly (S12 Table) and line 353-357). In addition, 95% prediction intervals were calculated for all random-effects models to better reflect the expected range of effects in comparable future settings.

Finally, we have expanded the Discussion section (line 442-448) to more explicitly acknowledge the remaining clinical and methodological heterogeneity that could not be fully addressed.

5) For non-randomised and observational studies with comparators, it is unclear whether the extracted effect estimates are adjusted or unadjusted, and how differences in covariate adjustment across studies were handled in the meta-analysis. Pooling adjusted and unadjusted estimates without clear distinction may bias the summary effects. The Methods should clarify which estimates were preferentially extracted and whether any hierarchy or standardization was applied.

We thank the Reviewer for this important comment. We have revised the Methods section (line 202-206) to clarify that adjusted effect estimates were preferentially extracted whenever available. When adjusted estimates were not reported, unadjusted estimates were extracted.

Information regarding whether each effect estimate was adjusted or unadjusted has now been systematically reported and included in S3 Table to ensure transparency.

6) Risk-of-bias assessments are presented using appropriate tools, but their integration into the quantitative synthesis is limited. While sensitivity analyses excluding studies at serious or critical risk of bias are provided, the main pooled estimates still include these studies. The manuscript would benefit from a clearer explanation of how risk of bias influenced interpretation of the primary findings, particularly for outcomes dominated by observational evidence.

R: We agree with the reviewer that risk of bias is a key element in the interpretation of our findings, particularly for outcomes largely informed by observational evidence. Although the primary analyses include all eligible studies, we conducted sensitivity analyses excluding studies rated as having a serious or critical risk of bias, which did not materially change the direction of the pooled estimates. To address this concern more explicitly, we have further emphasized in the Discussion (line 429-431) that, despite the robustness of these sensitivity analyses, effect estimates derived from non-randomized and observational studies should be interpreted with caution given their underlying risk-of-bias profiles.

7) The certainty of evidence assessment (e.g., GRADE or equivalent) is mentioned but remains difficult to interpret in light of the extensive heterogeneity, mixed study designs, and outcome definition issues. The rationale for rating decisions should be more transparent, especially for outcomes where statistically significant pooled effects are driven primarily by observational retrospective studies.

R: We thank the reviewer for this thoughtful comment. We considered applying a formal GRADE assessment; however, we judged that it would provide limited additional value in this context. The majority of the included evidence derives from non-randomized and observational studies, which are, by definition, initially rated as low certainty within the GRADE framework, with further downgrading likely due to risk of bias, inconsistency, and indirectness. Applying GRADE separately by study design would therefore largely reproduce the information already captured by the RoB2 and ROBINS-I assessments.

Instead, we chose to explicitly and extensively discuss the methodological limitations, sources of bias, and interpretative caution required when considering these findings, opting to focus on transparent reporting of risk of bias and careful interpretation of effect estimates rather than on formal certainty ratings. Moreover, as the objective of this review was to synthesize and critically appraise the available evidence on the clinical impact of MTBs, rather than to support clinical recommendations, the practical added value of a formal certainty-of-evidence grading is limited, and we believed this approach was more appropriate and informative for readers.

Minor comments
8) It should be clarified whether random-effects models were used uniformly across all syntheses and, if so, which estimator (e.g., DerSimonian-Laird, REML) was applied. Justification for the chosen approach would improve methodological transparency.

We thank the Reviewer for this important comment. We confirm that random-effects models were applied uniformly across all quantitative syntheses. Between-study variance was estimated using the restricted maximum-likelihood (REML) method, as specified in the Methods section. We selected REML because it provides more robust and less biased estimates of between-study variance compared with traditional approaches such as DerSimonian–Laird, particularly in the presence of expected moderate heterogeneity or a limited number of studies (Langan 2019, 10.1002/jrsm.1316). We have clarified this rationale in the revised Methods section (line 216.218) to improve methodological transparency.

9) Funnel plots are presented stratified by study design; however, their interpretability is limited in strata with a small number of studies. This limitation should be acknowledged more explicitly, and strong conclusions regarding publication bias should be avoided.

We thank the Reviewer for this important observation. We acknowledge that the interpretability of funnel plots is limited in strata with a small number of studies. We have revised the Results (line 358-360) and Discussion sections (line 459-462) to adopt a more cautious interpretation of the findings related to publication bias. Although the graphical and statistical assessments did not suggest substantial asymmetry, we have tempered our conclusions in light of the limited number of studies within each stratum.

10) For binary outcomes such as ORR and DCR, the handling of zero-event studies and continuity corrections should be described, as these choices can materially affect pooled relative risks.

We thank the Reviewer for this important comment. For binary outcomes (ORR and DCR), a continuity correction of 0.5 was specified to be applied only to studies with at least one zero cell. However, no included study had zero events in either arm; therefore, no continuity correction was ultimately required. Studies with zero events in both arms would have been excluded, as they do not contribute information to the estimation of relative risks. We have clarified this in the Methods section (line 207-212).

11) The Methods section would benefit from a concise summary table linking each outcome to its effect measure, eligible study designs, and main sensitivity analyses, to improve clarity for readers.

R: We thank the Reviewer for this helpful suggestion. To improve clarity and facilitate interpretation, we have added a concise summary table in the Supplementary Materials (S2 Table) linking each outcome to its corresponding effect measure, eligible study designs, and main sensitivity analyses.

12) The manuscript should ensure consistent terminology when referring to "comparators," particularly distinguishing between standard-of-care, physician's choice, non-targeted therapy, and absence of actionable alterations, as these are not methodologically equivalent comparison groups.

We thank the Reviewer for this important comment. We acknowledge that the comparator groups across studies were not methodologically equivalent (e.g., standard-of-care, physician’s choice, non-targeted therapy, or absence of actionable alterations). In our analyses, we used the term “comparator” to collectively refer to the non-MTB groups, as the nature of the comparison varied across studies and could not be consistently categorized into mutually exclusive subgroups.

To address this potential source of heterogeneity and ensure methodological rigor, we have conducted an additional meta-regression analysis including the type of comparator as a study-level covariate. The results of this analysis are now reported in the results section (line 353-357) and in S12 Table.

Reviewer #3: Summary and general assessment
The authors aim to evaluate whether receiving Molecular Tumor Board (MTB)-recommended treatments is associated with improved clinical outcomes, assessed using multiple effect measures, including hazard ratios (HRs) for overall survival (OS) and progression-free survival (PFS), relative risks (RRs) for objective response rate (ORR) and disease control rate (DCR), and pooled proportions for a PFS ratio ≥ 1.3. A total of 78 studies were identified through a comprehensive literature review, encompassing heterogeneous study designs, including randomized controlled trials (RCTs), non-randomized clinical trials, prospective studies, and retrospective studies.
The authors extracted general study information, MTB characteristics, patient and tumor characteristics, and clinical outcomes, including the starting point for survival time measurement. In addition, they contacted principal investigators of RCTs to obtain clarifications and additional data where needed.
For each study design category, pooled effect sizes were estimated using random-effects meta-analysis and reported with 95% confidence intervals. Prediction intervals were not reported. Across most outcomes and study designs, MTB-guided treatment was associated with improved clinical outcomes compared with non-MTB approaches.
Given the substantial heterogeneity across studies — including differences in cancer types and study design — the authors appropriately conducted sensitivity analyses, including leave-one-out analyses and exclusion of studies at high risk of bias. These analyses demonstrated that the direction of the pooled estimates remained stable, supporting the robustness of the overall findings. The authors also assessed potential publication bias and performed tests for excess significance, finding no clear evidence of publication bias.

Heterogeneity and meta-regression
To explore potential sources of between-study heterogeneity, the authors performed meta-regression analyses using study-level covariates. Notably, for OS, studies focusing on a single cancer type were associated with an odds ratio of 0.88 and accounted for 78.4% of the between-study variance, while for ORR this covariate explained 59.3% of the variance.
These findings suggest that whether a study focuses on a single versus multiple cancer types may be a major contributor to heterogeneity in reported outcomes. However, the manuscript does not clearly specify whether the meta-regression analyses were conducted jointly across all study designs with study design included as a covariate, or separately within each study-design category. Given the substantial differences in effect sizes and risk of bias across study designs, clarifying this point would be important for the interpretation of the meta-regression results.

Risk of bias and limitations
The authors assessed risk of bias using RoB 2 for randomized controlled trials and ROBINS-I for non-randomized studies. As expected, the majority of non-randomized studies were judged to have moderate to high risk of bias, whereas RCTs generally showed lower risk of bias.
The authors appropriately acknowledge that the overall evidence base is dominated by non-randomized studies with substantial risk of bias, which limits causal interpretation of the pooled estimates. This limitation is clearly stated and appropriately discussed.

Additional comments
Major comments
1. Prediction intervals
Given the substantial heterogeneity across studies, reporting prediction intervals in addition to confidence intervals would provide clinically relevant information on the expected range of effects in future studies.

We thank the Reviewer for this valuable suggestion. We have now calculated and reported 95% prediction intervals in addition to confidence intervals for all random-effects meta-analyses. This information has been added to the Methods (line 218-220) and Results sections (Table 3), and prediction intervals are now presented in all corresponding figures and tables.

2. Meta-regression strategy clarity
The manuscript would benefit from clearer reporting of the meta-regression strategy, including whether analyses were performed separately by study design or jointly across all study types, and whether covariates were included in univariable or multivariable models. In addition, it would be helpful to report the associations of study design, year of publication, and other study-level covariates with overall survival, as mentioned in the "Data synthesis and statistical methods" section.

We thank the Reviewer for this helpful comment. We have clarified the meta-regression strategy in the Methods section (line 229-237), specifying that the analyses were conducted across all included studies, with study design incorporated as covariate and additional study-level variables entered individually in separate models. To further enhance transparency, we have added S12 Table presenting the detailed results of the meta-regression analyses.

3. Study inclusion and exclusion
On page 13, the authors state that "of the 78 studies identified, 52 were included in the meta-analysis." Could the authors clarify the reasons for exclusion of the remaining 26 studies?

R: We thank the reviewer for this question. The 26 studies not included in the quantitative meta-analyses did not report effect measures or outcome data suitable for pooling, such as hazard ratios for overall survival or progression-free survival, or sufficient information to derive these estimates (e.g., median OS/PFS without time-to-event data). These studies are nonetheless summarized descriptively, and their reported outcomes are detailed in S6 Table.

4. Median OS reporting inconsistencies
On page 13 (OS section), median overall survival is reported for randomized controlled trials; however, this information is provided for only four studies, with one study missing. Additionally, in Supplementary Table 4, the median OS for the study by Schneider (2021) is missing. Clarification or completion of these data would improve transparency.

R: We thank the reviewer for this comment. One of the randomized controlled trials did not report median overall survival values and provided only hazard ratio estimates; therefore, median OS could not be extracted for this study and it is not reported in S6 Table, as these data were not available in the original publication. We added references next to the RCTs for the median OS and PFS (line 315 and 325).

Minor comments
1. Page 5: In the sentence "A multidisciplinary genomic pathway is currently being developed," the term "pathway" may be confusing for readers with a biological background, as it commonly refers to molecular signaling pathways. Consider clarifying or rephrasing this term.

R: We thank the reviewer for this suggestion. We have modified the sentence utilizing the word “framework” instead of “pathway” (line 111).

2. Page 5: In the sentence "We aim to evaluate the quality of evidence in order to district potential source of bias to make informed considerations," please consider replacing "district" with "identify."

R: We have modified the sentence accordingly (line 120).

3. Page 9: The sentence "For the analysis of patients with a PFS ratio ≥ 1.3, we calculated pooled proportions using …" could be revised for clarity, for example by explicitly stating that the pooled proportion was estimated to quantify the proportion of patients achieving a PFS ratio ≥ 1.3.

R: We thank the reviewer for this suggestion and have revised the sentence to clarify the concept (line 210-212).

4. Page 17: Please consider revising the sentence "The results clearly show a trend versus significant effects on …" to clarify the contrast between randomized and observational evidence and to avoid the phrasing "trend versus significant effects."

R: We thank the reviewer for this suggestion and have revised the sentence to clarify the concept (line 398-401).

Once again, we sincerely thank the Editor and the reviewers for their valuable comments and constructive suggestions, which helped to improve our manuscript.

Sincerely,

Dr. Nicolò Lentini
On behalf of the authors
