# Supplementary material for: Molecular Tumor Boards clinical impact on patient care and structural features: A systematic review and meta-analysis
Source: PLoS Med. 2026 Jun 9;23(6):e1005125. doi: 10.1371/journal.pmed.1005125 (PMC13274928; doi:10.1371/journal.pmed.1005125)
Supplement: Attachment — Submitted filename: Response_2_MTB_PLOS.docx [file pmed.1005125.s008.docx]

Dear Dr. Lentini,

Thank you very much for re-submitting your manuscript "Molecular Tumor Boards clinical impact on patient care and structural features: a systematic review and metanalysis" (PMEDICINE-D-25-04203R2) for review by PLOS Medicine.

I have discussed the paper with my colleagues and the academic editor and it was also seen again by three reviewers. I am pleased to say that provided the remaining editorial and production issues are dealt with we are planning to accept the paper for publication in the journal.

I did notice that you didn't provided a rebuttal for editorial comments and requests from previous decision letter. Please, include your responses to the points from previous decision letter followed by requests from this letter. Additionally, you have to provide point-to-point to reviewers comments. Without this we won't be able to proceed with the manuscript.

R: Dear Editor,

Thank you for your message, and we sincerely apologize for the confusion.

We reviewed the submission system and had already uploaded our previous responses; however, it is possible that some replies were inadvertently omitted or not properly visible. In any case, we have attached the full point-by-point response in this revision.

The remaining issues that need to be addressed are listed at the end of this email. Any accompanying reviewer attachments can be seen via the link below. Please take these into account before resubmitting your manuscript:

***Please note while forming your response, if your article is accepted, you may have the opportunity to make the peer review history publicly available. The record will include editor decision letters (with reviews) and your responses to reviewer comments. If eligible, we will contact you to opt in or out.***

In revising the manuscript for further consideration here, please ensure you address the specific points made by each reviewer and the editors. In your rebuttal letter you should indicate your response to the reviewers' and editors' comments and the changes you have made in the manuscript. Please submit a clean version of the paper as the main article file. A version with changes marked must also be uploaded as a marked up manuscript file.

Please also check the guidelines for revised papers at [https://eur03.safelinks.protection.outlook.com/?url=http%3A%2F%2Fjournals.plos.org%2Fplosmedicine%2Fs%2Frevising-your-manuscript&data=05%7C02%7Cnicolo.lentini%40unicatt.it%7C28d246d00e504765f20708de961d9e9b%7Cb94f7d7481ff44a9b5886682acc85779%7C0%7C0%7C639113251132274687%7CUnknown%7CTWFpbGZsb3d8eyJFbXB0eU1hcGkiOnRydWUsIlYiOiIwLjAuMDAwMCIsIlAiOiJXaW4zMiIsIkFOIjoiTWFpbCIsIldUIjoyfQ%3D%3D%7C0%7C%7C%7C&sdata=g0KXZVB33HNToFGS2IgcddmaQzMZfyWbrQU85uBpVtE%3D&reserved=0](https://eur03.safelinks.protection.outlook.com/?url=http%3A%2F%2Fjournals.plos.org%2Fplosmedicine%2Fs%2Frevising-your-manuscript&data=05%7C02%7Cluigi.russo12%40icatt.it%7C100bb5caf3c2422dc3f408de961f94f5%7C080496d6a52847a18c2af8860f43342f%7C0%7C0%7C639113259575321880%7CUnknown%7CTWFpbGZsb3d8eyJFbXB0eU1hcGkiOnRydWUsIlYiOiIwLjAuMDAwMCIsIlAiOiJXaW4zMiIsIkFOIjoiTWFpbCIsIldUIjoyfQ%3D%3D%7C0%7C%7C%7C&sdata=sUCFISTggT2glnRKKWSNqtxLfBF41wbc%2F2dN2yENvHQ%3D&reserved=0) for any that apply to your paper. If you haven't already, we ask that you provide a short, non-technical Author Summary of your research to make findings accessible to a wide audience that includes both scientists and non-scientists. The Author Summary should immediately follow the Abstract in your revised manuscript. This text is subject to editorial change and should be distinct from the scientific abstract.

R: We would like to confirm that this point has already been addressed in the previous revision.

In addition to these revisions, you may need to complete some formatting changes, which you will receive in a follow up email. A member of our team will be in touch with a set of requests shortly. If you do not receive a separate email within a few days, please assume that checks have been completed, and no additional changes are required.

We expect to receive your revised manuscript within 1 week. Please email us (kstarowicz@plos.org or plosmedicine@plos.org) if you have any questions or concerns.

We ask every co-author listed on the manuscript to fill in a contributing author statement. If any of the co-authors have not filled in the statement, we will remind them to do so when the paper is revised. If all statements are not completed in a timely fashion this could hold up the re-review process. Should there be a problem getting one of your co-authors to fill in a statement we will be in contact. YOU MUST NOT ADD OR REMOVE AUTHORS UNLESS YOU HAVE ALERTED THE EDITOR HANDLING THE MANUSCRIPT TO THE CHANGE AND THEY SPECIFICALLY HAVE AGREED TO IT.

Please ensure that the paper adheres to the PLOS Data Availability Policy (see [https://eur03.safelinks.protection.outlook.com/?url=http%3A%2F%2Fjournals.plos.org%2Fplosmedicine%2Fs%2Fdata-availability&data=05%7C02%7Cnicolo.lentini%40unicatt.it%7C28d246d00e504765f20708de961d9e9b%7Cb94f7d7481ff44a9b5886682acc85779%7C0%7C0%7C639113251132301623%7CUnknown%7CTWFpbGZsb3d8eyJFbXB0eU1hcGkiOnRydWUsIlYiOiIwLjAuMDAwMCIsIlAiOiJXaW4zMiIsIkFOIjoiTWFpbCIsIldUIjoyfQ%3D%3D%7C0%7C%7C%7C&sdata=ksX2ERLFY1Pqxr0mYPeRrK6rQGkwbAz2vGh9IzM2a0E%3D&reserved=0)](https://eur03.safelinks.protection.outlook.com/?url=http%3A%2F%2Fjournals.plos.org%2Fplosmedicine%2Fs%2Fdata-availability&data=05%7C02%7Cluigi.russo12%40icatt.it%7C100bb5caf3c2422dc3f408de961f94f5%7C080496d6a52847a18c2af8860f43342f%7C0%7C0%7C639113259575351111%7CUnknown%7CTWFpbGZsb3d8eyJFbXB0eU1hcGkiOnRydWUsIlYiOiIwLjAuMDAwMCIsIlAiOiJXaW4zMiIsIkFOIjoiTWFpbCIsIldUIjoyfQ%3D%3D%7C0%7C%7C%7C&sdata=RAYyiAT2AGldPiG1qxdvyGAtSkh5PxNe2PLLuVfKabY%3D&reserved=0), which requires that all data underlying the study's findings be provided in a repository or as Supporting Information. For data residing with a third party, authors are required to provide instructions with contact information for obtaining the data. PLOS journals do not allow statements supported by "data not shown" or "unpublished results." For such statements, authors must provide supporting data or cite public sources that include it.

To enhance the reproducibility of your results, we recommend that you deposit your laboratory protocols in protocols.io, where a protocol can be assigned its own identifier (DOI) such that it can be cited independently in the future. Additionally, PLOS ONE offers an option to publish peer-reviewed clinical study protocols. Read more information on sharing protocols at [https://eur03.safelinks.protection.outlook.com/?url=https%3A%2F%2Fplos.org%2Fprotocols%3Futm_medium%3Deditorial-email%26utm_source%3Dauthorletters%26utm_campaign%3Dprotocols&data=05%7C02%7Cnicolo.lentini%40unicatt.it%7C28d246d00e504765f20708de961d9e9b%7Cb94f7d7481ff44a9b5886682acc85779%7C0%7C0%7C639113251132319791%7CUnknown%7CTWFpbGZsb3d8eyJFbXB0eU1hcGkiOnRydWUsIlYiOiIwLjAuMDAwMCIsIlAiOiJXaW4zMiIsIkFOIjoiTWFpbCIsIldUIjoyfQ%3D%3D%7C0%7C%7C%7C&sdata=MRx9ICeiDaFfg1pWj0q4AOeiXSOKY%2B1QD5HIN7zAdjw%3D&reserved=0](https://eur03.safelinks.protection.outlook.com/?url=https%3A%2F%2Fplos.org%2Fprotocols%3Futm_medium%3Deditorial-email%26utm_source%3Dauthorletters%26utm_campaign%3Dprotocols&data=05%7C02%7Cluigi.russo12%40icatt.it%7C100bb5caf3c2422dc3f408de961f94f5%7C080496d6a52847a18c2af8860f43342f%7C0%7C0%7C639113259575376205%7CUnknown%7CTWFpbGZsb3d8eyJFbXB0eU1hcGkiOnRydWUsIlYiOiIwLjAuMDAwMCIsIlAiOiJXaW4zMiIsIkFOIjoiTWFpbCIsIldUIjoyfQ%3D%3D%7C0%7C%7C%7C&sdata=Ns28dxLm2IQ%2BNkD0niHPd8iSR40xdl7NPcd19qABmkE%3D&reserved=0)

Please review your reference list to ensure that it is complete and correct. If you have cited papers that have been retracted, please include the rationale for doing so in the manuscript text, or remove these references and replace them with relevant current references. Any changes to the reference list should be mentioned in the rebuttal letter that accompanies your revised manuscript.

Please note, when your manuscript is accepted, an uncorrected proof of your manuscript will be published online ahead of the final version, unless you've already opted out via the online submission form. If, for any reason, you do not want an earlier version of your manuscript published online or are unsure if you have already indicated as such, please let the journal staff know immediately at [plosmedicine@plos.org](mailto:plosmedicine@plos.org).

If you have any questions in the meantime, please contact me or the journal staff on [plosmedicine@plos.org.](mailto:plosmedicine@plos.org.)

We look forward to receiving the revised manuscript by Apr 16 2026 11:59PM.

Sincerely,

Katarzyna Starowicz, PhD

Associate Editor

PLOS Medicine

[kstarowicz@plos.org](mailto:kstarowicz@plos.org)

------------------------------------------------------------
General editorial requests:

(Note: not all will apply to your paper, but please check each item carefully)

* We ask every co-author listed on the manuscript to fill in a contributing author statement, making sure to declare all competing interests. If any of the co-authors have not filled in the statement, we will remind them to do so when the paper is revised. If all statements are not completed in a timely fashion this could hold up the re-review process. If new competing interests are declared later in the revision process, this may also hold up the submission. Should there be a problem getting one of your co-authors to fill in a statement we will be in contact. Please do not add or remove authors without first discussing this with the handling editor. You can see our competing interests policy here: <http://journals.plos.org/plosmedicine/s/competing-interests>.

R: We confirm we have no conflicts to declare.

* Please upload any figures associated with your paper as individual TIF or EPS files with 300dpi resolution at resubmission; please read our figure guidelines for more information on our requirements: <http://journals.plos.org/plosmedicine/s/figures>. While revising your submission, we strongly recommend that you use PLOS's NAAS tool (<https://ngplosjournals.pagemajik.ai/artanalysis>) to test your figure files. NAAS can convert your figure files to the TIFF file type and meet basic requirements (such as print size, resolution), or provide you with a report on issues that do not meet our requirements and that NAAS cannot fix.

After uploading your figures to PLOS's NAAS tool - <https://ngplosjournals.pagemajik.ai/artanalysis>, NAAS will process the files provided and display the results in the "Uploaded Files" section of the page as the processing is complete.

If the uploaded figures meet our requirements (or NAAS is able to fix the files to meet our requirements), the figure will be marked as "fixed" above. If NAAS is unable to fix the files, a red "failed" label will appear above.

When NAAS has confirmed that the figure files meet our requirements, please download the file via the download option, and include these NAAS processed figure files when submitting your revised manuscript.

R: We confirm that we follow the formatting rule of the figure.

* Please ensure that the paper adheres to the PLOS Data Availability Policy (see <http://journals.plos.org/plosmedicine/s/data-availability>), which requires that all data underlying the study's findings be provided in a repository or as Supporting Information. For data residing with a third party, authors are required to provide instructions with contact information (web or email address) for obtaining the data. Please note that a study author cannot be the contact person for the data. PLOS journals do not allow statements supported by "data not shown" or "unpublished results." For such statements, authors must provide supporting data or cite public sources that include it.

R: We confirm that the Data Availability formatting is correct.

* We expect all researchers with submissions to PLOS in which author-generated code underpins the findings in the manuscript to make all author-generated code available without restrictions upon publication of the work. In cases where code is central to the manuscript, we may require the code to be made available as a condition of publication. Authors are responsible for ensuring that the code is reusable and well documented. Please make any custom code available, either as part of your data deposition or as a supplementary file. Please add a sentence to your data availability statement regarding any code used in the study, e.g. "The code used in the analysis is available from Github [URL] and archived in Zenodo [DOI link]" Please review our guidelines at <https://journals.plos.org/plosmedicine/s/materials-software-and-code-sharing> and ensure that your code is shared in a way that follows best practice and facilitates reproducibility and reuse. Because Github depositions can be readily changed or deleted, we encourage you to make a permanent DOI'd copy (e.g. in Zenodo) and provide the URL.

R: We confirm that we published code and data on the Zenodo platform ([https://zenodo.org/records/19628910](https://doi.org/10.5281/zenodo.19628910)).

* Please ensure that the study is reported according to the PRISMA guideline and include the completed checklist as Supporting Information. When completing the checklist, please use section and paragraph numbers, rather than page numbers. Please add the following statement, or similar, to the Methods: "This study is reported as per [XXXX] guideline (S1 Checklist)."

R: The statement in the manuscript and the PRISMA checklist as Supporting Information have been included.

FORMATTING - GENERAL

* Abstract: Please structure your abstract using the PLOS Medicine headings (Background, Methods and Findings, Conclusions). Please combine the Methods and Findings sections into one section.

R: We confirm that the abstract is formatted in a compliant way.

* At this stage, we ask that you include a short, non-technical Author Summary of your research to make findings accessible to a wide audience that includes both scientists and non-scientists. The Author Summary should immediately follow the Abstract in your revised manuscript. This text is subject to editorial change and should be distinct from the scientific abstract. Ideally each sub-heading should contain 2-3 single sentence, concise bullet points containing the most salient points from your study. In the final bullet point of 'What Do These Findings Mean?', please include the main limitations of the study in non-technical language. Please see our author guidelines for more information: <https://journals.plos.org/plosmedicine/s/revising-your-manuscript#loc-author-summary>.

R: We confirm that we added non-technical Author Summary.

* Please express the main results with 95% CIs as well as p values. When reporting p values please report as p<0.001 and where higher as the exact p value p=0.002, for example. Throughout, suggest reporting statistical information as follows to improve clarity for the reader "22% (95% CI [13%,28%]; p</=)". Please be sure to define all numerical values at first use.

R: We have revised the manuscript, tables, and figures accordingly to standardize statistical reporting, including the format of confidence intervals, separation of lower and upper bounds with commas, and consistent repetition of statistical definitions (e.g., HR, CI) throughout the text.

* Please include page numbers and line numbers in the manuscript file. Use continuous line numbers (do not restart the numbering on each page).

R: We added page numbers and line numbers in the manuscript file.

* Please cite the reference numbers in square brackets. Citations should precede punctuation.

R: We corrected the reference style

FIGURES AND TABLES

* Please provide titles and legends for all figures and tables (including those in Supporting Information files).

R: We added all titles and legends for figures and tables

* Please define all abbreviations used in each figure/table (including those in Supporting Information files).

R: We added all abbreviations in each figure and tables.

* Please consider avoiding the use of red and green in order to make your figure more accessible to those with color blindness.

R: We have modified the figure and tables (for example S16 Table) where possible to avoid the use of red and green and improve accessibility.

SUPPLEMENTARY MATERIAL

* Please note that supplementary material will be posted as supplied by the authors. Therefore, please amend it according to the relevant comments outlined here.

R: We have modified all supplementary files according to the comments

* Please cite your Supporting Information as outlined here: <https://journals.plos.org/plosmedicine/s/supporting-information>

R: We have cited all supplementary files according

REFERENCES

* PLOS uses the numbered citation (citation-sequence) method and first six authors, et al.

R: We corrected the citation style accordingly.

* Please ensure that journal name abbreviations match those found in the National Center for Biotechnology Information (NCBI) databases (<http://www.ncbi.nlm.nih.gov/nlmcatalog/journals>), and are appropriately formatted and capitalised.

R: We corrected the citation style accordingly.

* Where website addresses are cited, please include the complete URL and specify the date of access (e.g. [accessed: 12/06/2023]).

R: We corrected the website-citation style accordingly.

* Please also see <https://journals.plos.org/plosmedicine/s/submission-guidelines#loc-references> for further details on reference formatting.

R: We corrected the citation style accordingly.

Requests from Editors:

* At this stage, we ask that you include a short, non-technical Author Summary of your research to make findings accessible to a wide audience that includes both scientists and non-scientists. The Author Summary should immediately follow the Abstract in your revised manuscript. This text is subject to editorial change and should be distinct from the scientific abstract. Ideally each sub-heading should contain 2-3 single sentence, concise bullet points containing the most salient points from your study. In the final bullet point of ‘What Do These Findings Mean?’ Please include the main limitations of the study in non-technical language.
Please see our author guidelines for more information: [https://eur03.safelinks.protection.outlook.com/?url=https%3A%2F%2Fjournals.plos.org%2Fplosmedicine%2Fs%2Frevising-your-manuscript%23loc-author-summary&data=05%7C02%7Cnicolo.lentini%40unicatt.it%7C28d246d00e504765f20708de961d9e9b%7Cb94f7d7481ff44a9b5886682acc85779%7C0%7C0%7C639113251132337886%7CUnknown%7CTWFpbGZsb3d8eyJFbXB0eU1hcGkiOnRydWUsIlYiOiIwLjAuMDAwMCIsIlAiOiJXaW4zMiIsIkFOIjoiTWFpbCIsIldUIjoyfQ%3D%3D%7C0%7C%7C%7C&sdata=C%2B1hVzk4pb9Hg0vWGBBncb4yphl9HX2oFk%2BPV%2B04wO0%3D&reserved=0."](https://eur03.safelinks.protection.outlook.com/?url=https%3A%2F%2Fjournals.plos.org%2Fplosmedicine%2Fs%2Frevising-your-manuscript%23loc-author-summary&data=05%7C02%7Cluigi.russo12%40icatt.it%7C100bb5caf3c2422dc3f408de961f94f5%7C080496d6a52847a18c2af8860f43342f%7C0%7C0%7C639113259575394883%7CUnknown%7CTWFpbGZsb3d8eyJFbXB0eU1hcGkiOnRydWUsIlYiOiIwLjAuMDAwMCIsIlAiOiJXaW4zMiIsIkFOIjoiTWFpbCIsIldUIjoyfQ%3D%3D%7C0%7C%7C%7C&sdata=FlHdirJYdUUmho1EDJuqexjL8RDbc3Xs3h9tB54kwfY%3D&reserved=0)

R: We would like to confirm that this point has already been addressed in the previous revision

* Please confirm that your title complies with PLOS Medicine's style. Your title must be nondeclarative and not a question. It should begin with main concept if possible. "Effect of" should be used only if causality can be inferred, i.e., for an RCT. Please place the study design ("A randomized controlled trial," "A retrospective study," "A modelling study," etc.) in the subtitle (ie, after a colon).

R: We confirm the title compliance.

* Please confirm that your abstract complies with our requirements, including format (three sections: Background, Methods and Findings, and Conclusions) and providing all the information relevant to this study type [https://eur03.safelinks.protection.outlook.com/?url=https%3A%2F%2Fjournals.plos.org%2Fplosmedicine%2Fs%2Fsubmission-guidelines%23loc-abstract&data=05%7C02%7Cnicolo.lentini%40unicatt.it%7C28d246d00e504765f20708de961d9e9b%7Cb94f7d7481ff44a9b5886682acc85779%7C0%7C0%7C639113251132355675%7CUnknown%7CTWFpbGZsb3d8eyJFbXB0eU1hcGkiOnRydWUsIlYiOiIwLjAuMDAwMCIsIlAiOiJXaW4zMiIsIkFOIjoiTWFpbCIsIldUIjoyfQ%3D%3D%7C0%7C%7C%7C&sdata=sXmfQLXuUWL8myJXphpl%2BYY9dypeIpGDNoHzsKKYXBw%3D&reserved=0](https://eur03.safelinks.protection.outlook.com/?url=https%3A%2F%2Fjournals.plos.org%2Fplosmedicine%2Fs%2Fsubmission-guidelines%23loc-abstract&data=05%7C02%7Cluigi.russo12%40icatt.it%7C100bb5caf3c2422dc3f408de961f94f5%7C080496d6a52847a18c2af8860f43342f%7C0%7C0%7C639113259575412702%7CUnknown%7CTWFpbGZsb3d8eyJFbXB0eU1hcGkiOnRydWUsIlYiOiIwLjAuMDAwMCIsIlAiOiJXaW4zMiIsIkFOIjoiTWFpbCIsIldUIjoyfQ%3D%3D%7C0%7C%7C%7C&sdata=uhsQE88FhmpFdp17WorGY6%2BrL723KbzDWmzb3Bi3aQc%3D&reserved=0) 
R: We confirm the abstract compliance.

* Please ensure that the Introduction ends with a clear description of the study question or hypothesis.

R: We confirm that the introduction complies with the requirements.

* Please ensure that all abbreviations are defined at first use throughout the text.
R: We confirm that all abbreviations are defined at first use, and we also added the full abbreviations list.

* Please confirm that all numbers presented in the abstract are present and identical to numbers presented in the main manuscript text.
R: We confirm.

* Please review your text for claims of novelty or primacy (e.g. 'for the first time') and remove this language. In addition, please check that any use of statistical terms (such as trend or significant) are supported by the data, and if not please remove them.

R: We confirm that the text is compliant with the requirements.

* Please remove the 'conclusions' subheading from the discussion. Please also remove any other subheadings from the discussion.
R: We revised the text accordingly.

* Statistical reporting: Please revise throughout the manuscript, including tables and figures.
- Please report statistical information as follows to improve clarity for the reader ""22% (95% CI [13,28]; p</=)"".
- Please separate upper and lower bounds with commas instead of hyphens as the latter can be confused with reporting of negative values.
- Please repeat statistical definitions (HR, CI etc.) for each set of parentheses."

R: We have revised the manuscript, tables, and figures accordingly to standardize statistical reporting, including the format of confidence intervals, separation of lower and upper bounds with commas, and consistent repetition of statistical definitions (e.g., HR, CI) throughout the text.

* In the last sentence of the Abstract Methods and Findings section, please describe the main limitation(s) of the study's methodology.
R: We have now added a brief statement in the last sentence of the Abstract Methods and Findings section describing the main methodological limitations of the study.

* In the abstract, please include the important dependent variables that are adjusted for in the analyses.

R: It was not applicable

* In the author summary, in the final bullet point of 'What Do These Findings Mean?', please include the main limitations of the study in non-technical language.

R: We included it.

* PLOS Medicine requires that the de-identified data underlying the specific results in a published article be made available, without restrictions on access, in a public repository or as Supporting Information at the time of article publication, provided it is legal and ethical to do so. Please see the policy at

[https://eur03.safelinks.protection.outlook.com/?url=http%3A%2F%2Fjournals.plos.org%2Fplosmedicine%2Fs%2Fdata-availability&data=05%7C02%7Cnicolo.lentini%40unicatt.it%7C28d246d00e504765f20708de961d9e9b%7Cb94f7d7481ff44a9b5886682acc85779%7C0%7C0%7C639113251132377543%7CUnknown%7CTWFpbGZsb3d8eyJFbXB0eU1hcGkiOnRydWUsIlYiOiIwLjAuMDAwMCIsIlAiOiJXaW4zMiIsIkFOIjoiTWFpbCIsIldUIjoyfQ%3D%3D%7C0%7C%7C%7C&sdata=GNmDFJRgcN0mFssM%2Bz%2Bd20yU1JW27GyB9VuVaW80ULY%3D&reserved=0](https://eur03.safelinks.protection.outlook.com/?url=http%3A%2F%2Fjournals.plos.org%2Fplosmedicine%2Fs%2Fdata-availability&data=05%7C02%7Cluigi.russo12%40icatt.it%7C100bb5caf3c2422dc3f408de961f94f5%7C080496d6a52847a18c2af8860f43342f%7C0%7C0%7C639113259575429375%7CUnknown%7CTWFpbGZsb3d8eyJFbXB0eU1hcGkiOnRydWUsIlYiOiIwLjAuMDAwMCIsIlAiOiJXaW4zMiIsIkFOIjoiTWFpbCIsIldUIjoyfQ%3D%3D%7C0%7C%7C%7C&sdata=HdtCWT5paGOY7Gm0Cifrx18RYnO0Q%2FWzQWF1Vu%2BSqv8%3D&reserved=0) 

and FAQs at

[https://eur03.safelinks.protection.outlook.com/?url=http%3A%2F%2Fjournals.plos.org%2Fplosmedicine%2Fs%2Fdata-availability%23loc-faqs-for-data-policy&data=05%7C02%7Cnicolo.lentini%40unicatt.it%7C28d246d00e504765f20708de961d9e9b%7Cb94f7d7481ff44a9b5886682acc85779%7C0%7C0%7C639113251132395204%7CUnknown%7CTWFpbGZsb3d8eyJFbXB0eU1hcGkiOnRydWUsIlYiOiIwLjAuMDAwMCIsIlAiOiJXaW4zMiIsIkFOIjoiTWFpbCIsIldUIjoyfQ%3D%3D%7C0%7C%7C%7C&sdata=69%2B2tlu1wZXMgGWiqhKvJ%2FddSnTAnNH15n2rE04jxhk%3D&reserved=0](https://eur03.safelinks.protection.outlook.com/?url=http%3A%2F%2Fjournals.plos.org%2Fplosmedicine%2Fs%2Fdata-availability%23loc-faqs-for-data-policy&data=05%7C02%7Cluigi.russo12%40icatt.it%7C100bb5caf3c2422dc3f408de961f94f5%7C080496d6a52847a18c2af8860f43342f%7C0%7C0%7C639113259575446702%7CUnknown%7CTWFpbGZsb3d8eyJFbXB0eU1hcGkiOnRydWUsIlYiOiIwLjAuMDAwMCIsIlAiOiJXaW4zMiIsIkFOIjoiTWFpbCIsIldUIjoyfQ%3D%3D%7C0%7C%7C%7C&sdata=Avumue40cK0dMFM9dFMbdT4C4%2FUy7QLr2QW6%2F6ZGpD4%3D&reserved=0)  "

R: We published code and data on the Zenodo platform ([https://zenodo.org/records/19628910](https://doi.org/10.5281/zenodo.19628910)).

* The funding statement should include: specific grant numbers, initials of authors who received each award, URLs to sponsors’ websites. Also, please state whether any sponsors or funders (other than the named authors) played any role in study design, data collection and analysis, the decision to publish, or preparation of the manuscript. If they had no role in the research, include this sentence: “The funders had no role in study design, data collection and analysis, decision to publish, or preparation of the manuscript.”

R: We confirm it is formatted in a compliant way.

* All authors must declare their relevant competing interests per the PLOS policy, which can be seen here: [https://eur03.safelinks.protection.outlook.com/?url=https%3A%2F%2Fjournals.plos.org%2Fplosmedicine%2Fs%2Fcompeting-interests&data=05%7C02%7Cnicolo.lentini%40unicatt.it%7C28d246d00e504765f20708de961d9e9b%7Cb94f7d7481ff44a9b5886682acc85779%7C0%7C0%7C639113251132412544%7CUnknown%7CTWFpbGZsb3d8eyJFbXB0eU1hcGkiOnRydWUsIlYiOiIwLjAuMDAwMCIsIlAiOiJXaW4zMiIsIkFOIjoiTWFpbCIsIldUIjoyfQ%3D%3D%7C0%7C%7C%7C&sdata=7JRW2LvVHR0GMf3l7j7V2Dt6tkGqXHOJEWnAwWOPIW4%3D&reserved=0](https://eur03.safelinks.protection.outlook.com/?url=https%3A%2F%2Fjournals.plos.org%2Fplosmedicine%2Fs%2Fcompeting-interests&data=05%7C02%7Cluigi.russo12%40icatt.it%7C100bb5caf3c2422dc3f408de961f94f5%7C080496d6a52847a18c2af8860f43342f%7C0%7C0%7C639113259575464018%7CUnknown%7CTWFpbGZsb3d8eyJFbXB0eU1hcGkiOnRydWUsIlYiOiIwLjAuMDAwMCIsIlAiOiJXaW4zMiIsIkFOIjoiTWFpbCIsIldUIjoyfQ%3D%3D%7C0%7C%7C%7C&sdata=5hBQjkj%2BHWJpiUtjF%2BYOx00GsbPRickYf7XhwzrYftU%3D&reserved=0) For authors with ties to industry, please indicate whether any of the interests has a financial stake in the results of the current study.

R: We confirm we have no conflicts to declare.

* PLOS Medicine requires that the de-identified data underlying the specific results in a published article be made available, without restrictions on access, in a public repository or as Supporting Information at the time of article publication, provided it is legal and ethical to do so. Please see the policy at

[https://eur03.safelinks.protection.outlook.com/?url=http%3A%2F%2Fjournals.plos.org%2Fplosmedicine%2Fs%2Fdata-availability&data=05%7C02%7Cnicolo.lentini%40unicatt.it%7C28d246d00e504765f20708de961d9e9b%7Cb94f7d7481ff44a9b5886682acc85779%7C0%7C0%7C639113251132431104%7CUnknown%7CTWFpbGZsb3d8eyJFbXB0eU1hcGkiOnRydWUsIlYiOiIwLjAuMDAwMCIsIlAiOiJXaW4zMiIsIkFOIjoiTWFpbCIsIldUIjoyfQ%3D%3D%7C0%7C%7C%7C&sdata=JwhZ%2Fd%2Boyje0PWIRqB%2F63WHzgG%2BaOeRkbmZw%2BBF%2Fft4%3D&reserved=0](https://eur03.safelinks.protection.outlook.com/?url=http%3A%2F%2Fjournals.plos.org%2Fplosmedicine%2Fs%2Fdata-availability&data=05%7C02%7Cluigi.russo12%40icatt.it%7C100bb5caf3c2422dc3f408de961f94f5%7C080496d6a52847a18c2af8860f43342f%7C0%7C0%7C639113259575480158%7CUnknown%7CTWFpbGZsb3d8eyJFbXB0eU1hcGkiOnRydWUsIlYiOiIwLjAuMDAwMCIsIlAiOiJXaW4zMiIsIkFOIjoiTWFpbCIsIldUIjoyfQ%3D%3D%7C0%7C%7C%7C&sdata=cBr%2FelLZox81Gt0Cn7l8K0eeloGuujg960V0JHQeupI%3D&reserved=0)  and FAQs at [https://eur03.safelinks.protection.outlook.com/?url=http%3A%2F%2Fjournals.plos.org%2Fplosmedicine%2Fs%2Fdata-availability%23loc-faqs-for-data-policy&data=05%7C02%7Cnicolo.lentini%40unicatt.it%7C28d246d00e504765f20708de961d9e9b%7Cb94f7d7481ff44a9b5886682acc85779%7C0%7C0%7C639113251132448791%7CUnknown%7CTWFpbGZsb3d8eyJFbXB0eU1hcGkiOnRydWUsIlYiOiIwLjAuMDAwMCIsIlAiOiJXaW4zMiIsIkFOIjoiTWFpbCIsIldUIjoyfQ%3D%3D%7C0%7C%7C%7C&sdata=GXbmDh2PBRifDq9Gtpaw%2BTTj46MOvo3U4WZWcDRAVFA%3D&reserved=0](https://eur03.safelinks.protection.outlook.com/?url=http%3A%2F%2Fjournals.plos.org%2Fplosmedicine%2Fs%2Fdata-availability%23loc-faqs-for-data-policy&data=05%7C02%7Cluigi.russo12%40icatt.it%7C100bb5caf3c2422dc3f408de961f94f5%7C080496d6a52847a18c2af8860f43342f%7C0%7C0%7C639113259575495744%7CUnknown%7CTWFpbGZsb3d8eyJFbXB0eU1hcGkiOnRydWUsIlYiOiIwLjAuMDAwMCIsIlAiOiJXaW4zMiIsIkFOIjoiTWFpbCIsIldUIjoyfQ%3D%3D%7C0%7C%7C%7C&sdata=iqFUyacXDiAYTkER%2FV3LbupBKRJVYZlIt%2FcJpR90pts%3D&reserved=0) 
R: We published code and data on the Zenodo platform. Since additional analysis were conducted after the first round of reviews, we updated the code and data [(https://zenodo.org/records/19628910)](https://doi.org/10.5281/zenodo.19628910)

* PLOS defines the “minimal data set” to consist of the data set used to reach the conclusions drawn in the manuscript with related metadata and methods, and any additional data required to replicate the reported study findings in their entirety. Authors do not need to submit their entire data set, or the raw data collected during an investigation. Please submit the following data:

The values behind the means, standard deviations and other measures reported;
The values used to build graphs;
The points extracted from images for analysis."
R: The minimal dataset and the corresponding code required to reproduce the analyses have been made publicly available on Zenodo platform ([https://zenodo.org/records/19628910](https://doi.org/10.5281/zenodo.19628910))

* Please provide titles and legends for all figures and tables (including those in Supporting Information files). Please define all acronyms used in each figure or table in its corresponding legend.
R: We confirm that the formatting is correct.

* Please ensure that where relevant figures include 95% CIs.

R: We confirm that the formatting is correct.

* When a p value is given, please specify the statistical test used to determine it in the legend.
R: We have added the corresponding statistical tests in the figure legends where p-values are reported.

* Please consider avoiding the use of red and green in order to make your figure more accessible
R: We have modified the figure where possible to avoid the use of red and green and improve accessibility.
HYPERLINK "https://eur03.safelinks.protection.outlook.com/?url=http%3A%2F%2Fwww.equator-network.org%2Freporting-guidelines%2Fprisma%2F&data=05%7C02%7Cluigi.russo12%40icatt.it%7C100bb5caf3c2422dc3f408de961f94f5%7C080496d6a52847a18c2af8860f43342f%7C0%7C0%7C639113259575511704%7CUnknown%7CTWFpbGZsb3d8eyJFbXB0eU1hcGkiOnRydWUsIlYiOiIwLjAuMDAwMCIsIlAiOiJXaW4zMiIsIkFOIjoiTWFpbCIsIldUIjoyfQ%3D%3D%7C0%7C%7C%7C&sdata=G6YDB8ZDZem9YkWvHJyhC0SvdVS1RlYIkjMiW7q2v0U%3D&reserved=0"

* Where data points are discrete, please ensure that they are depicted in the figures as discrete data and not as a continuous line.
R: We confirm that the formatting is correct.

* Please provide the unadjusted comparisons as well as the adjusted comparisons in all relevant Tables
R: It was not applicable.

* Please specify the variables controlled for in all relevant Tables
R: It was not applicable

* Please consider if moving some of the figures shared in the supplementary information into the main text would aid the reader.
R: We moved PRISMA flowchart as figure 1 (previously S1 Figure) and metanalysis of PFSratio as new figure 4 (previously S2 Figure).

* Please include in the Acknowledgments recognition of individuals who played a role in data collection or participant care or involvement
R: We included all the necessary individuals in the acknowledgements section.

* Please report your SR/MA according to the PRISMA guidelines provided at the EQUATOR site.

[https://eur03.safelinks.protection.outlook.com/?url=http%3A%2F%2Fwww.equator-network.org%2Freporting-guidelines%2Fprisma%2F&data=05%7C02%7Cnicolo.lentini%40unicatt.it%7C28d246d00e504765f20708de961d9e9b%7Cb94f7d7481ff44a9b5886682acc85779%7C0%7C0%7C639113251132465902%7CUnknown%7CTWFpbGZsb3d8eyJFbXB0eU1hcGkiOnRydWUsIlYiOiIwLjAuMDAwMCIsIlAiOiJXaW4zMiIsIkFOIjoiTWFpbCIsIldUIjoyfQ%3D%3D%7C0%7C%7C%7C&sdata=sr4bKYuuTW1lDatWF3qrppQmWZVFKhRi6nKQFd5kv5Y%3D&reserved=0](https://eur03.safelinks.protection.outlook.com/?url=http%3A%2F%2Fwww.equator-network.org%2Freporting-guidelines%2Fprisma%2F&data=05%7C02%7Cluigi.russo12%40icatt.it%7C100bb5caf3c2422dc3f408de961f94f5%7C080496d6a52847a18c2af8860f43342f%7C0%7C0%7C639113259575511704%7CUnknown%7CTWFpbGZsb3d8eyJFbXB0eU1hcGkiOnRydWUsIlYiOiIwLjAuMDAwMCIsIlAiOiJXaW4zMiIsIkFOIjoiTWFpbCIsIldUIjoyfQ%3D%3D%7C0%7C%7C%7C&sdata=G6YDB8ZDZem9YkWvHJyhC0SvdVS1RlYIkjMiW7q2v0U%3D&reserved=0)

Please provide the completed PRISMA checklist as Supporting Information.
When completing the checklist, please use section and paragraph numbers, rather than page numbers.
Please add the following statement, or similar, to the Methods: ""This study is reported as per the Preferred Reporting Items for Systematic Reviews and Meta-Analyses (PRISMA) guideline (S1 Checklist)."""
R: The statement in the manuscript and the PRISMA checklist as Supporting Information have been included.

* Abstract: Please report your abstract according to PRISMA for abstracts (https://doi.org/10.1371/journal.pmed.1001419) following the PLOS Medicine abstract structure (Background, Methods and Findings, Conclusions). Please ensure you provide dates of search, data sources, number of studies included, types of study designs included, eligibility criteria, and synthesis/appraisal methods.

R: We confirm the abstract is formatted in a compliant way.

* PRISMA flow diagram must be in the main body of the manuscript as Figure 1. Please update manuscript accordingly.
R: We revised the manuscript accordingly.

* Please provide the beginning and end dates of your search.
R: Search was conducted from inception to 1^st^ may 2025.
* Please note that we expect searches to be updated to within 6 months of the time of submission.
R: the search was updated until 1^st^ may 2025 and exactly seven months passed from the search update to the submission. We hope this is still compliant with the request.

* Please provide and explanation for including English literature only

R: We chose to include only English-language literature to ensure consistency and feasibility in the review process. English is the predominant language of scientific publication in this field, which allows for broad coverage of high-quality and widely accessible studies. Additionally, limiting the inclusion to English-language articles reduces the risk of misinterpretation due to translation and ensures that all selected studies could be accurately assessed within the resources available.

* Please report your abstract according to PRISMA for abstracts, following the PLOS Medicine abstract structure (Background, Methods and Findings, Conclusions) [https://eur03.safelinks.protection.outlook.com/?url=http%3A%2F%2Fwww.plosmedicine.org%2Farticle%2Finfo%3Adoi%2F10.1371%2Fjournal.pmed.1001419&data=05%7C02%7Cnicolo.lentini%40unicatt.it%7C28d246d00e504765f20708de961d9e9b%7Cb94f7d7481ff44a9b5886682acc85779%7C0%7C0%7C639113251132483025%7CUnknown%7CTWFpbGZsb3d8eyJFbXB0eU1hcGkiOnRydWUsIlYiOiIwLjAuMDAwMCIsIlAiOiJXaW4zMiIsIkFOIjoiTWFpbCIsIldUIjoyfQ%3D%3D%7C0%7C%7C%7C&sdata=2CTYa5URsp2OHIZFbe0TaDCIbIde2aHwl3sR5MxWqbY%3D&reserved=0](https://eur03.safelinks.protection.outlook.com/?url=http%3A%2F%2Fwww.plosmedicine.org%2Farticle%2Finfo%3Adoi%2F10.1371%2Fjournal.pmed.1001419&data=05%7C02%7Cluigi.russo12%40icatt.it%7C100bb5caf3c2422dc3f408de961f94f5%7C080496d6a52847a18c2af8860f43342f%7C0%7C0%7C639113259575532603%7CUnknown%7CTWFpbGZsb3d8eyJFbXB0eU1hcGkiOnRydWUsIlYiOiIwLjAuMDAwMCIsIlAiOiJXaW4zMiIsIkFOIjoiTWFpbCIsIldUIjoyfQ%3D%3D%7C0%7C%7C%7C&sdata=Eh5iN0rBiOPC%2BXkS%2B3urP0XGev3BjE%2FH%2BLtODCM%2FTC4%3D&reserved=0) .
R: We confirm the abstract is formatted in a compliant way.

Comments from Reviewers:

Reviewer #1: The authors have revised appropriately with the exception of not removing the SHIVA trial (or adding TAPUR and MATCH).  Their rationale is that the SHIVA trial provided access to an MTB.  This is problematic because
1) The primary goal of SHIVA was to determine if the medications worked for mutations outside of their usual indications. The other studies were assessing the efficacy of MTBs.  It seems there should be a common goal of the studies included in a meta-analysis

R: We acknowledge that the primary objective of the SHIVA trial differed from that of most studies included in our meta-analysis, as it aimed to evaluate the efficacy of molecularly targeted agents beyond their approved indications. However, our inclusion criteria were not based on the primary endpoint of the study, but rather on the presence of a MTB, like structure involved in treatment decision-making. Importantly, as confirmed by the investigators of the SHIVA trial, all patients were discussed within a molecular biology board framework. While the trial incorporated a predefined treatment algorithm, this was not applied in isolation: cases, particularly those with multiple molecular alterations, were systematically reviewed by an expert board to guide treatment allocation. Even though we agree that the published report does not provide a detailed quantitative description of MTB discussions, based on clarification from the study investigators, MTB review was part of the clinical workflow for all the patient management within the trial, and, for this reason, we considered SHIVA to meet our predefined criterion of structured MTB involvement.

2) Its not clear how many individuals were reviewed by the MTB but in the meta-analysis all patients in the trial were considered to be reviewed by the MTB

R: In the SHIVA trail, all patients were reviewed in the MTB organized for the trial, as confirmed by one of the PI of the trial.

3)  The SHIVA trial had several well documented weakness, but most importantly was that the drugs in the algorithm were not effective in those mutations, for example the imatinib concentration required to inhibit RET is ~40 umol/L which is not clinically achievable

R: Concerning the known limitations of the SHIVA trial, including the biological and pharmacological limitations of certain drug–target matches (e.g., imatinib for RET alterations), we fully agree with the reviewer. These limitations have been widely discussed in the literature and represent an important historical benchmark in the evolution of precision oncology. Importantly, the aim of our meta-analysis was not to assess the efficacy of specific drug-target matches, but rather to evaluate the clinical impact of MTB-guided decision-making. For this purpose, SHIVA remains informative as an early example of integrating molecular profiling and expert review into treatment orientations.

Reviewer #2: Thank you to the authors for their detailed responses and for the substantial revisions made to the manuscript. The methodological transparency of the study has improved considerably, particularly with the clarification of the synthesis strategy by study design, the addition of meta-regression analyses, reporting of prediction intervals, and clearer description of effect estimate extraction and sensitivity analyses. These revisions address the major concerns raised in the previous review.
I have a few minor additional suggestions that could further strengthen the manuscript:
a) While prediction intervals are now reported, it would be useful to briefly interpret them in the Results or Discussion sections, particularly where they include the null value, as this reflects the expected range of effects in future studies.

R: We thank the reviewer for this important suggestion. We have now added a brief interpretation of the prediction intervals in the Discussion section, specifically highlighting instances in which they include the null value and discussing their implications for the expected range of effects in future studies.

b) Given that the largest effect sizes appear in retrospective studies, the Discussion could more explicitly emphasize the potential role of residual confounding and selection bias, and more clearly distinguish between causal inference from randomized trials and associative evidence from observational studies.

R: We thank the reviewer for this important comment. We believe that the potential impact of residual confounding and selection bias is already addressed at multiple points in the Discussion, and we aimed to avoid overemphasizing this single aspect. However, regarding the distinction between causal inference and associative evidence, we have added a sentence to more clearly highlight this point.

c) The limitations of study-level meta-regression (e.g., ecological bias and limited statistical power) could be acknowledged more explicitly.

R: We thank the reviewer for this valuable suggestion. We have now explicitly acknowledged the limitations of study-level meta-regression in the Discussion, including the potential for ecological bias and limited statistical power, particularly given the number of included studies.

d) It would be helpful to report how many hazard ratios were reconstructed from Kaplan-Meier curves and whether sensitivity analyses excluding reconstructed estimates were considered.

R: We thank the reviewer for this important comment. We have now reported that 20 hazard ratios were reconstructed from Kaplan-Meier curves (previously detailed in S3 Table) and clarified that sensitivity analyses excluding these reconstructed estimates were performed. These analyses confirmed the robustness of the findings, with no meaningful changes in the main results. The corresponding information has been added and updated in the Methods, Results, and Discussion sections.

Reviewer #4: Very important work, methodologically robust, limitations and biases were addressed.

R: We thank the reviewer for this thoughtful comment and for the positive appraisal of our work.
