# Supplementary material for: Molecular Tumor Boards clinical impact on patient care and structural features: A systematic review and meta-analysis
Source: PLoS Med. 2026 Jun 9;23(6):e1005125. doi: 10.1371/journal.pmed.1005125 (PMC13274928; doi:10.1371/journal.pmed.1005125)
Supplement: Attachment — Submitted filename: Response_3_MTB_PLOS.docx [file pmed.1005125.s009.docx]

Dear Dr. Lentini,

Thank you very much for re-submitting your manuscript "Molecular Tumor Boards clinical impact on patient care and structural features: a systematic review and metanalysis" (PMEDICINE-D-25-04203R3) for review by PLOS Medicine.

I have discussed the paper with my colleagues and the academic editor and it was also seen again by two reviewers. I am pleased to say that provided the remaining editorial and production issues are dealt with we are planning to accept the paper for publication in the journal.

The remaining issues that need to be addressed are listed at the end of this email. Any accompanying reviewer attachments can be seen via the link below. Please take these into account before resubmitting your manuscript:


***Please note while forming your response, if your article is accepted, you may have the opportunity to make the peer review history publicly available. The record will include editor decision letters (with reviews) and your responses to reviewer comments. If eligible, we will contact you to opt in or out.***

In revising the manuscript for further consideration here, please ensure you address the specific points made by each reviewer and the editors. In your rebuttal letter you should indicate your response to the reviewers' and editors' comments/requests and the changes you have made in the manuscript. Please submit a clean version of the paper as the main article file. A version with changes marked must also be uploaded as a marked up manuscript file.

In addition to these revisions, you may need to complete some formatting changes, which you will receive in a follow up email. A member of our team will be in touch with a set of requests shortly. If you do not receive a separate email within a few days, please assume that checks have been completed, and no additional changes are required.

We expect to receive your revised manuscript within 1 week. Please email us (plosmedicine@plos.org) if you have any questions or concerns.

Please review your reference list to ensure that it is complete and correct. If you have cited papers that have been retracted, please include the rationale for doing so in the manuscript text, or remove these references and replace them with relevant current references. Any changes to the reference list should be mentioned in the rebuttal letter that accompanies your revised manuscript.

Please note, when your manuscript is accepted, an uncorrected proof of your manuscript will be published online ahead of the final version, unless you've already opted out via the online submission form. If, for any reason, you do not want an earlier version of your manuscript published online or are unsure if you have already indicated as such, please let the journal staff know immediately at plosmedicine@plos.org.

If you have any questions in the meantime, please contact me or the journal staff on plosmedicine@plos.org.  

We look forward to receiving the revised manuscript by May 13 2026 11:59PM.   

Sincerely,

Katarzyna Starowicz, PhD
Associate Editor 
PLOS Medicine
plosmedicine.org

------------------------------------------------------------
Requests from Editors:

* Please correct the title in Manuscript file as well as in Editorial Manager and where applicable, second part of it should be "A systematic review and meta-analysis "

R: We corrected the title accordingly.

* I have discussed the matter of search updated and you need update it until 28th June 2025. Maybe the updated search won't even yield new results but please check for publications in missing period. I a case there are result we DO NOT ask you to re-do analysis, you will just need to acknowledge their existence in the text and update manuscript accordingly in method section and where applicable.
R: We thank the Editor for this suggestion. We updated the search strategy up to July 1st, 2025. This updated search identified three additional eligible observational studies. As requested, we did not repeat the analyses; however, we acknowledged the presence of these studies in the Methods section.

* There is a discrepancy in search dates between abstract (states March 2025) and Method section, please correct and check manuscript carefully for consistency of a new requested date.

R. We thank the Editor for pointing out this inconsistency. We have now corrected the search dates mettendola fino July 1st, 2025 in the abstract.

* As you will see below, reviewer #1 is still raising major point of not removing SHIVA trial or adding TAPUR and NCI. After discussing this with editorial team we would like you to confirm that all the publications in your SR/MA conform to your eligibility and exclusion criteria.
R. We thank the Editor for allowing us to confirm that all studies included in our systematic review and meta-analysis conform to our predefined eligibility and exclusion criteria.

* Please include rebuttal for editorial and reviewers requests in revised manuscript submission.
R. We have included rebuttals to all Editors’ and Reviewers’ comments in the revised submission.

Comments from Reviewers:

Reviewer #1: 1) Authors have declined to remove SHIVA, arguing that an MTB was available despite the drug-gene combination being fixed in the study design.  If that is the case, both TAPUR and NCI match should be included, as they had similar designs (at least as to the drug-gene combinations being fixed) to SHIVA and had MTBs. 
R: We understand the Reviewer’s point, as also acknowledged in the previous revision rounds. However, we still believe that these platform trials should not be considered eligible because, although the drug-gene combinations were predefined similarly to SHIVA, not every patient underwent systematic Molecular Tumor Board evaluation. In contrast, within SHIVA, each patient was systematically reviewed by the MTB before treatment allocation. We thank the Reviewer again for raising this important point, which has now been further clarified in the Methods section. We hope that, even if disagreement remains regarding this methodological aspect, the revised text improves the overall transparency and clarity of the manuscript.

Reviewer #2: Thank you for thoroughly addressing all my comments and providing detailed clarifications in your revised manuscript. I appreciate the effort you have put into enhancing the rigor and transparency of your analyses.

The manuscript now reflects a robust and valuable contribution to subject, I am happy to recommend this work for publication and look forward to seeing its positive impact in the field.
R: We sincerely thank the Reviewer for the positive evaluation of our revised manuscript and for the constructive comments provided throughout the review process.
