# Supplementary material for: Molecular Tumor Boards clinical impact on patient care and structural features: A systematic review and meta-analysis
Source: PLoS Med. 2026 Jun 9;23(6):e1005125. doi: 10.1371/journal.pmed.1005125 (PMC13274928; doi:10.1371/journal.pmed.1005125)
Supplement: Peer review history

# Editor report: Decision Letter 0

**DOI:** 10.1371/journal.pmed.1005125.r001

**Authors:** Simona Fiorani

4 Dec 2025

Dear Dr Lentini,

Thank you for submitting your manuscript entitled "Molecular Tumor Boards clinical impact on patient care and structural features: a systematic review and metanalysis" for consideration by PLOS Medicine.

Your manuscript has now been evaluated by the PLOS Medicine editorial staff, and I am writing to let you know that we would like to send your submission out for external peer review.

However, before we can send your manuscript to reviewers, we need you to complete your submission by providing the metadata that is required for full assessment. To this end, please login to Editorial Manager where you will find the paper in the 'Submissions Needing Revisions' folder on your homepage. Please click 'Revise Submission' from the Action Links and complete all additional questions in the submission questionnaire.

For clinical studies, please upload a copy of your trial study protocol as a supporting information file. The study protocol should be the version submitted for approval to the institutional review board or ethics committee, should include any amendments to the study protocol, as well as the date of their approval by the institutional review or ethics committee. Please also detail any deviations from the study protocol in the Methods section of your manuscript. The editors will consider the protocol and study conduct prior to a final decision for external review.

Please re-submit your manuscript within two working days, i.e. by Dec 08 2025 11:59PM.

Login to Editorial Manager here: https://www.editorialmanager.com/pmedicine

Once your full submission is complete, your paper will undergo a series of checks in preparation for peer review. Once your manuscript has passed all checks it will be sent out for review.

Feel free to email us at plosmedicine@plos.org if you have any queries relating to your submission.

Kind regards,

Simona Fiorani

Senior Editor

PLOS Medicine

# Peer review: Decision Letter 1

**DOI:** 10.1371/journal.pmed.1005125.r002

**Authors:** Katarzyna Starowicz

11 Feb 2026

Dear Dr Lentini,

Many thanks for submitting your manuscript "Molecular Tumor Boards clinical impact on patient care and structural features: a systematic review and metanalysis" (PMEDICINE-D-25-04203R1) to PLOS Medicine. The paper has been reviewed by subject experts and a statistician; their comments are included below and can also be accessed here: [LINK]

After discussing the paper with the editorial team and an academic editor with relevant expertise, I'm pleased to invite you to revise the paper in response to the reviewers' comments. As you will see, the reviewers find your study to be valuable for the field but they have raised some important concerns around methodology and requested some clarification, that we would ask you to address in full.

We plan to send the revised paper to some or all of the original reviewers and additionally to one extra subject expert for input, and we cannot provide any guarantees at this stage regarding publication.

In addition to these revisions, you may need to complete some formatting changes, which you will receive in a follow up email. A member of our team will be in touch with a set of requests shortly. If you do not receive a separate email within a few days, please assume that checks have been completed, and no additional changes are required.

When you upload your revision, please include a point-by-point response that addresses all of the reviewer and editorial points, indicating the changes made in the manuscript and either an excerpt of the revised text or the location (eg: page and line number) where each change can be found. Please also be sure to check the general editorial comments at the end of this letter and include these in your point-by-point response. When you resubmit your paper, please include a clean version of the paper as the main article file and a version with changes tracked as a marked-up manuscript. It may also be helpful to check the guidelines for revised papers at http://journals.plos.org/plosmedicine/s/revising-your-manuscript for any that apply to your paper.

We ask that you submit your revision by Mar 04 2026 11:59PM. However, if this deadline is not feasible, please contact me by email, and we can discuss a suitable alternative.

Don't hesitate to contact me directly with any questions (kstarowicz@plos.org).

Best regards,

Katarzyna

Katarzyna Starowicz, PhD

Associate Editor

PLOS Medicine

kstarowicz@plos.org

-----------------------------------------------------------

Comments from the reviewers:

Reviewer #1: The authors have provided a comprehensive review and meta-analysis of the clinical efficacy of MTBs. Major positives include that it is the largest to date and the only one to stratify by study design. They also made intensive efforts to reach out to study PIs to obtain missing information.

Major concerns

1) The number of targeted therapies available have increased dramatically since 2015 when the SHIVA trial was reported (and 2012 when it was conceived). Could date of publication date be used to categorize trials as well?

2) The SHIVA trial had a predefined treatment algorithm that assigned a targeted therapy to a mutation in various pathways. The other studies included used an MTB team that made individual recommendations. The TAPUR and NCI-MATCH studies employed a similar approach to SHIVA (predefined drug/mutation assignment) and not included. Trials like SHIVA with prespecified treatments should be excluded.

A few suggestions

1) page 13, I don't think OS was consistently favored in the MTB guided across study designs. It was more of a trend in the randomized designs. Please revise

2) The abstract conclusion is very well stated. I suggest revising the discussion to better support the conclusion by including a para on the need for high quality studies, and potentially point out that the lowest quality studies (observational) had the best outcomes as well as a para on the dilemmas facing health systems with regard to MTBs (is the effort worth the outcomes)

Reviewer #2: Major comments

1) The review combines randomised controlled trials, non-randomised clinical trials, and observational (prospective and retrospective) studies within the same quantitative framework. Although stratification by study design is presented in figures and sensitivity analyses, the manuscript does not sufficiently justify the appropriateness of pooling these fundamentally different designs. Given the high risk of confounding and selection bias in non-randomised and observational studies, a clearer rationale is needed for the chosen synthesis strategy, including an explicit discussion of causal estimands and whether the pooled effects are intended to represent causal or associative effects.

2) The definition and use of the Progression-Free Survival (PFS) ratio (≥1.3) as a binary outcome raises methodological concerns. This metric is highly sensitive to the choice of prior line of therapy and assumes comparability between pre- and post-MTB treatment periods, which is unlikely across heterogeneous clinical contexts. The manuscript should more clearly justify the statistical validity of this threshold-based outcome and discuss potential biases such as regression to the mean, immortal time bias, and informative censoring, particularly in observational settings.

3) Survival outcomes (OS and PFS) are synthesised despite substantial heterogeneity in the definition of time origin across studies (e.g., randomisation, treatment initiation, MTB discussion, diagnosis, or consent). Although sensitivity analyses restricted to studies measuring survival from treatment initiation are presented, this issue remains a major threat to internal validity. The primary analyses should more explicitly acknowledge that pooled hazard ratios may not be directly comparable across studies with different time zero definitions, and stronger emphasis should be placed on these sensitivity analyses in the main results and interpretation.

4) The manuscript reports heterogeneity statistics (I²) but provides limited exploration of its sources beyond stratification by study design. Given the wide clinical and methodological heterogeneity across included studies (tumor types, prior lines of therapy, MTB composition, comparator definitions, sequencing technologies), additional exploratory analyses (e.g., meta-regression or subgroup analyses by key effect modifiers) would be methodologically appropriate, or at minimum, the limitations arising from unaddressed heterogeneity should be discussed more explicitly.

5) For non-randomised and observational studies with comparators, it is unclear whether the extracted effect estimates are adjusted or unadjusted, and how differences in covariate adjustment across studies were handled in the meta-analysis. Pooling adjusted and unadjusted estimates without clear distinction may bias the summary effects. The Methods should clarify which estimates were preferentially extracted and whether any hierarchy or standardization was applied.

6) Risk-of-bias assessments are presented using appropriate tools, but their integration into the quantitative synthesis is limited. While sensitivity analyses excluding studies at serious or critical risk of bias are provided, the main pooled estimates still include these studies. The manuscript would benefit from a clearer explanation of how risk of bias influenced interpretation of the primary findings, particularly for outcomes dominated by observational evidence.

7) The certainty of evidence assessment (e.g., GRADE or equivalent) is mentioned but remains difficult to interpret in light of the extensive heterogeneity, mixed study designs, and outcome definition issues. The rationale for rating decisions should be more transparent, especially for outcomes where statistically significant pooled effects are driven primarily by observational retrospective studies.

Minor comments

8) It should be clarified whether random-effects models were used uniformly across all syntheses and, if so, which estimator (e.g., DerSimonian-Laird, REML) was applied. Justification for the chosen approach would improve methodological transparency.

9) Funnel plots are presented stratified by study design; however, their interpretability is limited in strata with a small number of studies. This limitation should be acknowledged more explicitly, and strong conclusions regarding publication bias should be avoided.

10) For binary outcomes such as ORR and DCR, the handling of zero-event studies and continuity corrections should be described, as these choices can materially affect pooled relative risks.

11) The Methods section would benefit from a concise summary table linking each outcome to its effect measure, eligible study designs, and main sensitivity analyses, to improve clarity for readers.

12) The manuscript should ensure consistent terminology when referring to "comparators," particularly distinguishing between standard-of-care, physician's choice, non-targeted therapy, and absence of actionable alterations, as these are not methodologically equivalent comparison groups.

Reviewer #3: Summary and general assessment

The authors aim to evaluate whether receiving Molecular Tumor Board (MTB)-recommended treatments is associated with improved clinical outcomes, assessed using multiple effect measures, including hazard ratios (HRs) for overall survival (OS) and progression-free survival (PFS), relative risks (RRs) for objective response rate (ORR) and disease control rate (DCR), and pooled proportions for a PFS ratio ≥ 1.3. A total of 78 studies were identified through a comprehensive literature review, encompassing heterogeneous study designs, including randomized controlled trials (RCTs), non-randomized clinical trials, prospective studies, and retrospective studies.

The authors extracted general study information, MTB characteristics, patient and tumor characteristics, and clinical outcomes, including the starting point for survival time measurement. In addition, they contacted principal investigators of RCTs to obtain clarifications and additional data where needed.

For each study design category, pooled effect sizes were estimated using random-effects meta-analysis and reported with 95% confidence intervals. Prediction intervals were not reported. Across most outcomes and study designs, MTB-guided treatment was associated with improved clinical outcomes compared with non-MTB approaches.

Given the substantial heterogeneity across studies — including differences in cancer types and study design — the authors appropriately conducted sensitivity analyses, including leave-one-out analyses and exclusion of studies at high risk of bias. These analyses demonstrated that the direction of the pooled estimates remained stable, supporting the robustness of the overall findings. The authors also assessed potential publication bias and performed tests for excess significance, finding no clear evidence of publication bias.

Heterogeneity and meta-regression

To explore potential sources of between-study heterogeneity, the authors performed meta-regression analyses using study-level covariates. Notably, for OS, studies focusing on a single cancer type were associated with an odds ratio of 0.88 and accounted for 78.4% of the between-study variance, while for ORR this covariate explained 59.3% of the variance.

These findings suggest that whether a study focuses on a single versus multiple cancer types may be a major contributor to heterogeneity in reported outcomes. However, the manuscript does not clearly specify whether the meta-regression analyses were conducted jointly across all study designs with study design included as a covariate, or separately within each study-design category. Given the substantial differences in effect sizes and risk of bias across study designs, clarifying this point would be important for the interpretation of the meta-regression results.

Risk of bias and limitations

The authors assessed risk of bias using RoB 2 for randomized controlled trials and ROBINS-I for non-randomized studies. As expected, the majority of non-randomized studies were judged to have moderate to high risk of bias, whereas RCTs generally showed lower risk of bias.

The authors appropriately acknowledge that the overall evidence base is dominated by non-randomized studies with substantial risk of bias, which limits causal interpretation of the pooled estimates. This limitation is clearly stated and appropriately discussed.

Additional comments

Major comments

1. Prediction intervals

Given the substantial heterogeneity across studies, reporting prediction intervals in addition to confidence intervals would provide clinically relevant information on the expected range of effects in future studies.

2. Meta-regression strategy clarity

The manuscript would benefit from clearer reporting of the meta-regression strategy, including whether analyses were performed separately by study design or jointly across all study types, and whether covariates were included in univariable or multivariable models. In addition, it would be helpful to report the associations of study design, year of publication, and other study-level covariates with overall survival, as mentioned in the "Data synthesis and statistical methods" section.

3. Study inclusion and exclusion

On page 13, the authors state that "of the 78 studies identified, 52 were included in the meta-analysis." Could the authors clarify the reasons for exclusion of the remaining 26 studies?

4. Median OS reporting inconsistencies

On page 13 (OS section), median overall survival is reported for randomized controlled trials; however, this information is provided for only four studies, with one study missing. Additionally, in Supplementary Table 4, the median OS for the study by Schneider (2021) is missing. Clarification or completion of these data would improve transparency.

Minor comments

1. Page 5: In the sentence "A multidisciplinary genomic pathway is currently being developed," the term "pathway" may be confusing for readers with a biological background, as it commonly refers to molecular signaling pathways. Consider clarifying or rephrasing this term.

2. Page 5: In the sentence "We aim to evaluate the quality of evidence in order to district potential source of bias to make informed considerations," please consider replacing "district" with "identify."

3. Page 9: The sentence "For the analysis of patients with a PFS ratio ≥ 1.3, we calculated pooled proportions using …" could be revised for clarity, for example by explicitly stating that the pooled proportion was estimated to quantify the proportion of patients achieving a PFS ratio ≥ 1.3.

4. Page 17: Please consider revising the sentence "The results clearly show a trend versus significant effects on …" to clarify the contrast between randomized and observational evidence and to avoid the phrasing "trend versus significant effects."

Any attachments provided with reviews can be seen via the following link: [LINK]

--------------------------------------------------------- ---

General editorial requests:

(Note: not all will apply to your paper, but please check each item carefully)

* We ask every co-author listed on the manuscript to fill in a contributing author statement, making sure to declare all competing interests. If any of the co-authors have not filled in the statement, we will remind them to do so when the paper is revised. If all statements are not completed in a timely fashion this could hold up the re-review process. If new competing interests are declared later in the revision process, this may also hold up the submission. Should there be a problem getting one of your co-authors to fill in a statement we will be in contact. Please do not add or remove authors without first discussing this with the handling editor. You can see our competing interests policy here: http://journals.plos.org/plosmedicine/s/competing-interests.

* Please upload any figures associated with your paper as individual TIF or EPS files with 300dpi resolution at resubmission; please read our figure guidelines for more information on our requirements: http://journals.plos.org/plosmedicine/s/figures. While revising your submission, we strongly recommend that you use PLOS's NAAS tool (https://ngplosjournals.pagemajik.ai/artanalysis) to test your figure files. NAAS can convert your figure files to the TIFF file type and meet basic requirements (such as print size, resolution), or provide you with a report on issues that do not meet our requirements and that NAAS cannot fix.

After uploading your figures to PLOS's NAAS tool - https://ngplosjournals.pagemajik.ai/artanalysis, NAAS will process the files provided and display the results in the "Uploaded Files" section of the page as the processing is complete.

If the uploaded figures meet our requirements (or NAAS is able to fix the files to meet our requirements), the figure will be marked as "fixed" above. If NAAS is unable to fix the files, a red "failed" label will appear above.

When NAAS has confirmed that the figure files meet our requirements, please download the file via the download option, and include these NAAS processed figure files when submitting your revised manuscript.

* Please ensure that the paper adheres to the PLOS Data Availability Policy (see http://journals.plos.org/plosmedicine/s/data-availability), which requires that all data underlying the study's findings be provided in a repository or as Supporting Information. For data residing with a third party, authors are required to provide instructions with contact information (web or email address) for obtaining the data. Please note that a study author cannot be the contact person for the data. PLOS journals do not allow statements supported by "data not shown" or "unpublished results." For such statements, authors must provide supporting data or cite public sources that include it.

* We expect all researchers with submissions to PLOS in which author-generated code underpins the findings in the manuscript to make all author-generated code available without restrictions upon publication of the work. In cases where code is central to the manuscript, we may require the code to be made available as a condition of publication. Authors are responsible for ensuring that the code is reusable and well documented. Please make any custom code available, either as part of your data deposition or as a supplementary file. Please add a sentence to your data availability statement regarding any code used in the study, e.g. "The code used in the analysis is available from Github [URL] and archived in Zenodo [DOI link]" Please review our guidelines at https://journals.plos.org/plosmedicine/s/materials-software-and-code-sharing and ensure that your code is shared in a way that follows best practice and facilitates reproducibility and reuse. Because Github depositions can be readily changed or deleted, we encourage you to make a permanent DOI'd copy (e.g. in Zenodo) and provide the URL.

* Please ensure that the study is reported according to the PRISMA guideline and include the completed checklist as Supporting Information. When completing the checklist, please use section and paragraph numbers, rather than page numbers. Please add the following statement, or similar, to the Methods: "This study is reported as per [XXXX] guideline (S1 Checklist)."

FORMATTING - GENERAL

* Abstract: Please structure your abstract using the PLOS Medicine headings (Background, Methods and Findings, Conclusions). Please combine the Methods and Findings sections into one section.

* At this stage, we ask that you include a short, non-technical Author Summary of your research to make findings accessible to a wide audience that includes both scientists and non-scientists. The Author Summary should immediately follow the Abstract in your revised manuscript. This text is subject to editorial change and should be distinct from the scientific abstract. Ideally each sub-heading should contain 2-3 single sentence, concise bullet points containing the most salient points from your study. In the final bullet point of 'What Do These Findings Mean?', please include the main limitations of the study in non-technical language. Please see our author guidelines for more information: https://journals.plos.org/plosmedicine/s/revising-your-manuscript#loc-author-summary.

* Please express the main results with 95% CIs as well as p values. When reporting p values please report as p<0.001 and where higher as the exact p value p=0.002, for example. Throughout, suggest reporting statistical information as follows to improve clarity for the reader "22% (95% CI [13%,28%]; p</=)". Please be sure to define all numerical values at first use.

* Please include page numbers and line numbers in the manuscript file. Use continuous line numbers (do not restart the numbering on each page).

* Please cite the reference numbers in square brackets. Citations should precede punctuation.

FIGURES AND TABLES

* Please provide titles and legends for all figures and tables (including those in Supporting Information files).

* Please define all abbreviations used in each figure/table (including those in Supporting Information files).

* Please consider avoiding the use of red and green in order to make your figure more accessible to those with color blindness.

SUPPLEMENTARY MATERIAL

* Please note that supplementary material will be posted as supplied by the authors. Therefore, please amend it according to the relevant comments outlined here.

* Please cite your Supporting Information as outlined here: https://journals.plos.org/plosmedicine/s/supporting-information

REFERENCES

* PLOS uses the numbered citation (citation-sequence) method and first six authors, et al.

* Please ensure that journal name abbreviations match those found in the National Center for Biotechnology Information (NCBI) databases (http://www.ncbi.nlm.nih.gov/nlmcatalog/journals), and are appropriately formatted and capitalised.

* Where website addresses are cited, please include the complete URL and specify the date of access (e.g. [accessed: 12/06/2023]).

* Please also see https://journals.plos.org/plosmedicine/s/submission-guidelines#loc-references for further details on reference formatting.

STUDY TYPE-SPECIFIC REQUESTS

SYSTEMATIC REVIEWS & META-ANALYSES

* Please report your SR/MA according to the PRISMA guidelines provided at the EQUATOR site. http://www.equator-network.org/reporting-guidelines/prisma/. Please provide the completed PRISMA checklist as Supporting Information. When completing the checklist, please use section and paragraph numbers, rather than page numbers. Please add the following statement, or similar, to the Methods: "This study is reported as per the Preferred Reporting Items for Systematic Reviews and Meta-Analyses (PRISMA) guideline (S1 Checklist)."

* Abstract: Please report your abstract according to PRISMA for abstracts (https://doi.org/10.1371/journal.pmed.1001419) following the PLOS Medicine abstract structure (Background, Methods and Findings, Conclusions). Please ensure you provide dates of search, data sources, number of studies included, types of study designs included, eligibility criteria, and synthesis/appraisal methods.

* Please note that we expect searches to be updated to within 6 months of the time of submission.

* Please provide and explanation for including English literature only

# Author comment: Author response to Decision Letter 2

**DOI:** 10.1371/journal.pmed.1005125.r003

3 Mar 2026

Attachment Submitted filename: PLOS Response.docx

# Peer review: Decision Letter 2

**DOI:** 10.1371/journal.pmed.1005125.r004

**Authors:** Katarzyna Starowicz

9 Apr 2026

Dear Dr. Lentini,

Thank you very much for re-submitting your manuscript "Molecular Tumor Boards clinical impact on patient care and structural features: a systematic review and metanalysis" (PMEDICINE-D-25-04203R2) for review by PLOS Medicine.

I have discussed the paper with my colleagues and the academic editor and it was also seen again by three reviewers. I am pleased to say that provided the remaining editorial and production issues are dealt with we are planning to accept the paper for publication in the journal.

I did notice that you didn't provided a rebuttal for editorial comments and requests from previous decision letter. Please, include your responses to the points from previous decision letter followed by requests from this letter. Additionally, you have to provide point-to-point to reviewers comments. Without this we won't be able to proceed with the manuscript.

The remaining issues that need to be addressed are listed at the end of this email. Any accompanying reviewer attachments can be seen via the link below. Please take these into account before resubmitting your manuscript:

[LINK]

***Please note while forming your response, if your article is accepted, you may have the opportunity to make the peer review history publicly available. The record will include editor decision letters (with reviews) and your responses to reviewer comments. If eligible, we will contact you to opt in or out.***

In revising the manuscript for further consideration here, please ensure you address the specific points made by each reviewer and the editors. In your rebuttal letter you should indicate your response to the reviewers' and editors' comments and the changes you have made in the manuscript. Please submit a clean version of the paper as the main article file. A version with changes marked must also be uploaded as a marked up manuscript file.

Please also check the guidelines for revised papers at http://journals.plos.org/plosmedicine/s/revising-your-manuscript for any that apply to your paper. If you haven't already, we ask that you provide a short, non-technical Author Summary of your research to make findings accessible to a wide audience that includes both scientists and non-scientists. The Author Summary should immediately follow the Abstract in your revised manuscript. This text is subject to editorial change and should be distinct from the scientific abstract.

In addition to these revisions, you may need to complete some formatting changes, which you will receive in a follow up email. A member of our team will be in touch with a set of requests shortly. If you do not receive a separate email within a few days, please assume that checks have been completed, and no additional changes are required.

We expect to receive your revised manuscript within 1 week. Please email us (kstarowicz@plos.org or plosmedicine@plos.org) if you have any questions or concerns.

We ask every co-author listed on the manuscript to fill in a contributing author statement. If any of the co-authors have not filled in the statement, we will remind them to do so when the paper is revised. If all statements are not completed in a timely fashion this could hold up the re-review process. Should there be a problem getting one of your co-authors to fill in a statement we will be in contact. YOU MUST NOT ADD OR REMOVE AUTHORS UNLESS YOU HAVE ALERTED THE EDITOR HANDLING THE MANUSCRIPT TO THE CHANGE AND THEY SPECIFICALLY HAVE AGREED TO IT.

Please ensure that the paper adheres to the PLOS Data Availability Policy (see http://journals.plos.org/plosmedicine/s/data-availability), which requires that all data underlying the study's findings be provided in a repository or as Supporting Information. For data residing with a third party, authors are required to provide instructions with contact information for obtaining the data. PLOS journals do not allow statements supported by "data not shown" or "unpublished results." For such statements, authors must provide supporting data or cite public sources that include it.

To enhance the reproducibility of your results, we recommend that you deposit your laboratory protocols in protocols.io, where a protocol can be assigned its own identifier (DOI) such that it can be cited independently in the future. Additionally, PLOS ONE offers an option to publish peer-reviewed clinical study protocols. Read more information on sharing protocols at https://plos.org/protocols?utm_medium=editorial-email&utm_source=authorletters&utm_campaign=protocols

Please review your reference list to ensure that it is complete and correct. If you have cited papers that have been retracted, please include the rationale for doing so in the manuscript text, or remove these references and replace them with relevant current references. Any changes to the reference list should be mentioned in the rebuttal letter that accompanies your revised manuscript.

Please note, when your manuscript is accepted, an uncorrected proof of your manuscript will be published online ahead of the final version, unless you've already opted out via the online submission form. If, for any reason, you do not want an earlier version of your manuscript published online or are unsure if you have already indicated as such, please let the journal staff know immediately at plosmedicine@plos.org.

If you have any questions in the meantime, please contact me or the journal staff on plosmedicine@plos.org.

We look forward to receiving the revised manuscript by Apr 16 2026 11:59PM.

Sincerely,

Katarzyna Starowicz, PhD

Associate Editor

PLOS Medicine

kstarowicz@plos.org

------------------------------------------------------------

Requests from Editors:

* At this stage, we ask that you include a short, non-technical Author Summary of your research to make findings accessible to a wide audience that includes both scientists and non-scientists. The Author Summary should immediately follow the Abstract in your revised manuscript. This text is subject to editorial change and should be distinct from the scientific abstract. Ideally each sub-heading should contain 2-3 single sentence, concise bullet points containing the most salient points from your study. In the final bullet point of ‘What Do These Findings Mean?’ Please include the main limitations of the study in non-technical language.

Please see our author guidelines for more information: https://journals.plos.org/plosmedicine/s/revising-your-manuscript#loc-author-summary."

* Please confirm that your title complies with PLOS Medicine's style. Your title must be nondeclarative and not a question. It should begin with main concept if possible. "Effect of" should be used only if causality can be inferred, i.e., for an RCT. Please place the study design ("A randomized controlled trial," "A retrospective study," "A modelling study," etc.) in the subtitle (ie, after a colon).

* Please confirm that your abstract complies with our requirements, including format (three sections: Background, Methods and Findings, and Conclusions) and providing all the information relevant to this study type https://journals.plos.org/plosmedicine/s/submission-guidelines#loc-abstract

* Please ensure that the Introduction ends with a clear description of the study question or hypothesis.

* Please ensure that all abbreviations are defined at first use throughout the text.

* Please confirm that all numbers presented in the abstract are present and identical to numbers presented in the main manuscript text.

* Please review your text for claims of novelty or primacy (e.g. 'for the first time') and remove this language. In addition, please check that any use of statistical terms (such as trend or significant) are supported by the data, and if not please remove them.

* Please remove the 'conclusions' subheading from the discussion. Please also remove any other subheadings from the discussion.

* Statistical reporting: Please revise throughout the manuscript, including tables and figures.

- Please report statistical information as follows to improve clarity for the reader ""22% (95% CI [13,28]; p</=)"".

- Please separate upper and lower bounds with commas instead of hyphens as the latter can be confused with reporting of negative values.

- Please repeat statistical definitions (HR, CI etc.) for each set of parentheses."

* In the last sentence of the Abstract Methods and Findings section, please describe the main limitation(s) of the study's methodology.

* In the abstract, please include the important dependent variables that are adjusted for in the analyses.

* In the author summary, in the final bullet point of 'What Do These Findings Mean?', please include the main limitations of the study in non-technical language.

* PLOS Medicine requires that the de-identified data underlying the specific results in a published article be made available, without restrictions on access, in a public repository or as Supporting Information at the time of article publication, provided it is legal and ethical to do so. Please see the policy at

http://journals.plos.org/plosmedicine/s/data-availability

and FAQs at

http://journals.plos.org/plosmedicine/s/data-availability#loc-faqs-for-data-policy "

* The funding statement should include: specific grant numbers, initials of authors who received each award, URLs to sponsors’ websites. Also, please state whether any sponsors or funders (other than the named authors) played any role in study design, data collection and analysis, the decision to publish, or preparation of the manuscript. If they had no role in the research, include this sentence: “The funders had no role in study design, data collection and analysis, decision to publish, or preparation of the manuscript.”

* All authors must declare their relevant competing interests per the PLOS policy, which can be seen here: https://journals.plos.org/plosmedicine/s/competing-interests For authors with ties to industry, please indicate whether any of the interests has a financial stake in the results of the current study.

* PLOS Medicine requires that the de-identified data underlying the specific results in a published article be made available, without restrictions on access, in a public repository or as Supporting Information at the time of article publication, provided it is legal and ethical to do so. Please see the policy at

http://journals.plos.org/plosmedicine/s/data-availability and FAQs at http://journals.plos.org/plosmedicine/s/data-availability#loc-faqs-for-data-policy

* PLOS defines the “minimal data set” to consist of the data set used to reach the conclusions drawn in the manuscript with related metadata and methods, and any additional data required to replicate the reported study findings in their entirety. Authors do not need to submit their entire data set, or the raw data collected during an investigation. Please submit the following data:

The values behind the means, standard deviations and other measures reported;

The values used to build graphs;

The points extracted from images for analysis."

* Please provide titles and legends for all figures and tables (including those in Supporting Information files). Please define all acronyms used in each figure or table in its corresponding legend.

* Please ensure that where relevant figures include 95% CIs.

* When a p value is given, please specify the statistical test used to determine it in the legend.

* Please consider avoiding the use of red and green in order to make your figure more accessible

* Where data points are discrete, please ensure that they are depicted in the figures as discrete data and not as a continuous line.

* Please provide the unadjusted comparisons as well as the adjusted comparisons in all relevant Tables

* Please specify the variables controlled for in all relevant Tables

* Please consider if moving some of the figures shared in the supplementary information into the main text would aid the reader.

* Please include in the Acknowledgments recognition of individuals who played a role in data collection or participant care or involvement

* Please report your SR/MA according to the PRISMA guidelines provided at the EQUATOR site.

http://www.equator-network.org/reporting-guidelines/prisma/

Please provide the completed PRISMA checklist as Supporting Information.

When completing the checklist, please use section and paragraph numbers, rather than page numbers.

Please add the following statement, or similar, to the Methods: ""This study is reported as per the Preferred Reporting Items for Systematic Reviews and Meta-Analyses (PRISMA) guideline (S1 Checklist)."""

* PRISMA flow diagram must be in the main body of the manuscript as Figure 1. Please update manuscript accordingly.

* Please provide the beginning and end dates of your search.

* Please note that we expect searches to be updated to within 6 months of the time of submission.

* Please provide and explanation for including English literature only

* Please report your abstract according to PRISMA for abstracts, following the PLOS Medicine abstract structure (Background, Methods and Findings, Conclusions) http://www.plosmedicine.org/article/info:doi/10.1371/journal.pmed.1001419 .

Comments from Reviewers:

Reviewer #1: The authors have revised appropriately with the exception of not removing the SHIVA trial (or adding TAPUR and MATCH). Their rationale is that the SHIVA trial provided access to an MTB. This is problematic because

1) The primary goal of SHIVA was to determine if the medications worked for mutations outside of their usual indications. The other studies were assessing the efficacy of MTBs. It seems there should be a common goal of the studies included in a meta-analysis

2) Its not clear how many individuals were reviewed by the MTB but in the meta-analysis all patients in the trial were considered to be reviewed by the MTB

3) The SHIVA trial had several well documented weakness, but most importantly was that the drugs in the algorithm were not effective in those mutations, for example the imatinib concentration required to inhibit RET is ~40 umol/L which is not clinically achievable

Reviewer #2: Thank you to the authors for their detailed responses and for the substantial revisions made to the manuscript. The methodological transparency of the study has improved considerably, particularly with the clarification of the synthesis strategy by study design, the addition of meta-regression analyses, reporting of prediction intervals, and clearer description of effect estimate extraction and sensitivity analyses. These revisions address the major concerns raised in the previous review.

I have a few minor additional suggestions that could further strengthen the manuscript:

a) While prediction intervals are now reported, it would be useful to briefly interpret them in the Results or Discussion sections, particularly where they include the null value, as this reflects the expected range of effects in future studies.

b) Given that the largest effect sizes appear in retrospective studies, the Discussion could more explicitly emphasize the potential role of residual confounding and selection bias, and more clearly distinguish between causal inference from randomized trials and associative evidence from observational studies.

c) The limitations of study-level meta-regression (e.g., ecological bias and limited statistical power) could be acknowledged more explicitly.

d) It would be helpful to report how many hazard ratios were reconstructed from Kaplan-Meier curves and whether sensitivity analyses excluding reconstructed estimates were considered.

Reviewer #4: Very important work, methodologically robust, limitations and biases were addressed.

Any attachments provided with reviews can be seen via the following link:

[LINK]

# Author comment: Author response to Decision Letter 3

**DOI:** 10.1371/journal.pmed.1005125.r005

17 Apr 2026

Attachment Submitted filename: Response_2_MTB_PLOS.docx

# Peer review: Decision Letter 3

**DOI:** 10.1371/journal.pmed.1005125.r006

**Authors:** Katarzyna Starowicz

6 May 2026

Dear Dr. Lentini,

Thank you very much for re-submitting your manuscript "Molecular Tumor Boards clinical impact on patient care and structural features: a systematic review and metanalysis" (PMEDICINE-D-25-04203R3) for review by PLOS Medicine.

I have discussed the paper with my colleagues and the academic editor and it was also seen again by two reviewers. I am pleased to say that provided the remaining editorial and production issues are dealt with we are planning to accept the paper for publication in the journal.

The remaining issues that need to be addressed are listed at the end of this email. Any accompanying reviewer attachments can be seen via the link below. Please take these into account before resubmitting your manuscript:

[LINK]

***Please note while forming your response, if your article is accepted, you may have the opportunity to make the peer review history publicly available. The record will include editor decision letters (with reviews) and your responses to reviewer comments. If eligible, we will contact you to opt in or out.***

In revising the manuscript for further consideration here, please ensure you address the specific points made by each reviewer and the editors. In your rebuttal letter you should indicate your response to the reviewers' and editors' comments/requests and the changes you have made in the manuscript. Please submit a clean version of the paper as the main article file. A version with changes marked must also be uploaded as a marked up manuscript file.

In addition to these revisions, you may need to complete some formatting changes, which you will receive in a follow up email. A member of our team will be in touch with a set of requests shortly. If you do not receive a separate email within a few days, please assume that checks have been completed, and no additional changes are required.

We expect to receive your revised manuscript within 1 week. Please email us (plosmedicine@plos.org) if you have any questions or concerns.

Please review your reference list to ensure that it is complete and correct. If you have cited papers that have been retracted, please include the rationale for doing so in the manuscript text, or remove these references and replace them with relevant current references. Any changes to the reference list should be mentioned in the rebuttal letter that accompanies your revised manuscript.

Please note, when your manuscript is accepted, an uncorrected proof of your manuscript will be published online ahead of the final version, unless you've already opted out via the online submission form. If, for any reason, you do not want an earlier version of your manuscript published online or are unsure if you have already indicated as such, please let the journal staff know immediately at plosmedicine@plos.org.

If you have any questions in the meantime, please contact me or the journal staff on plosmedicine@plos.org.

We look forward to receiving the revised manuscript by May 13 2026 11:59PM.

Sincerely,

Katarzyna Starowicz, PhD

Associate Editor

PLOS Medicine

plosmedicine.org

------------------------------------------------------------

Requests from Editors:

* Please correct the title in Manuscript file as well as in Editorial Manager and where applicable, second part of it should be "A systematic review and meta-analysis "

* I have discussed the matter of search updated and you need update it until 28th June 2025. Maybe the updated search won't even yield new results but please check for publications in missing period. I a case there are result we DO NOT ask you to re-do analysis, you will just need to acknowledge their existence in the text and update manuscript accordingly in method section and where applicable.

* There is a discrepancy in search dates between abstract (states March 2025) and Method section, please correct and check manuscript carefully for consistency of a new requested date.

* As you will see below, reviewer #1 is still raising major point of not removing SHIVA trial or adding TAPUR and NCI. After discussing this with editorial team we would like you to confirm that all the publications in your SR/MA conform to your eligibility and exclusion criteria.

* Please include rebuttal for editorial and reviewers requests in revised manuscript submission.

Comments from Reviewers:

Reviewer #1: 1) Authors have declined to remove SHIVA, arguing that an MTB was available despite the drug-gene combination being fixed in the study design. If that is the case, both TAPUR and NCI match should be included, as they had similar designs (at least as to the drug-gene combinations being fixed) to SHIVA and had MTBs.

Reviewer #2: Thank you for thoroughly addressing all my comments and providing detailed clarifications in your revised manuscript. I appreciate the effort you have put into enhancing the rigor and transparency of your analyses.

The manuscript now reflects a robust and valuable contribution to subject, I am happy to recommend this work for publication and look forward to seeing its positive impact in the field.

Any attachments provided with reviews can be seen via the following link:

[LINK]

# Author comment: Author response to Decision Letter 4

**DOI:** 10.1371/journal.pmed.1005125.r007

12 May 2026

Attachment Submitted filename: Response_3_MTB_PLOS.docx

# Editor report: Decision Letter 4

**DOI:** 10.1371/journal.pmed.1005125.r008

**Authors:** Katarzyna Starowicz

18 May 2026

Dear Dr Lentini,

On behalf of my colleagues and the Academic Editor, Dario Trapani, I am pleased to inform you that we have agreed to publish your manuscript "Molecular Tumor Boards clinical impact on patient care and structural features: a systematic review and meta-analysis" (PMEDICINE-D-25-04203R4) in PLOS Medicine.

Before your manuscript can be formally accepted you will need to complete some formatting changes, which you will receive in a follow up email. Please be aware that it may take several days for you to receive this email; during this time no action is required by you. Once you have received these formatting requests, please note that your manuscript will not be scheduled for publication until you have made the required changes.

In the meantime, please log into Editorial Manager at http://www.editorialmanager.com/pmedicine/, click the "Update My Information" link at the top of the page, and update your user information to ensure an efficient production process.

PRESS

We frequently collaborate with press offices. If your institution or institutions have a press office, please notify them about your upcoming paper at this point, to enable them to help maximise its impact. If the press office is planning to promote your findings, we would be grateful if they could coordinate with medicinepress@plos.org. If you have not yet opted out of the early version process, we ask that you notify us immediately of any press plans so that we may do so on your behalf.

We also ask that you take this opportunity to read our Embargo Policy regarding the discussion, promotion and media coverage of work that is yet to be published by PLOS. As your manuscript is not yet published, it is bound by the conditions of our Embargo Policy. Please be aware that this policy is in place both to ensure that any press coverage of your article is fully substantiated and to provide a direct link between such coverage and the published work. For full details of our Embargo Policy, please visit http://www.plos.org/about/media-inquiries/embargo-policy/.

To enhance the reproducibility of your results, we recommend that you deposit your laboratory protocols in protocols.io, where a protocol can be assigned its own identifier (DOI) such that it can be cited independently in the future. Additionally, PLOS ONE offers an option to publish peer-reviewed clinical study protocols. Read more information on sharing protocols at https://plos.org/protocols?utm_medium=editorial-email&utm_source=authorletters&utm_campaign=protocols

Thank you again for submitting to PLOS Medicine. We look forward to publishing your paper.

Sincerely,

Katarzyna Starowicz, PhD

Associate Editor

PLOS Medicine
